# Determining Acute-Phase Biomarkers for Mild Traumatic Brain Injury Through Exploratory Data Analysis: A Preliminary Report

**DOI:** 10.1007/s12035-026-06130-1

**Published:** 2026-09-25

**Authors:** João Luís Vieira Monteiro de Barros, Maíra Glória de Freitas Cardoso, Danielle Emely de Souza Almeida, Agnes Stéphanie da Silva, Caroline Amaral Machado, Bruna da Silva Oliveira, Isabella Alves Teixeira de Abreu, Patrícia Fernandes Fontes, Natalia Pessoa Rocha, Vinicius Sousa Pietra Pedroso, Rodrigo Moreira Faleiro, Érica Leandro Marciano Vieira, Antônio Lúcio Teixeira, Leonardo Cruz de Souza, Rafael Alves Bonfim de Queiroz, Aline Silva de Miranda

**Affiliations:** 1 Laboratório Interdisciplinar de Investigação Médica (LIIM), Faculdade de Medicina, Universidade Federal de Minas Gerais (UFMG), Belo Horizonte, MG Brazil; 2 Programa de Pós-Graduação em Biologia Celular da UFMG, Belo Horizonte, MG Brazil; 3 Programa de Pós-Graduação em Biotecnologia, Universidade Federal de Ouro Preto (UFOP), Ouro Preto, MG Brazil; 4 Laboratório de Neurobiologia, Departamento de Morfologia, Instituto de Ciências Biológicas, UFMG, Av. Pres. Antônio Carlos, 6627 - Pampulha, Belo Horizonte, MG 31270-901 Brazil; 5 Department of Neurology, McGovern Medical School, The University of Texas Health Science Center at Houston, Houston, TX USA; 6 Hospital João XXIII, Fundação Hospitalar do Estado de Minas Gerais – FHEMIG, Belo Horizonte, MG Brazil; 7 Department of Psychiatry, University of Toronto and Centre for Addiction and Mental Health–CAMH, Toronto, ON Canada; 8 Lozano Long School of Medicine, The Glenn Biggs Institute for Alzheimer’s and Neurodegenerative Diseases, University of Texas Health Science Center at San Antonio, San Antonio, TX USA; 9 Faculdade Santa Casa BH, Belo Horizonte, MG Brazil; 10 Departamento de Clínica Médica, Faculdade de Medicina, UFMG, Belo Horizonte, MG Brazil; 11 Departamento de Computação, Instituto de Ciências Exatas e Biológicas, Universidade Federal de Ouro Preto-UFOP, Ouro Preto, MG Brazil

**Keywords:** Mild traumatic brain injury, Concussion, Serum biomarkers, Random forest, SHAP, Machine-learning

## Abstract

The purpose of this study is to identify acute-phase serum biomarkers for mild traumatic brain injury (mTBI) within 24 h of trauma and evaluate their discriminatory value versus orthopedic trauma and healthy status using a leakage-safe machine-learning framework. We enrolled 60 patients with computed tomography (CT)-negative mTBI (World Health Organization [WHO] criteria; Glasgow Coma Scale [GCS] 13–15; age 18–59) within 24 h of injury, 17 orthopedic injury controls, and 24 healthy controls without mTBI in the prior 5 years. Fifty-nine serum biomarkers were quantified by Luminex®. The group comparisons used ANOVA or Kruskal–Wallis tests with corrected post hoc contrasts. Random Forest models were trained under leakage-safe nested cross-validation (outer 5-fold; inner 3-fold) with biomarker-only and biomarker-plus-demographics feature sets. SHAP values were computed on held-out folds and stability was assessed across resamples. Twenty-three biomarkers differed across groups after Bonferroni correction for post hoc comparisons (BDNF, EGF, Fracktalkine, G-CSF, GRO, IL15, IL-1α, IL-1β, IL-2, IL-3, IL-4, IL-5, IL-9, LIGHT, MMP-9, MPO, NCAM, NRG1-β1, S100B, TGF- α, VEGF A, sICAM-1). In nested cross-validation, three-class balanced accuracy ranged ~0.48–0.70, and mTBI one-vs-rest receiver operating characteristic area under the curve (ROC-AUC) frequently exceeded 0.85; Control vs mTBI classification achieved balanced accuracy ~0.70–0.90 with precision–recall area under the curve (PR-AUC) consistently >0.82. Sex and age added minimal incremental value. Stable SHAP contributors (top-5 in ≥4/5 folds) were LIGHT, IL-5, sCD40L, MMP-9, EGF, and fractalkine. Acute mTBI is associated with a reproducible serum signature spanning inflammatory, vascular, and growth-factor pathways.

**Supplementary Information:**

The online version contains supplementary material available at https://doi.org/10.1007/s12035-026-06130-1.

## Introduction

Traumatic brain injury (TBI) presents a major public health challenge due to its high incidence and socioeconomic burden. In 2016, approximately 2 million individuals in the United States had TBI, generating an estimated $40.6 billion in healthcare expenditure [1]. Beyond direct medical costs, work loss attributable to TBI-associated cognitive and physical impairments contributed to an additional $69 billion in economic losses, emphasizing the far-reaching societal consequences of this condition [2]. These numbers emphasize the critical need for effective preventive strategies and evidence-based management across all levels of TBI severity. The current research aims at defining blood-based biomarkers for mild TBI (mTBI), the most frequent presentation of TBI.

The diagnosis of mTBI and its related impact can be challenging due to its subtle clinical manifestations and the absence of definitive biomarkers [3]. For example, mTBI can cause persistent psychological distress despite its less overt clinical presentation compared to more severe forms of TBI [4]. Recent studies have indicated potential biomarkers of mTBI identified through imaging techniques, behavioral assessments, and protein metabolism approaches [5]. For instance, salivary biomarkers, including S100 calcium-binding protein B (S100B), neurofilament light polypeptide (NF-L), microRNAs, and extracellular vesicles, have demonstrated diagnostic potential for mTBI [6]. Sustained astroglial activation, as evidenced by increased total distribution volume of the astroglia marker 11C-SL25.1188 in the prefrontal cortex, thalamus, and hippocampus, has also been reported in individuals with mild to moderate TBI and persistent symptoms [7]. Importantly, there was a negative correlation between elevated 11C-SL25.1188 distribution volume and cognitive function [7]. Altered glial fibrillary acidic protein (GFAP) and ubiquitin C-terminal hydrolase L1 (UCH-L1) were useful for detecting brain lesions on computed tomography (CT) scans in mTBI, whereas biomarkers like S100B, NF-L, and total tau played a minimal role in predicting mTBI outcomes [8]. Conversely, Hier et al. [9] observed that biomarkers had limited utility in distinguishing mTBI from non-mTBI individuals or predicting mTBI recovery. Given the conflicting findings in the literature, the definition of a biomarker signature of mTBI is still elusive. Potential biomarkers linked to long-term neuropsychiatric sequelae still remains to be determined, which may have meaningful clinical impact.

This preliminary study utilized a machine-learning-driven approach to analyze serum-based biomarkers of acute mTBI within the first 24 h following mTBI. Our exploratory analysis aimed to identify specific biomarkers of mTBI that could lead to early and precise diagnosis. We expect that these biomarkers will guide future prognostic studies to refine treatment and rehabilitation.

## Methods

### Participants

We consecutively recruited 60 patients with mTBI, as defined by the World Health Organization (WHO) Task Force criteria [10], from the João XXIII Emergency Hospital in Belo Horizonte, Brazil, within 24 h of trauma. The initial diagnosis of mTBI was performed by a neurology team with trauma expertise. Patients were selected based on their medical records indicating mTBI diagnosis. Sociodemographic and clinical data, such as loss of consciousness (LOC), were obtained from medical records and interview.

Age of the patients ranged from 18 to 59 years and had a Glasgow Coma Scale (GCS) score of 13–15 upon admission. All patients underwent a CT scan; therefore, the exclusion criteria included any CT abnormalities, skull fractures, penetrating trauma, pregnancy, prior history of moderate/severe TBI or neurosurgery, autoimmune or neurodegenerative diseases, severe mental illnesses (i.e., schizophrenia, bipolar disorder or any active mental illness), use of medications other than nonsteroidal anti-inflammatory drugs (NSAIDs) and ketoprofen, and a current diagnosis of alcohol or substance use disorder (documented in medical records or self-reported during the interview).

For comparison, we recruited a healthy control group (*n* = 24) from the community with no history of TBI in the past 5 years, following the same exclusion criteria. This method was the only way to ensure the inclusion of patients without a history of trauma. We also recruited a ‘positive’ control (orthopedic) group (*n* = 17) consisting of individuals with arm or leg fractures who were also treated at the João XXIII Emergency Hospital. To keep consistency, the orthopedic control group underwent the same recruitment process as the mTBI group. Participants were selected based on their medical records and an interview was conducted that evaluated the fracture mechanism and injury kinematics. Through the assessment of clinical documentation and interviews, we ensured that no participant in the orthopedic control group was exposed to abrupt acceleration or deceleration forces capable of causing TBI.

Patients with mTBI and both control groups were comparable in terms of age, sex, and educational level.

The study was approved by the Universidade Federal de Minas Gerais and hospital ethics committee (CAAE 49623015.0.0000.5149).

### Blood Sample Processing

Peripheral blood was collected from mTBI patients and controls using vacuum tubes. The samples were centrifuged at 4 °C and 2500×*g* for 10 min to separate the serum, which was stored at –80 °C for subsequent analyses. Using the Luminex^®^ microsphere technique, we quantified various biomarkers including inflammatory, vascular, neuronal, and glial markers (see Supplementary Material, Table 1). For these assays, we used a customized panel for human magnetic cytokines/chemokines (Merck Millipore, Darmstadt, Germany; R&D Systems, Minneapolis, MN, USA) and neuroscience kits (Milliplex MAP Human Neuroscience Panel 1 Magnetic Bead Panel [kit lot 3007830], Milliplex MAP Human Neurodegenerative Disease Magnetic Bead Panel 3 [kit lot 3007831]). All protocols were carried out according to the manufacturers’ guidelines. Data were acquired using Luminex’s xMAP^®^ instruments (MAGPIX®, Merck Millipore) and processed using Exponent^®^ software (LUMINEX, Austin, TX, USA). Additional analyses were performed using MILLIPLEX^®^ Analyst 5.1 (Merck Millipore, Darmstadt, Germany). All results are reported in picograms per milliliter (pg/mL). The Luminex^®^ microsphere technique employed to investigate biomarkers levels provides quantitative values. All measures were performed on the same experiment using internal standards and all samples were analyzed in the same batch.

**Table 1:** Sociodemographic and clinical data

	mTBI	Orthopedic injury	Healthy control	*p-*value
Biological sex				
Male (n)	35 (58.3%)	15 (88.2%)	11 (45.8%)	0.021*
Female (n)	25 (41.7%)	2 (11.8%)	13 (54.2%)	
Age (Mean ± SD)	34.5 ± 10.3	31.7 ± 11.6	39.6 ± 11.6	0.055^#^
Ethnicity				
White	5 (8.3%)	4 (23.5%)	N/A	N/A
Brown	29 (48.3%)	8	N/A	
Black	25 (41.7%)	5	N/A	
Not Reported	1 (1.7%)	0 (0.0%)	24 (100%)	
TBI mechanism				
Fall From Own Height	17 (28.3%)	N/A	N/A	< 0.000*
Car Accident	7 (11.7%)			
Motorcycle Accident	8 (13.3%)			
Physical Assault without Object	3 (5.0%)			
Physical Assault with Object	2 (3.3%)			
Pedestrian Accident	3 (5.0%)			
Fall from Varying Height	14 (23.3%)			
Others	6 (10.0%)			
GCS (Mean ± SD)	14.7 ± 0.6 (*N*= 53)#	N/A	N/A	N/A
Loss of consciousness	63.2% (*N*=36 out of 57)#	N/A	N/A	N/A

Biological sex and TBI mechanism were evaluated using Chi-square tests (*). Age was evaluated using an ANOVA test (^#^) #. Data were partially missing, with GCS reported for 53 of 60 patients and loss of consciousness (LOC) recorded for 57 patients

*N/A* not applicable, *SD* standard deviation, *mTBI* mild traumatic brain injury, *GCS* Glasgow Coma Scale

### Statistical Analysis

#### Conventional Group Comparisons of Clinical and Biomarker Measures

At first, statistical analyses were conducted using SPSS (version 26.0). Descriptive data are presented as means, standard deviations, and frequencies. We used the Shapiro–Wilk test to assess the distribution of each variable. For variables following a normal distribution, an analysis of variance (ANOVA) test was used to compare differences among groups; for those not normally distributed, the Kruskal–Wallis test was employed. The Chi-square test (or Fisher’s exact test, if necessary) was used to compare categorical frequencies. When the Kruskal–Wallis or ANOVA results were significant, Tukey’s multiple comparisons test or Dunn’s method, respectively, was applied to determine which groups differed significantly.

#### Machine-Learning-Based Group Classification and Model Interpretation

We implemented a leakage-safe machine-learning pipeline to evaluate the discriminatory value of the serum biomarker panel (Supplementary Table S1) for distinguishing Healthy Control vs. mTBI vs. Orthopedic Injury groups using Python 3.10. All preprocessing operations were executed within the training partition of each outer fold only and the learned transformations were then applied to the corresponding held-out test partition to prevent leakage and optimistic bias. Preprocessing consisted of (i) quantile-based ‘winsorization’ (clipping each biomarker to the 1st–99th percentiles computed from the training fold), (ii) median imputation for missing values using training-fold medians, and (iii) robust scaling (median–interquartile range [IQR] normalization) fit on training data only; no biomarkers were excluded, and the number of ‘winsorized’ observations per biomarker was tracked for quality control. Model development and evaluation were performed under a nested cross-validation framework, with an outer five-fold StratifiedKFold for unbiased generalization estimates and an inner three-fold StratifiedKFold restricted to each outer training fold for hyperparameter selection via grid search.

Random Forest models were optimized over a prespecified grid (*n*_estimators {300, 500}; max_depth {None, 8, 12}; min_samples_leaf {1, 2, 4}; max_features = “sqrt”; bootstrap = True), using a fixed random_state = 42 to ensure determinism [11]. To quantify the incremental value of demographics, we evaluated two parallel feature configurations: biomarkers only (bio_only) and biomarkers plus age and sex (bio_demo). We trained (i) a three-class model (Control, mTBI, Orthopedic Injury) and (ii) pairwise binary models (Control vs. mTBI; Control vs. Orthopedic Injury). Model performance was summarized using balanced accuracy, macro-F1, receiver operating characteristic (ROC) area under the curve (AUC) (ROC-AUC) (one-vs-rest for three-class; standard AUC for binary), and precision–recall (PR) area under the curve (AUC) (PR-AUC) (one-vs-rest for three-class; binary PR-AUC for pairwise), with confusion matrices and ROC/PR curves reported as complementary diagnostics under class imbalance; uncertainty was quantified with fold-wise variability and bootstrap 95% confidence intervals (*n* = 1000).

For interpretability, we applied SHapley Additive exPlanations (SHAP) to Random Forest predictions using a leakage-safe approach in which SHAP values were computed exclusively on the held-out outer test folds [12]. We summarized per-fold mean absolute SHAP values and quantified robustness via (i) fold-wise rank variability heatmaps and (ii) Top-10 recurrence (“stability”) analyses across outer folds, highlighting biomarkers whose importance persisted under resampling.

Machine-learning analyses were conducted using scikit-learn (≥ 1.4) and SHAP (≥ 0.45), with fixed seeds and fold-contained preprocessing [12, 13]. Additional details regarding this machine-learning approach are provided in the Supplementary Material.

## Results

### Demographic Analysis

Table 1 summarizes demographic and clinical data for the three groups. There were more men in the mTBI and orthopedic groups compared to controls (*p*=0.02). Age differences were not significant (*p*=0.06). A fall from one’s own height (*n*=17) was the most frequent TBI mechanism, followed by falls from varying heights (*n*=14). No post-hoc calibration was required for demographic analysis.

### Group Comparisons of Serum Biomarkers

Descriptive statistics for all biomarkers stratified by group are presented in Supplementary Table S2, with normality assessments in Supplementary Table S3**.** The overall results of omnibus comparisons (ANOVA or Kruskal–Wallis, depending on distribution) are summarized in Supplementary Table 4, and Bonferroni-adjusted Dunn post hoc pairwise contrasts for significant findings are reported in Supplementary Table S5.

Among the 59 biomarkers examined, 25 showed significant between-group differences on Kruskal–Wallis testing (*p* < 0.05).

Cytokine analyses revealed distinct group-specific patterns across multiple interleukins. Interleukin (IL)− 1*α* (H(2) = 13.283, *p* = 0.001), IL-1*β* (H(2) = 17.278, *p* < 0.001), IL-2 (H(2) = 11.115, *p* = 0.004), IL-3 (H(2) = 10.662, *p* = 0.005), IL-4 (H(2) = 10.647, *p* = 0.005), IL-5 (H(2) = 20.793, *p* < 0.001), IL-9 (H(2) = 11.427, *p* = 0.003), and IL-15 (H(2) = 8.055, *p* = 0.018) all differed significantly among groups. C-X-C motif chemokine ligand 1 (GRO-KC) also differed across groups (H(2) = 12.109, *p* = 0.002) and was higher in the mTBI group than in healthy controls (Dunn–Bonferroni *p*_adj_ = 0.002). Beyond these cytokine differences, fractalkine also varied by group (H(2) = 7.992, *p* = 0.018), with higher concentrations in mTBI than in the orthopedic injury group. Post hoc Dunn–Bonferroni tests showed that IL-4 was lower in the mTBI group than in healthy controls (*p*_adj_ = 0.015), IL-5 was lower in mTBI than in the orthopedic injury group (*p*a_dj_ < 0.001), IL-1*β* was lower in mTBI than in both healthy controls (*p*_adj_ = 0.040) and orthopedic injury (*p*_adj_ < 0.001), and IL-3 (*p*_adj_ = 0.004) and IL-9 (*p*_adj_ = 0.002) were also lower in mTBI compared with orthopedic injury.

Markers related to immune activation and cell adhesion also showed group differences. tumor necrosis factor superfamily member 14 (LIGHT) (H(2)=20.024, *p* < 0.001) and myeloperoxidase (MPO) (H(2)=14.415, *p* = 0.001) were elevated in mTBI compared with healthy participants (*p*_adj_ < 0.001 for both biomarkers). Post hoc Dunn–Bonferroni tests showed that matrix metalloproteinase−9 (MMP-9) differed between healthy controls and the mTBI group (*p*_adj_ < 0.001), with higher concentrations in mTBI, while soluble intercellular adhesion molecule−1 (sICAM-1) differed between orthopedic injury and mTBI (*p*_adj_ = 0.016), with higher concentrations in mTBI; additionally, neural cell adhesion molecule (NCAM) was reduced in orthopedic injury relative to healthy controls (*p*_adj_ = 0.022).

Growth and neurotrophic factors also differed across clinical groups. Brain-derived neurotrophic factor (BDNF) (H(2) = 8.711, *p* = 0.013) was lower in the orthopedic injury group than in healthy controls (*p*_adj_ = 0.010), whereas granulocyte colony-stimulating factor (G-CSF) (H(2) = 10.122, *p* = 0.006) was higher in orthopedic injury than in healthy controls (*p*_adj_ = 0.005). Neuregulin-1 *β*1 (NRG1-*β*1) (H(2) = 10.383, *p* = 0.006) differed between orthopedic injury and mTBI (*p*_adj_ = 0.050), with higher concentrations in mTBI. Epidermal growth factor (EGF) also differed across groups (H(2) = 11.719, *p* = 0.003), with lower concentrations in mTBI than in the orthopedic injury group (Dunn–Bonferroni *p*_adj_ = 0.006). Transforming growth factor- *α* (TGF-α) (H(2) = 11.456, *p* = 0.003) was higher in mTBI than in healthy controls (*p*_adj_ = 0.002), and vascular endothelial growth factor-A (VEGF-A) (H(2) = 9.415, *p* = 0.009) also differed between healthy controls and mTBI (*p*_adj_ = 0.033), with higher concentrations in mTBI.

Finally, several molecules reached significance at the omnibus level but did not retain pairwise significance after Bonferroni correction, including lipocalin-2 (H(2)=6.139, *p* = 0.046), macrophage migration inhibitory factor (MIF) (H(2)=7.188, *p* = 0.027), and platelet-derived growth factor-AA (PDGF-AA) (H(2)=6.153, *p* = 0.046). For transparency, all biomarkers (including those with nonsignificant omnibus results) are presented in the supplementary material (Tables S1–S5).

### Machine Learning Analyses

Machine-learning analyses demonstrated consistent discriminative ability across both the three-class and pairwise classification tasks. Under the fully leakage-safe nested cross-validation design, balanced accuracy for the three-class model ranged approximately from 0.48 to 0.70 across outer folds, with ROC–AUC values frequently exceeding 0.85 for the TBI class. Across outer folds, models trained with biomarkers alone (bio_only) performed similarly to those incorporating age and sex (bio_demo), indicating that the biological signal dominated the predictive structure.

For the binary comparisons, discrimination between Control vs. TBI (0×1) was high, with balanced accuracy typically between 0.70 and 0.90, and PR-AUC consistently above 0.82, reflecting the robustness of the biomarker signature associated with acute neurotrauma. In contrast, the Control vs. Orthopedic Injury (0×2) task showed greater variability (including balanced accuracies ranging from approximately 0.40 to 0.80) consistent with the biological proximity of these two non-neurological cohorts.

Across all tasks, ROC and PR curves (Supplementary Figs. S3, S4, S6–S7, S9–S10) showed stable, overlapping performance between the bio_demo and bio_only scenarios, further confirming that demographics make only a modest incremental contribution relative to the biomarker panel.

Finally, SHAP-based interpretability analyses (Supplementary Figs. S11–S24) revealed a subset of cytokines and inflammatory markers that consistently contributed to the model’s predictions across resampling folds, with high stability in Top-10 importance rankings. This reproducibility reinforces the biological relevance of the identified biomarkers and supports their potential utility in distinguishing TBI from other clinical groups. A full breakdown of performance metrics (fold-wise and summarized confidence intervals), confusion matrices, ROC/PR curves, best Random Forest hyperparameters, and detailed SHAP stability and rank analyses are reported in Supplementary Tables S6–S12 and Supplementary Figs. S2–S24.

#### mTBI vs Healthy Controls

**Fig. 1:**
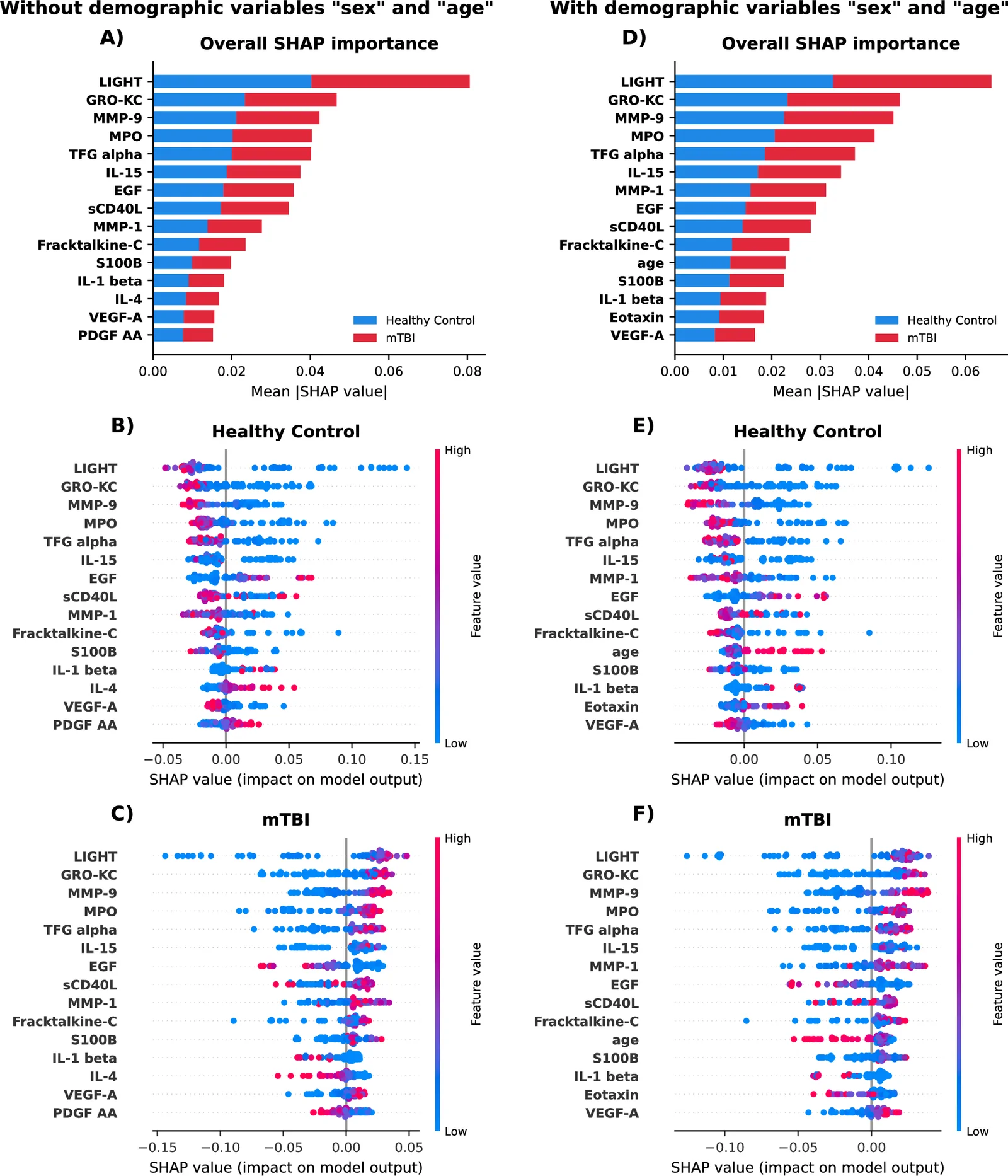
SHAP analysis of random forest models comparing mTBI and healthy controls, with and without demographic variables. **A** Global feature importance for the model including only serum biomarkers, expressed as mean absolute SHAP value for each variable, with blue bars for healthy controls and red bars for mTBI. **B** SHAP beeswarm plot for the probability of belonging to the healthy control group in the biomarker-only model; each point represents one participant, the horizontal axis shows the SHAP value (impact on model output), and the color scale denotes the underlying feature value from low (blue) to high (red). **C** Corresponding SHAP beeswarm plot for the probability of belonging to the mTBI group in the biomarker-only model. **D** Global feature importance for the model that additionally includes the demographic variables age and sex, again summarized as mean absolute SHAP values and separated by group. **E** SHAP beeswarm plot for the healthy control group in the biomarker-plus-demographics model. **F** SHAP beeswarm plot for the mTBI group in the biomarker-plus-demographics model. In all subfigures, variables are ordered from top to bottom by decreasing mean absolute SHAP value, therefore features at the top exert the greatest influence on model predictions

**Fig. 2:**
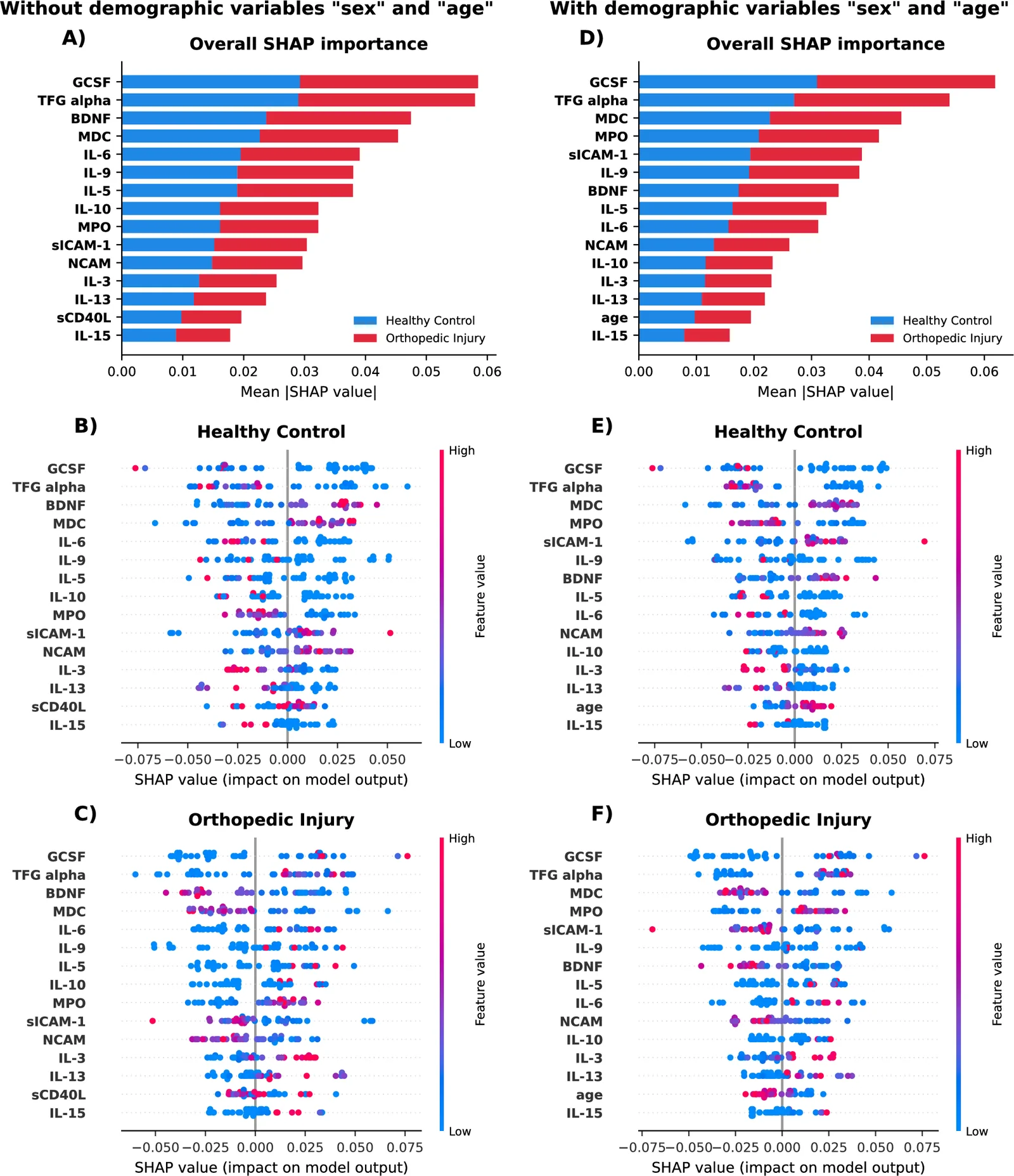
SHAP analysis of random forest models comparing healthy controls and the orthopedic injury group, with and without demographic variables.** A** Global feature importance for the model including only serum biomarkers, expressed as mean absolute SHAP value for each variable, with blue bars for healthy controls and red bars for orthopedic injury. **B** SHAP beeswarm plot for the probability of belonging to the healthy control group in the biomarker-only model; each point represents one participant, the horizontal axis shows the SHAP value (impact on model output), and the color scale denotes the underlying feature value from low (blue) to high (red). **C** Corresponding SHAP beeswarm plot for the probability of belonging to the orthopedic injury group in the biomarker-only model. **D** Global feature importance for the model that additionally includes the demographic variables age and sex, again summarized as mean absolute SHAP values and separated by group. **E** SHAP beeswarm plot for the healthy control group in the biomarker-plus-demographics model. **F** SHAP beeswarm plot for the orthopedic injury group in the biomarker-plus-demographics model. In all subfigures, variables are ordered from top to bottom by decreasing mean absolute SHAP value, therefore features at the top contribute most strongly to model predictions

Figure 1 summarizes the SHAP-based interpretation of the random forest models contrasting mTBI with healthy controls. In the biomarker-only model, global feature importance (Fig. 1A) shows that LIGHT, GRO-KC, MMP-9, and MPO have the largest mean absolute SHAP values for both classes, followed by TGF-α, IL-15, EGF, soluble CD40 ligand (sCD40L), matrix metalloproteinase−9 (MMP-1), fractalkine, and S100B indicating that this set of inflammatory and vascular markers contributes most to class discrimination. The corresponding beeswarm plot for the healthy control class (Fig. 1B) reveals that low values of LIGHT, GRO-KC, MMP-9, and MPO are associated with positive SHAP values and therefore shift predictions toward the healthy group, whereas higher values of these markers reduce the probability of being classified as healthy. The beeswarm plot for the mTBI class (Fig. 2C) displays the reverse pattern: higher concentrations of LIGHT, GRO-KC, MMP-9, and MPO increase the likelihood of mTBI classification, while lower levels favor the healthy class.

When age and sex are added to the model, global importance remains largely unchanged (Fig. 1D): LIGHT, GRO-KC, MMP-9, and MPO continue to dominate the ranking, while age appears in the middle of the spectrum and sex remains among the least influential features, suggesting that most predictive information derives from the biomarker panel rather than demographics. The beeswarm plots for the extended model (Fig. 1E and F) reproduce the same directional effects observed in the biomarker-only model for the healthy control and mTBI classes, respectively. Inclusion of age and sex does not materially alter the SHAP patterns, indicating that the discriminative signal for mTBI versus healthy controls is largely captured by the biomarker profile.

#### Orthopedic Injury vs Healthy Control

In the comparison between healthy controls and the orthopedic injury group (Fig. 2), SHAP analysis showed that discrimination between groups was driven mainly by a limited set of inflammatory markers. In the model including biomarkers only, global feature importance (Fig. 2A) indicated that G-CSF, TFG-α, BDNF, and MDC, contributed most strongly to classification, followed by IL-6, IL-9, IL-5, and IL-10. The beeswarm plots clarify the direction of these effects: for the healthy control class (Fig. 2B), lower values of G-CSF, TFG-α, and higher concentrations of BDNF, MDC were associated with positive SHAP values, shifting predictions toward the healthy group. For the orthopedic injury class (Fig. 2C), the pattern was the opposite.

When age and sex were added to the model (Fig. 2D–F), the SHAP profiles changed little. G-CSF, TFG-α, and MDC remained the most influential variables in the global importance plot, but now with MPO at the top four most influential variables (Fig. 2D), while age and sex appeared near the bottom of the ranking. The beeswarm plots for the healthy control (Fig. 2E) and orthopedic injury (Fig. 2F) classes reproduced similar directional patterns observed in the biomarker-only model, indicating that demographic variables added minimal predictive information beyond the biomarker panel.

#### mTBI vs Orthopedic Injury vs Healthy Controls

**Fig. 3:**
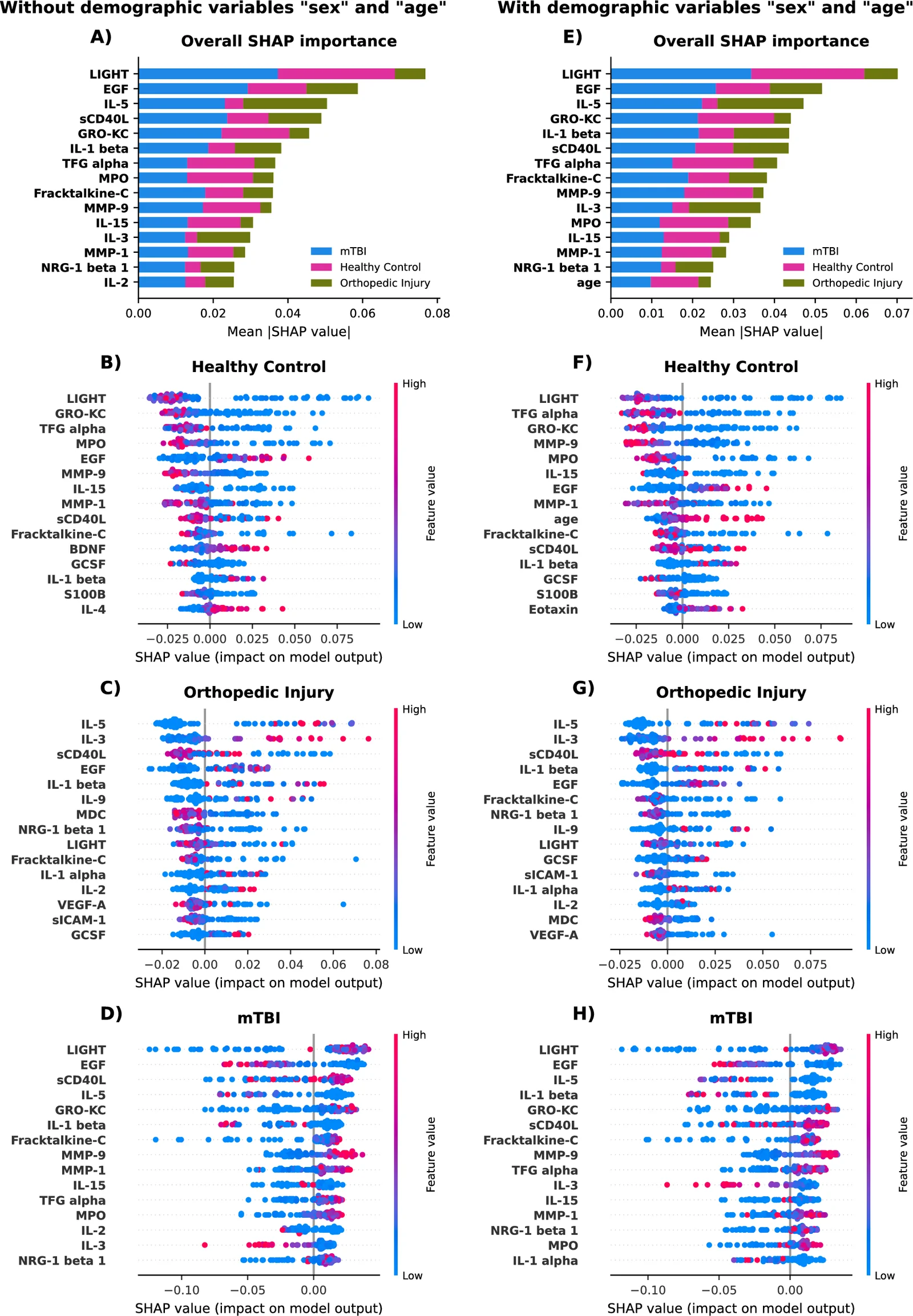
SHAP analysis of three-class random forest models comparing mTBI, healthy controls, and the orthopedic injury group, with and without demographic variables.** A** Global feature importance for the model using biomarker data alone, expressed as mean absolute SHAP value for each variable, with stacked bars indicating contributions to predictions for mTBI (blue), healthy controls (pink), and orthopedic injury (green). **B** SHAP beeswarm plot for the probability of belonging to the healthy control group in the biomarker-only model; each point represents one participant, the horizontal axis shows the SHAP value (impact on model output), and the color scale denotes the corresponding feature value from low (blue) to high (red). **C** SHAP beeswarm plot for the orthopedic injury group in the biomarker-only model. **D** SHAP beeswarm plot for the mTBI group in the biomarker-only model. **E** Global feature importance for the model that includes biomarkers plus the demographic variables age and sex, summarized as mean absolute SHAP values and partitioned by class. **F** SHAP beeswarm plot for the healthy control group in the biomarkers-plus-demographics model. **G** SHAP beeswarm plot for the orthopedic injury group in the biomarkers-plus-demographics model. **H** SHAP beeswarm plot for the mTBI group in the biomarkers-plus-demographics model. In all subfigures, variables are ordered from top to bottom by decreasing mean absolute SHAP value, therefore features appearing higher in the plots contribute more strongly to class predictions

Figure 3 summarizes the SHAP-based interpretation of the three-class random forest models contrasting mTBI, healthy controls, and the orthopedic injury group. In the model using biomarker data alone (Fig. 3A), LIGHT and EGF showed the highest mean absolute SHAP values across classes, followed by IL-5, sCD40L, GRO-KC, IL-1β, TFG-α, MPO, Fractalkine, and MMP-9, indicating that these inflammatory and growth-factor markers concentrated most of the discriminative signal. The beeswarm plot for the healthy control class (Fig. 3B) shows that lower values of LIGHT, GRO-KC, TFG-α, and MPO were associated with positive SHAP values and therefore pushed predictions toward the healthy group, whereas higher concentrations of these markers reduced the probability of being classified as healthy. For the orthopedic injury group (Fig. 3C), higher levels of IL-5, IL-3, EGF, IL-1β and lower levels of sCD40L shifted predictions toward orthopedic injury, consistent with a systemic trauma-related inflammatory profile. In contrast, the mTBI class (Fig. 3D) was characterized by higher concentrations of LIGHT, sCD40L, GRO-KC, Fractalkine, and MMP-9and lower concentrations of EGF, IL-5, IL-1β.

Adding age and sex to the model did not substantially alter the structure of the SHAP profiles. In the biomarkers-plus-demographics model (Fig. 3E), LIGHT, EGF, and IL-5 remained the most influential features; GRO-KC rose to fourth, whereas sCD40L fell to sixth most influential feature. IL-1β, fractalkine, MMP-9, and TFGα continued to contribute prominently, while age appeared near the bottom of the importance ranking. The corresponding beeswarm plots for healthy controls (Fig. 3F), orthopedic injury (Fig. 3G), and mTBI (Fig. 3H) reproduced similar directional patterns observed in the biomarker-only model. Separation between mTBI, healthy controls, and orthopedic injuries is driven predominantly by a shared set of inflammatory and vascular biomarkers, with demographic variables providing limited additional predictive information.

## Discussion

Biomarkers provide insights into the pathophysiology of diseases and injuries and have potential as diagnostic, prognostic, and theranostic tools [14]. To minimize over-interpretation of unstable findings, we restricted detailed biological interpretation to biomarkers with high stability, defined as appearing in the top five SHAP-ranked features in at least four of five cross-validation folds (stability score ≥ 4/5). Under this criterion, LIGHT, IL-5, sCD40L, MMP-9, EGF, and fractalkine emerged as the most reproducible contributors to class separation. To the best of our knowledge, this is the first study to apply random forest models combined with SHAP interpretability to investigate mTBI-specific biomarkers.

The three-group design, which contrasted mTBI not only with healthy participants but also with an orthopedic trauma group, allowed us to test whether the most stable biomarkers reflected head injury or general trauma. Predictions for the mTBI class were more strongly influenced by LIGHT, EGF, and MMP-9, which showed high SHAP stability for mTBI. Taken together, these patterns support a two-component framework in which acute mTBI comprises a trauma-related systemic inflammatory module, indexed primarily by IL-5, EGF, sCD40L, and fractalkine, superimposed on a head-injury–weighted neurovascular–microglial signature centered on LIGHT, MMP-9, sCD40L, EGF, and fractalkine.

LIGHT belongs to the tumor necrosis factor (TNF) superfamily and signals through herpesvirus entry mediator (HVEM) and lymphotoxin-β receptor (LTβR), promoting T cell activation, microglial priming, and endothelial activation. LIGHT also induces a proinflammatory endothelial phenotype, including upregulation of adhesion molecules such as intercellular adhesion molecule 1 (ICAM-1) and vascular cell adhesion molecule 1 (VCAM-1) and production of chemokines and prostanoids in human vascular endothelial cells, a pattern that would be expected to influence vascular barrier regulation and could extend to the blood–brain barrier interface [15, 16]. Additionally, single-concussion athletes showed significantly lower serum LIGHT levels than healthy controls [17]. They also showed significantly lower LIGHT levels when samples were collected more than 1 week after injury [17]. However, repetitively concussed athletes did not show a significant decrease in LIGHT relative to healthy controls [17]. In our data, LIGHT consistently differentiated mTBI from both healthy and orthopedic groups in multigroup models, which suggests that acute mTBI may preferentially engage this TNF-superfamily axis compared with systemic trauma without head injury.

MMP-9 and sCD40L highlighted a vascular and platelet–endothelial component of the acute mTBI response. MMP-9 degrades type IV collagen and tight junction proteins in the vascular basement membrane [18]. In human cohorts with moderate-to-severe TBI, cerebrospinal fluid (CSF) MMP-9 rises early, can remain elevated for up to 72 h, is higher in severe than in moderate injury, and higher admission CSF MMP-9 predicts poorer 6-month functional outcome [19, 20]. In the same reports, plasma MMP-9 was also higher than in controls, but it did not show clear correlations with blood–brain barrier permeability or intracranial pressure in these small series [19, 20]. In adults evaluated within 24 h of concussion versus uninjured and orthopedic-injury controls, plasma MMP-9 was elevated within 8 h post-injury, supporting its potential utility for early concussion discrimination even in the context of concomitant orthopedic trauma [21]. These MMP-9 findings support early cerebrovascular matrix remodeling after mTBI and motivate consideration of complementary platelet-derived signals such as sCD40L.

Activated platelets express CD40L that they store in α-granules and cleavage releases soluble sCD40L into the circulation [22]. sCD40L can then engage CD40 on monocytes and endothelial cells, which promotes proinflammatory signaling, induces nuclear factor kappa B (NF-κB)–dependent cytokines and tissue factor, and supports procoagulant activity and vascular thrombosis [22]. In a multicenter cohort of 100 patients with isolated severe TBI, higher admission serum sCD40L concentrations correlated with higher Acute Physiology and Chronic Health Evaluation II (APACHE II) scores and lower GCS, and independently predicted 30-day mortality supporting its role as a prognostic severity marker [22]. In our models, sCD40L achieved high SHAP stability for mTBI and orthopedic injury classification and contributed to the three-way separation of mTBI, orthopedic trauma, and healthy status. In a pilot cohort (21 mTBI within 1 month vs 19 controls), machine-learning analyses linked impulsivity to a blood biomarker signature that included serum sCD40L, suggesting relevance to post-mTBI behavioral phenotype [23]. Together with MMP-9, this pattern supports the concept that acute mTBI involves early platelet activation and matrix remodeling at the cerebrovascular interface, even when standard CT imaging is normal.

IL-5 and EGF pointed to systemic inflammatory activity and early reparative responses. IL-5 is classically associated with T helper 2 (Th2) responses and eosinophil differentiation [24]. It also regulates B-cell function and contributes to cytokine networks that promote brain repair and limit inflammation [25]. Data on IL-5 in TBI remain sparse, but in a four-group cohort of younger and older adults with mTBI and age-/sex-matched controls, Thompson et al. found that plasma IL-5 increased within 24 h after mTBI and was still higher at 6 months in both younger and older mTBI participants, based on comparisons that used the younger control group as the reference [24]. In our analysis, lower IL-5 concentration contributed to class separation in the three-way model with high SHAP stability.

EGF, a mitogen for neural stem and progenitor cells, enhances TBI-induced proliferation in the subventricular zone and dentate gyrus, promotes differentiation of newly generated cells toward an astroglial phenotype, and exerts a neuroprotective effect by reducing neuronal loss in hippocampal CA3 and hilus with associated improvement in Morris water maze performance in rats [26]. In 140 sport-related concussion athletes with plasma collected within 48 h, EGF was lower in the prolonged-recovery group (≥ 14 days to asymptomatic) vs the faster-recovery group (< 14 days), linking early EGF signal to clinically meaningful recovery duration/return-to-sport stratification [27].In this context, the stable contribution of EGF to mTBI classification in our model is more consistent with early activation of neurotrophic and angiogenic repair programs than with a marker of direct tissue damage**.** Longitudinal work that tracks EGF together with structural and functional outcomes is needed to determine whether higher acute EGF levels predict better recovery or maladaptive gliosis.

Fractalkine and its C-X3-C motif chemokine receptor 1(CX3CR1) regulate neuron–microglia communication. In adult mice subjected to mild controlled cortical impact, deletion of CX3CR1 reduced early neuromotor impairment and neuronal loss but led to greater delayed neuron death and worse spatial learning at 30 days, indicating that CX3CR1 signaling has opposite effects in the acute and chronic phases after mTBI [28]. In a rugby-sport-related mTBI cohort, serum fractalkine was significantly upregulated in the early post-injury window (< 1 week) compared with healthy controls. By contrast, fractalkine was not significantly different at later timepoints (> 1 week). Together, this pattern suggests a transient, time-dependent fractalkine response that may index early inflammatory signaling after mTBI [17]. In a cohort of 313 participants (younger and older adults with mTBI plus age-/sex-matched controls), plasma fractalkine was higher in older adults with mTBI at day 0 [24]. Fractalkine showed higher levels in older controls than in younger mTBI participants [24]. Fractalkine remained significantly elevated at 1 month, suggesting an age-sensitive and relatively persistent fractalkine signal after mTBI [24]. Our models ranked fractalkine among the more stable contributors for several classes in three-class comparisons. Although more research is needed, this finding suggests that individual variation in neuron–microglia cross-talk may already be detectable in peripheral blood within 24 h of mTBI.

At a broader level, these patterns align with current views that mTBI biomarkers fall into several mechanistic clusters: neuronal and glial structural proteins such as GFAP and neurofilament light, systemic and central inflammatory mediators, and markers of vascular injury and coagulation [5, 8, 9]. Our exploratory analysis complements this growing knowledge by emphasizing a multi-marker inflammatory and vascular signature in the very acute phase. The reviews and meta-analyses indicate that inflammatory cytokines such as IL-6, IL-1 receptor antagonist, and CCL2 rise within hours after mTBI and may contribute to symptom persistence, although effect sizes are variable and assays are not yet standardized [29, 30]. Our findings identify LIGHT, MMP-9, sCD40L, EGF, and fractalkine as additional candidate markers, although they were derived from a hypothesis-generating machine-learning approach and require independent replication.

Several limitations temper the interpretation of these findings. The study used a modest, single-center sample and a cross-sectional design. As a result, the estimated effect sizes and SHAP importance rankings may be unstable in other populations, and we could not examine trajectories of biomarker change or relate acute levels to cognitive or neuropsychiatric outcomes. Sampling occurred within a 24-h window after trauma, but the exact number of hours since injury and the timing of analgesic administration were not available. Without precise timing information or stratification by different timepoints, temporal dynamics and pharmacologic effects of non-steroidal anti-inflammatory drugs and ketoprofen may confound the observed associations. The groups also differed in sex distribution, and although age and sex were included as covariates in adjusted models, the sample size precluded sex-stratified analyses. This limitation mirrors a broader gap in the biomarker literature, where age- and sex-related differences are rarely characterized and future work has been explicitly called for to address this issue [8]. In the orthopedic injury group, TBI absence was based on routine clinical documentation and patient report, consistent with usual emergency department practice. While we cannot fully exclude minor misclassification (due to acceleration/deacceleration forces), any undetected head trauma is likely to be limited and non-differential, and would be expected to bias group differences, if anything, toward the null. Finally, the machine-learning models were trained and evaluated within a single dataset. Cross-validation and the SHAP stability filter reduce overfitting but do not substitute for external validation in independent cohorts.

Despite the study’s limitations, several strengths support the relevance of our findings. We addressed a clinically practical question in an acute emergency department cohort. We also included an orthopedic injury group as a positive control. This design helps separate systemic trauma effects from brain-relevant patterns. We used a multimarker panel rather than a single-analyte approach. That choice better reflects the complex biology of mTBI. We paired this panel with an interpretable machine-learning framework using Random Forests and SHAP. This strategy enabled the identification of early serum candidates, including markers that are not routinely emphasized in standard TBI panels. The analytic workflow was fully leakage-safe. Outlier handling, winsorization, imputation, and scaling were performed only within training folds under nested cross-validation. This prevents information transfer from test data and supports unbiased estimates of generalization. Nested cross-validation also improves reliability in small, heterogeneous biomedical datasets. Random Forests were selected because they are robust in small samples. They also capture nonlinear and interaction effects that are plausible in neurotrauma. Such patterns can be non-monotonic and context dependent. Linear baselines such as multinomial logistic regression are transparent. Their linearity assumptions, however, can limit discovery unless extensive feature engineering is imposed. Tree-based ensembles accommodate heterogeneous feature scales and mixed data types with minimal manual specification. They also integrate naturally with SHAP for coherent feature-attribution analyses. Finally, fold-aggregated SHAP and Top-10 stability analyses strengthened interpretability. They helped distinguish consistently informative biomarkers from those with fold-dependent importance. Larger cohorts will be required to refine biomarker discrimination. Future work may also evaluate complementary approaches, including gradient boosting, multimodal models integrating imaging and clinical history, and longitudinal trajectories to characterize injury and recovery dynamics.

## Conclusion

In conclusion, we demonstrated via this exploratory analysis that acute mTBI is associated with a coordinated pattern of inflammatory and vascular biomarkers in peripheral blood. A small subset of markers with high SHAP stability, including LIGHT, MMP-9, sCD40L, IL-5, EGF, and fractalkine consistently contributed to the discrimination among mTBI, orthopedic trauma, and healthy controls. These molecules span neutrophil chemotaxis, oxidative burst, matrix remodeling, platelet–endothelial activation, growth factor signaling, and neuron–microglia communication, which supports a model of mTBI as a neuroimmune and neurovascular event rather than a purely structural brain insult. Given the exploratory design, modest sample size, and limited control over sampling time and medication exposure, our findings should be viewed as hypothesis generating. Larger, longitudinal, multi-center studies that integrate these candidates with established neuronal and glial biomarkers and with detailed clinical phenotyping will be necessary to determine whether any of these markers, alone or in combination, can support diagnosis, risk stratification, or treatment targeting in patients with mTBI. Additionally, future work should test whether biomarkers levels track lesion burden or predict persistence of post-concussive symptoms.

## Supplementary Information

Below is the link to the electronic supplementary material.

Supplementary file1 (PDF 4083 KB)

## Data Availability

No datasets were generated or analyzed during the current study.
