# Supplementary material for: Determining Acute-Phase Biomarkers for Mild Traumatic Brain Injury Through Exploratory Data Analysis: A Preliminary Report
Source: Mol Neurobiol. 2026 Sep 25;63(1):923. doi: 10.1007/s12035-026-06130-1 (PMC13614984; doi:10.1007/s12035-026-06130-1)
Supplement: Supplementary file 1 [file 12035_2026_6130_MOESM1_ESM.pdf]

APPENDIX

SUPPLEMENTARY MATERIAL

Supplementary Table 1 S1. Tested biomarkers and demographic variables

Demographic variables	Biomarkers
sex, age	EGF, FGF, Eotaxin, TFG-α, GCSF, GM CSF, Fractalkine, IFN α2, IFN-γ, GRO-KC, IL-10, MCP-3, IL-12 p40, MDC, IL-12 p70, IL-13, IL-15, sCD40L, IL-17A, IL-1α, IL-9, IL-1β, IL-2, IL-3, IL-4, IL-5, IL-6, IL-7, IL-8_CXCL8, IP-10, MCP-1, MIP-1α, MIP-1β, TNF-α, VEGF-A, BDNF, Catepsin D, sICAM-1, MPO, PDGF AA, RANTES, NCAM, PDGF-AB BB, sVCAM-1, PAI-1, MMP-9, Fibroblast, Neuropilin-1, Lipocalin-2, VEGF, MMP-1, MIF, S100B, LIGHT/TNFSF14, APP, RAGE, Enolase NSE, NRG1- β1, PARK5-UCHL1.

Supplementary Table 2 S2. Descriptive statistics of biomarker concentrations (pg/mL) by group.

Biomarker	Group	Mean (pg/mL)	SE (Mean)	CI Lower	CI Upper	Median (pg/mL)	Std. Deviation	Interquartile Range	Skewness	SE (Skewness)	Kurtosis	SE (Kurtosis)
APP	Healthy Control	23432.5417	2095.06355	19098.5725	27766.5108	23230.5000	10263.67333	10996.50	.866	.472	1.672	.918
	mTBI	23689.4000	1780.44073	20126.7463	27252.0537	21208.0000	13791.23459	18949.50	.708	.309	.373	.608
	Orthopedic Injury	23134.1765	2309.95984	18237.2804	28031.0726	21388.0000	9524.20839	15306.00	.595	.550	-.373	1.063
BDNF	Healthy Control	79991.0833	8476.80839	62455.4692	97526.6975	73529.0000	41527.71039	54995.00	1.062	.472	.527	.918
	mTBI	63927.0708	4575.60693	54771.3025	73082.8392	56699.0000	35442.49889	33428.50	1.718	.309	4.508	.608
	Orthopedic Injury	46797.2353	4299.87398	37681.9097	55912.5609	44194.0000	17728.83459	24945.00	-.695	.550	.452	1.063
Catepsin D	Healthy Control	369050.8750	30993.41682	304936.1074	433165.6426	340784.0000	151836.11318	176253.50	1.945	.472	5.756	.918
	mTBI	414939.2265	38434.07009	338032.8299	491845.6231	333481.0000	297709.02676	165936.75	2.620	.309	7.552	.608
	Orthopedic Injury	331889.9412	43056.97560	240613.2304	423166.6519	312500.0000	177528.45833	142227.50	1.457	.550	4.095	1.063
EGF	Healthy Control	160.4067	32.68397	92.7947	228.0186	115.2100	160.11811	141.85	1.594	.472	1.683	.918
	mTBI	101.5573	16.30374	68.9336	134.1810	58.8000	126.28824	59.60	2.687	.309	7.217	.608
	Orthopedic Injury	174.5806	33.03331	104.5531	244.6081	129.0000	136.19981	130.46	1.512	.550	1.706	1.063
Enolase NSE	Healthy Control	4700.9988	735.62890	3179.2344	6222.7631	4040.0000	3603.83089	4881.25	.586	.472	-.483	.918
	mTBI	4953.4328	455.35087	4042.2778	5864.5878	4217.5000	3527.13267	3727.75	1.405	.309	2.429	.608
	Orthopedic Injury	3646.2724	391.67702	2815.9542	4476.5906	3043.0000	1614.92574	2529.50	.248	.550	-.368	1.063
Eotaxin	Healthy Control	77.9446	12.11532	52.8821	103.0070	70.6900	59.35273	65.39	1.782	.472	4.928	.918
	mTBI	65.6003	8.03040	49.5315	81.6691	50.2400	62.20325	33.07	2.985	.309	10.609	.608
	Orthopedic Injury	155.9371	69.04375	9.5708	302.3033	54.1800	284.67469	103.95	3.875	.550	15.512	1.063
FGF	Healthy Control	129.3863	47.25881	31.6240	227.1485	35.8200	231.51993	104.30	3.122	.472	10.299	.918
	mTBI	112.0567	55.53015	.9411	223.1722	34.0200	430.13472	35.03	7.134	.309	52.977	.608
	Orthopedic Injury	222.1929	109.61541	-10.1813	454.5672	41.6000	451.95591	74.15	2.819	.550	8.300	1.063
Fibroblast	Healthy Control	35770.5000	1765.98771	32117.2761	39423.7239	38238.5000	8651.53755	10734.25	-1.028	.472	1.514	.918

	mTBI	35620.9000	996.26687	33627.3746	37614.4254	36375.0000	7717.04999	11456.50	-.553	.309	-.338	.608
	Orthopedic Injury	35375.5882	2007.84180	31119.1538	39632.0227	36781.0000	8278.54383	8750.00	-1.185	.550	1.411	1.063
Fracktalkine	Healthy Control	315.2621	60.53378	190.0384	440.4858	291.4850	296.55375	350.93	2.302	.472	7.740	.918
	mTBI	476.4485	58.69117	359.0077	593.8893	261.7100	454.61984	603.86	1.857	.309	4.925	.608
	Orthopedic Injury	271.7506	80.22934	101.6720	441.8292	141.5300	330.79405	307.68	2.857	.550	9.690	1.063
GCSF	Healthy Control	27.9917	3.97774	19.7631	36.2202	23.5150	19.48687	14.66	2.236	.472	6.531	.918
	mTBI	168.9308	74.51581	19.8250	318.0366	32.3500	577.19702	43.23	5.433	.309	30.865	.608
	Orthopedic Injury	200.5247	91.86717	5.7750	395.2744	56.2600	378.77804	105.84	2.939	.550	9.165	1.063
GM CSF	Healthy Control	8.2004	5.74404	-3.6820	20.0829	.6800	28.13995	2.61	4.760	.472	23.017	.918
	mTBI	6.4238	2.23004	1.9615	10.8861	1.6300	17.27379	3.33	4.237	.309	18.625	.608
	Orthopedic Injury	45.2300	31.91206	-22.4205	112.8805	2.0600	131.57679	3.23	3.526	.550	12.888	1.063
GRO-KC	Healthy Control	2299.2342	761.40974	724.1381	3874.3302	1372.0000	3730.13072	989.75	4.645	.472	22.225	.918
	mTBI	5865.6108	810.33379	4244.1367	7487.0850	2574.5000	6276.81851	8107.75	1.412	.309	.951	.608
	Orthopedic Injury	3010.7747	780.12599	1356.9815	4664.5679	1498.0000	3216.54187	3362.00	2.596	.550	7.845	1.063
IFN-α2	Healthy Control	30.2183	21.92236	-15.1315	75.5682	3.3700	107.39721	5.54	4.699	.472	22.522	.918
	mTBI	10.5860	5.18907	.2027	20.9693	3.3700	40.19435	6.35	6.883	.309	49.885	.608
	Orthopedic Injury	112.8118	106.02283	-111.9466	337.5701	4.7900	437.14334	10.33	4.122	.550	16.991	1.063
IFN-γ	Healthy Control	31.7733	10.28205	10.5033	53.0434	9.2150	50.37155	30.28	2.662	.472	7.902	.918
	mTBI	29.2780	13.31713	2.6305	55.9255	7.9200	103.15402	14.80	5.458	.309	29.969	.608
	Orthopedic Injury	72.6647	36.54106	-4.7989	150.1283	7.9100	150.66264	17.43	2.553	.550	6.432	1.063
IL-10	Healthy Control	40.1200	36.27626	-34.9232	115.1632	.0300	177.71665	.26	4.857	.472	23.706	.918
	mTBI	8.9607	5.97769	-3.0007	20.9220	.1100	46.30300	1.43	7.215	.309	53.921	.608
	Orthopedic Injury	1.1094	.53513	-.0250	2.2438	.3200	2.20640	1.07	3.453	.550	12.850	1.063
IL-12 p40	Healthy Control	1.7729	1.03523	-.3686	3.9145	.0000	5.07157	.00	2.891	.472	7.431	.918
	mTBI	24.3778	17.74235	-11.1245	59.8802	.0000	137.43167	.18	7.147	.309	52.938	.608
	Orthopedic Injury	19.2629	13.46830	-9.2886	47.8145	.0000	55.53124	6.86	3.579	.550	13.350	1.063

IL-12 p70	Healthy Control	26.8788	12.91554	.1609	53.5966	.2800	63.27295	14.60	2.685	.472	6.461	.918
	mTBI	71.5728	51.11569	-30.7094	173.8551	.4000	395.94046	2.63	6.614	.309	45.785	.608
	Orthopedic Injury	70.8688	45.21690	-24.9867	166.7244	.2800	186.43407	.81	2.589	.550	5.472	1.063
IL-13	Healthy Control	3.4642	1.40829	.5509	6.3774	.2000	6.89920	2.94	3.080	.472	10.792	.918
	mTBI	21.3277	10.60627	.1046	42.5508	.1200	82.15582	5.14	6.238	.309	42.766	.608
	Orthopedic Injury	44.8159	28.33216	-15.2456	104.8774	7.9100	116.81648	14.54	3.207	.550	10.411	1.063
IL-15	Healthy Control	1.3929	.83498	-.3344	3.1202	.2400	4.09056	.36	4.370	.472	19.854	.918
	mTBI	1.6173	.60920	.3983	2.8363	.5050	4.71884	.65	6.025	.309	39.530	.608
	Orthopedic Injury	12.1847	7.05726	-2.7760	27.1454	.6000	29.09784	1.97	2.493	.550	4.979	1.063
IL-17A	Healthy Control	7.8792	1.98898	3.7647	11.9937	4.0000	9.74396	8.30	1.758	.472	2.539	.918
	mTBI	13.6777	5.57217	2.5278	24.8275	2.3850	43.16181	6.92	4.963	.309	25.568	.608
	Orthopedic Injury	17.8424	8.13366	.5998	35.0849	2.4500	33.53595	9.51	2.388	.550	4.795	1.063
IL-1α	Healthy Control	191.7713	70.49334	45.9447	337.5978	36.9750	345.34543	147.32	1.928	.472	2.655	.918
	mTBI	102.6905	53.30999	-3.9825	209.3635	3.9100	412.93740	40.61	5.392	.309	29.319	.608
	Orthopedic Injury	270.9147	126.49981	2.7471	539.0823	56.2600	521.57207	143.41	2.350	.550	4.815	1.063
IL1β	Healthy Control	1.5413	.62299	.2525	2.8300	.2850	3.05203	.57	2.843	.472	8.219	.918
	mTBI	1.1713	.61751	-.0643	2.4070	.1750	4.78325	.13	5.316	.309	27.597	.608
	Orthopedic Injury	3.6912	2.14214	-.8500	8.2323	.6800	8.83226	.81	2.868	.550	7.675	1.063
IL-2	Healthy Control	2.9875	1.32048	.2559	5.7191	.5050	6.46902	3.01	3.348	.472	11.927	.918
	mTBI	9.4870	7.59170	-5.7040	24.6780	.1600	58.80505	.24	7.558	.309	57.888	.608
	Orthopedic Injury	12.5882	6.63669	-1.4809	26.6574	.4600	27.36378	1.38	2.073	.550	3.010	1.063
IL-3	Healthy Control	.0729	.00788	.0566	.0892	.0600	.03862	.03	1.710	.472	2.170	.918
	mTBI	.0623	.00478	.0528	.0719	.0550	.03702	.03	2.773	.309	9.344	.608
	Orthopedic Injury	.1282	.02017	.0855	.1710	.1000	.08316	.14	.680	.550	-.121	1.063
IL-4	Healthy Control	13.2567	1.84862	9.4325	17.0808	11.8900	9.05634	13.60	1.030	.472	.781	.918
	mTBI	7.7718	.96210	5.8467	9.6970	5.8800	7.45242	11.79	.946	.309	.360	.608

	Orthopedic Injury	12.2000	1.84046	8.2984	16.1016	13.0800	7.58843	16.01	-.120	.550	-1.566	1.063
IL-5	Healthy Control	1.6150	1.01253	-.4796	3.7096	.1300	4.96035	.27	3.611	.472	12.929	.918
	mTBI	.9265	.67734	-.4289	2.2819	.1100	5.24664	.08	7.568	.309	58.010	.608
	Orthopedic Injury	1.5212	.69550	.0468	2.9956	.5400	2.86762	.64	2.540	.550	5.206	1.063
IL-6	Healthy Control	7.2596	3.11135	.8233	13.6959	1.2250	15.24242	5.23	2.982	.472	8.951	.918
	mTBI	12.6450	5.10998	2.4200	22.8700	3.2500	39.58175	12.77	6.951	.309	51.452	.608
	Orthopedic Injury	25.9453	10.43963	3.8143	48.0763	6.4700	43.04368	30.79	1.957	.550	3.070	1.063
IL-7	Healthy Control	14.9037	6.58637	1.2788	28.5287	1.2900	32.26649	6.05	2.837	.472	8.069	.918
	mTBI	10.6817	7.06658	-3.4585	24.8219	2.0600	54.73753	3.43	7.652	.309	59.001	.608
	Orthopedic Injury	17.5724	10.65471	-5.0146	40.1593	3.7200	43.93049	4.97	3.329	.550	11.403	1.063
IL8_CXCL8	Healthy Control	10.3858	2.63075	4.9437	15.8280	5.5700	12.88798	9.10	2.853	.472	9.937	.918
	mTBI	13.9988	3.05252	7.8907	20.1069	5.4250	23.64475	9.07	2.972	.309	8.606	.608
	Orthopedic Injury	14.9800	4.66012	5.1010	24.8590	5.1600	19.21418	17.20	2.016	.550	3.329	1.063
IL-9	Healthy Control	2.1979	1.67796	-1.2732	5.6690	.0600	8.22029	.16	4.480	.472	20.717	.918
	mTBI	1.2648	.78319	-.3023	2.8320	.0600	6.06659	.07	6.676	.309	47.266	.608
	Orthopedic Injury	1.8576	1.38418	-1.0767	4.7920	.2100	5.70711	.78	4.077	.550	16.726	1.063
IP-10	Healthy Control	160.6879	16.45299	126.6523	194.7235	156.8400	80.60287	93.16	1.355	.472	2.537	.918
	mTBI	268.8803	45.94778	176.9390	360.8216	162.2250	355.90999	207.91	3.624	.309	16.469	.608
	Orthopedic Injury	224.8906	58.93160	99.9612	349.8200	174.4400	242.98123	225.10	2.980	.550	10.425	1.063
LIGHT/TNFSF14	Healthy Control	30.9442	5.58282	19.3952	42.4931	20.5300	27.35011	34.43	1.710	.472	2.990	.918
	mTBI	71.6242	6.15730	59.3034	83.9449	60.5100	47.69423	63.24	1.277	.309	1.390	.608
	Orthopedic Injury	60.4718	15.39341	27.8392	93.1043	45.5700	63.46865	49.94	1.862	.550	2.956	1.063
Lipocalin-2	Healthy Control	24329.3333	1260.97114	21720.8158	26937.8509	25325.0000	6177.47173	6440.25	-1.233	.472	.955	.918
	mTBI	27192.1333	603.35753	25984.8177	28399.4490	28549.0000	4673.58731	4389.00	-1.868	.309	5.047	.608
	Orthopedic Injury	23530.3529	2177.01770	18915.2816	28145.4243	25325.0000	8976.07392	12159.50	-.887	.550	-.324	1.063

MCP-1	Healthy Control	398.7767	37.38330	321.4434	476.1099	392.5350	183.14002	198.61	.436	.472	.386	.918
	mTBI	505.1280	38.41467	428.2604	581.9956	465.2900	297.55879	388.62	.941	.309	.279	.608
	Orthopedic Injury	432.4694	71.41782	281.0704	583.8684	335.4400	294.46320	440.66	.987	.550	-.076	1.063
MCP-3	Healthy Control	7.0750	2.23027	2.4613	11.6887	1.5800	10.92605	12.02	2.103	.472	4.944	.918
	mTBI	7.6912	2.77760	2.1332	13.2491	2.8900	21.51519	6.28	6.079	.309	41.522	.608
	Orthopedic Injury	13.4306	6.68455	-.7400	27.6012	3.2800	27.56112	15.64	3.247	.550	11.519	1.063
MDC	Healthy Control	1096.0300	71.03103	949.0911	1242.9689	1094.0000	347.97954	453.29	.212	.472	.706	.918
	mTBI	1168.3527	57.58227	1053.1308	1283.5745	1217.0000	446.03037	635.60	.128	.309	-.371	.608
	Orthopedic Injury	991.0824	138.41446	697.6568	1284.5079	731.4300	570.69743	438.83	1.943	.550	3.300	1.063
MIF	Healthy Control	908.5663	76.24589	750.8396	1066.2929	983.7900	373.52704	474.60	-.382	.472	-.247	.918
	mTBI	1355.2622	269.58861	815.8166	1894.7077	1056.0000	2088.22438	502.43	7.304	.309	55.407	.608
	Orthopedic Injury	868.1253	94.78610	667.1877	1069.0628	805.8200	390.81309	470.73	.421	.550	.584	1.063
MIP-1α	Healthy Control	46.5729	37.88316	-31.7944	124.9402	8.3850	185.58882	11.82	4.887	.472	23.915	.918
	mTBI	63.8878	36.85795	-9.8648	137.6404	5.2900	285.50045	9.39	6.613	.309	46.370	.608
	Orthopedic Injury	57.2194	32.92359	-12.5755	127.0143	5.1600	135.74745	19.74	2.670	.550	6.101	1.063
MIP-1β	Healthy Control	35.5054	5.82294	23.4597	47.5511	27.8200	28.52648	31.51	1.716	.472	3.544	.918
	mTBI	42.7562	9.50950	23.7277	61.7846	26.7950	73.66025	15.61	5.904	.309	37.724	.608
	Orthopedic Injury	46.4459	12.90413	19.0903	73.8014	23.9300	53.20511	14.43	2.685	.550	7.436	1.063
MMP-1	Healthy Control	1895.5108	406.09695	1055.4353	2735.5864	1235.5000	1989.46065	1576.85	1.998	.472	3.368	.918
	mTBI	2365.3608	183.42763	1998.3230	2732.3987	2215.0000	1420.82431	1653.00	.898	.309	.737	.608
	Orthopedic Injury	2226.9241	407.85165	1362.3172	3091.5310	1756.0000	1681.61542	3023.47	.731	.550	-.634	1.063
MMP-9	Healthy Control	45096.8333	8167.43309	28201.2107	61992.4560	32747.5000	40012.08717	45552.25	2.494	.472	8.518	.918
	mTBI	147342.5833	15576.10316	116174.8729	178510.2938	95252.0000	120651.97626	249351.75	.440	.309	-1.491	.608
	Orthopedic Injury	88801.2353	19647.97932	47149.3798	130453.0908	69148.0000	81010.69405	62133.00	1.750	.550	2.625	1.063
MPO	Healthy Control	2869137.2083	486292.12203	1863165.3092	3875109.1075	1665564.0000	2382335.12981	3266271.25	1.570	.472	2.969	.918

	mTBI	5788575.1167	460546.23773	4867024.2236	6710126.0098	4940279.0000	3567375.81776	5989367.25	.619	.309	-.299	.608
	Orthopedic Injury	4622337.9412	673120.38290	3195386.4745	6049289.4079	3840466.0000	2775346.43744	3656754.50	.964	.550	.526	1.063
NCAM	Healthy Control	713405.0000	64157.05958	580686.0104	846123.9896	579402.0000	314304.11876	280622.00	1.427	.472	.938	.918
	mTBI	589206.9167	27381.48473	534416.6923	643997.1411	573127.0000	212096.06871	267513.25	.563	.309	1.049	.608
	Orthopedic Injury	464424.8235	47291.09352	364172.1838	564677.4633	451619.0000	194986.17373	210490.00	-.413	.550	.225	1.063
Neuropilin-1	Healthy Control	58917.5833	1442.86938	55932.7806	61902.3861	60526.5000	7068.58749	11891.75	-.253	.472	-.852	.918
	mTBI	59644.7167	1400.70583	56841.9108	62447.5226	60849.0000	10849.82071	14276.25	-.651	.309	.168	.608
	Orthopedic Injury	61810.1176	2454.27167	56607.2941	67012.9412	65258.0000	10119.22134	19174.50	-.458	.550	-1.290	1.063
NRG-1 β1	Healthy Control	27.1133	19.14800	-12.4973	66.7240	5.6800	93.80564	3.43	4.836	.472	23.551	.918
	mTBI	7.1440	.35236	6.4389	7.8491	6.6000	2.72939	3.39	1.526	.309	3.027	.608
	Orthopedic Injury	6.4629	1.56655	3.1420	9.7839	4.4200	6.45905	2.11	3.775	.550	14.875	1.063
PAI 1	Healthy Control	567883.8333	58622.12710	446614.7240	689152.9427	494155.0000	287188.59809	317411.75	1.607	.472	2.709	.918
	mTBI	515174.2000	29842.49438	455459.5067	574888.8933	449692.0000	231158.96747	272212.50	1.054	.309	1.975	.608
	Orthopedic Injury	421308.1765	47363.36565	320902.3266	521714.0263	374507.0000	195284.15936	211611.50	.470	.550	.532	1.063
PARK5-UCHL1	Healthy Control	255.4921	19.22702	215.7180	295.2662	233.4500	94.19276	82.51	2.485	.472	6.132	.918
	mTBI	277.2775	23.60385	230.0463	324.5087	233.4500	182.83463	90.54	5.516	.309	36.039	.608
	Orthopedic Injury	280.4482	55.46450	162.8687	398.0277	218.3600	228.68599	74.47	3.852	.550	15.404	1.063
PDGF AA	Healthy Control	10650.2500	1221.86872	8122.6220	13177.8780	8428.5000	5985.90978	5589.00	2.162	.472	4.781	.918
	mTBI	7756.2888	552.04637	6651.6466	8860.9311	7087.0000	4276.13278	5181.25	.788	.309	.498	.608
	Orthopedic Injury	8567.5835	878.07418	6706.1494	10429.0176	8801.0000	3620.39260	4515.00	-.212	.550	1.178	1.063
PDGF-AB BB	Healthy Control	48204.7917	5321.27743	37196.8906	59212.6927	44441.5000	26068.82895	46242.00	.550	.472	-.673	.918
	mTBI	52717.5333	4990.83719	42730.8912	62704.1755	39918.0000	38658.85862	39463.00	2.514	.309	8.427	.608
	Orthopedic Injury	35532.1176	4480.92243	26032.9864	45031.2489	25884.0000	18475.31650	32232.00	.519	.550	-1.192	1.063
RAGE	Healthy Control	1381.4288	159.09555	1052.3145	1710.5430	1095.0000	779.40585	1097.74	.839	.472	-.068	.918

	mTBI	1341.3432	82.41043	1176.4403	1506.2461	1203.0000	638.34847	894.93	1.348	.309	2.431	.608
	Orthopedic Injury	1289.6694	143.30551	985.8753	1593.4635	1289.0000	590.86376	893.05	.898	.550	1.070	1.063
RANTES	Healthy Control	145213.4583	15733.67547	112665.8708	177761.0458	116504.5000	77078.95336	142828.25	.231	.472	-1.458	.918
	mTBI	135967.5808	13884.35650	108185.0476	163750.1140	109824.0000	107547.76300	132251.00	1.751	.309	4.148	.608
	Orthopedic Injury	106904.8235	12164.73214	81116.7434	132692.9037	107414.0000	50156.47551	47272.00	.537	.550	1.149	1.063
S100B	Healthy Control	45.9458	6.37824	32.7514	59.1402	37.3550	31.24686	34.58	1.889	.472	3.657	.918
	mTBI	82.3167	10.41005	61.4862	103.1471	54.4000	80.63593	56.71	2.595	.309	7.331	.608
	Orthopedic Injury	54.0347	7.71680	37.6758	70.3936	41.6700	31.81717	51.99	.809	.550	-.643	1.063
sCD40L	Healthy Control	11238.6667	1635.77248	7854.8135	14622.5199	7659.0000	8013.61584	15170.00	.506	.472	-1.298	.918
	mTBI	14078.5500	769.60508	12538.5738	15618.5262	14941.5000	5961.33533	9390.50	-.323	.309	-.700	.608
	Orthopedic Injury	11593.7776	2408.08400	6488.8676	16698.6877	3812.0000	9928.78469	19703.00	.222	.550	-2.044	1.063
sICAM-1	Healthy Control	297116.3333	29974.57449	235109.2017	359123.4650	257692.0000	146844.82552	195898.50	1.152	.472	1.300	.918
	mTBI	369422.9042	54689.61668	259989.2340	478856.5744	261364.5000	423623.94920	171999.50	4.631	.309	25.934	.608
	Orthopedic Injury	212588.4118	35451.38624	137434.8302	287741.9933	170392.0000	146169.81003	127189.50	2.400	.550	7.617	1.063
sVCAM-1	Healthy Control	2502167.3750	313156.72853	1854353.3253	3149981.4247	1878480.0000	1534148.38882	1172552.00	1.808	.472	2.755	.918
	mTBI	2282165.8000	175002.25027	1931987.1061	2632344.4939	2093352.0000	1355561.60170	1141512.50	2.630	.309	8.444	.608
	Orthopedic Injury	1699389.5294	175888.39065	1326522.7980	2072256.2608	1858136.0000	725206.41295	1186732.00	-.433	.550	-.332	1.063
TGF-α	Healthy Control	2.7333	.62270	1.4452	4.0215	1.5400	3.05057	2.58	1.988	.472	3.390	.918
	mTBI	5.9473	1.51901	2.9078	8.9869	4.1400	11.76618	4.55	7.091	.309	53.151	.608
	Orthopedic Injury	5.1665	1.58962	1.7966	8.5363	2.5200	6.55416	2.79	3.207	.550	11.184	1.063
TNF-α	Healthy Control	5.0071	.24828	4.4935	5.5207	4.9100	1.21632	.95	.674	.472	3.303	.918
	mTBI	5.6492	.37186	4.9051	6.3933	5.4950	2.88044	2.22	2.706	.309	13.221	.608
	Orthopedic Injury	6.5624	1.04230	4.3528	8.7719	5.0300	4.29751	1.74	2.088	.550	4.092	1.063
VEGF	Healthy Control	10755.4167	890.43339	8913.4149	12597.4185	10377.5000	4362.21490	5487.75	.742	.472	.340	.918

	mTBI	11073.7833	690.30158	9692.4931	12455.0736	10205.5000	5347.05306	6604.75	.708	.309	.686	.608
	Orthopedic Injury	10865.7647	1122.26575	8486.6676	13244.8618	10924.0000	4627.22022	7509.00	.353	.550	-.661	1.063
VEGF-A	Healthy Control	28.3042	4.80800	18.3581	38.2503	26.2500	23.55430	35.35	.990	.472	1.016	.918
	mTBI	54.5882	5.75510	43.0722	66.1041	42.4500	44.57884	67.24	1.740	.309	5.155	.608
	Orthopedic Injury	34.2388	9.61700	13.8517	54.6259	19.2600	39.65190	52.13	1.305	.550	.873	1.063

Supplementary Table 3 S3. Shapiro-Wilk tests of normality for each biomarker by group.

Biomarker	Group	Statistic	df	Sig.
APP	Healthy Control	.942	24	.179
	mTBI	.958	60	.039
	Orthopedic Injury	.946	17	.400
BDNF	Healthy Control	.901	24	.023
	mTBI	.867	60	.000
	Orthopedic Injury	.939	17	.305
Catepsin-D	Healthy Control	.841	24	.001
	mTBI	.688	60	.000
	Orthopedic Injury	.865	17	.018
EGF	Healthy Control	.785	24	.000
	mTBI	.623	60	.000
	Orthopedic Injury	.810	17	.003
Enolase	Healthy Control	.935	24	.127
	mTBI	.893	60	.000
	Orthopedic Injury	.968	17	.783
Eotaxin	Healthy Control	.856	24	.003
	mTBI	.662	60	.000
	Orthopedic Injury	.413	17	.000
FGF	Healthy Control	.534	24	.000
	mTBI	.205	60	.000
	Orthopedic Injury	.518	17	.000
Fibroblast	Healthy Control	.928	24	.089
	mTBI	.955	60	.026
	Orthopedic Injury	.879	17	.031
Fracktalkine	Healthy Control	.750	24	.000
	mTBI	.794	60	.000

	Orthopedic Injury	.651	17	.000
GCSF	Healthy Control	.787	24	.000
	mTBI	.276	60	.000
	Orthopedic Injury	.535	17	.000
GM-CSF	Healthy Control	.293	24	.000
	mTBI	.380	60	.000
	Orthopedic Injury	.397	17	.000
GRO	Healthy Control	.352	24	.000
	mTBI	.776	60	.000
	Orthopedic Injury	.668	17	.000
IFN- α2	Healthy Control	.289	24	.000
	mTBI	.234	60	.000
	Orthopedic Injury	.274	17	.000
IFN-γ	Healthy Control	.633	24	.000
	mTBI	.256	60	.000
	Orthopedic Injury	.518	17	.000
IL-10	Healthy Control	.239	24	.000
	mTBI	.187	60	.000
	Orthopedic Injury	.499	17	.000
IL-12p40	Healthy Control	.399	24	.000
	mTBI	.172	60	.000
	Orthopedic Injury	.408	17	.000
IL-12p70	Healthy Control	.489	24	.000
	mTBI	.177	60	.000
	Orthopedic Injury	.430	17	.000
IL-13	Healthy Control	.565	24	.000
	mTBI	.275	60	.000
	Orthopedic Injury	.432	17	.000
IL-15	Healthy Control	.332	24	.000
	mTBI	.292	60	.000
	Orthopedic Injury	.457	17	.000
IL-17A	Healthy Control	.758	24	.000
	mTBI	.320	60	.000
	Orthopedic Injury	.553	17	.000
IL-1α	Healthy Control	.614	24	.000
	mTBI	.252	60	.000
	Orthopedic Injury	.561	17	.000
IL-1β	Healthy Control	.528	24	.000

	mTBI	.216	60	.000
	Orthopedic Injury	.443	17	.000
IL-2	Healthy Control	.501	24	.000
	mTBI	.146	60	.000
	Orthopedic Injury	.514	17	.000
IL-3	Healthy Control	.757	24	.000
	mTBI	.705	60	.000
	Orthopedic Injury	.918	17	.137
IL-4	Healthy Control	.900	24	.022
	mTBI	.886	60	.000
	Orthopedic Injury	.903	17	.076
IL-5	Healthy Control	.344	24	.000
	mTBI	.146	60	.000
	Orthopedic Injury	.493	17	.000
IL-6	Healthy Control	.523	24	.000
	mTBI	.281	60	.000
	Orthopedic Injury	.627	17	.000
IL-7	Healthy Control	.522	24	.000
	mTBI	.158	60	.000
	Orthopedic Injury	.435	17	.000
IL8 CXCL8	Healthy Control	.677	24	.000
	mTBI	.548	60	.000
	Orthopedic Injury	.665	17	.000
IL-9	Healthy Control	.291	24	.000
	mTBI	.205	60	.000
	Orthopedic Injury	.327	17	.000
IP-10	Healthy Control	.898	24	.019
	mTBI	.598	60	.000
	Orthopedic Injury	.629	17	.000
LIGHT/TNFSF14	Healthy Control	.824	24	.001
	mTBI	.883	60	.000
	Orthopedic Injury	.754	17	.001
Lipocalin-2	Healthy Control	.870	24	.005
	mTBI	.848	60	.000
	Orthopedic Injury	.870	17	.022
MCP-1	Healthy Control	.978	24	.856
	mTBI	.918	60	.001
	Orthopedic Injury	.854	17	.012

MCP-3	Healthy Control	.704	24	.000
	mTBI	.349	60	.000
	Orthopedic Injury	.527	17	.000
MDC	Healthy Control	.986	24	.974
	mTBI	.983	60	.567
	Orthopedic Injury	.729	17	.000
MIF	Healthy Control	.975	24	.800
	mTBI	.256	60	.000
	Orthopedic Injury	.958	17	.600
MIP-1α	Healthy Control	.239	24	.000
	mTBI	.218	60	.000
	Orthopedic Injury	.451	17	.000
MIP-1β	Healthy Control	.830	24	.001
	mTBI	.324	60	.000
	Orthopedic Injury	.556	17	.000
MMP-1	Healthy Control	.720	24	.000
	mTBI	.940	60	.006
	Orthopedic Injury	.892	17	.050
MMP-9	Healthy Control	.760	24	.000
	mTBI	.849	60	.000
	Orthopedic Injury	.783	17	.001
MPO	Healthy Control	.835	24	.001
	mTBI	.940	60	.005
	Orthopedic Injury	.925	17	.178
NCAM	Healthy Control	.791	24	.000
	mTBI	.949	60	.014
	Orthopedic Injury	.967	17	.768
Neuropilin-1	Healthy Control	.965	24	.545
	mTBI	.960	60	.048
	Orthopedic Injury	.902	17	.074
NRG1-β1	Healthy Control	.256	24	.000
	mTBI	.874	60	.000
	Orthopedic Injury	.450	17	.000
PAI-1	Healthy Control	.842	24	.002
	mTBI	.937	60	.004
	Orthopedic Injury	.956	17	.556
PARK5-UCH	Healthy Control	.655	24	.000
	mTBI	.443	60	.000

	Orthopedic Injury	.440	17	.000
PDGF AA	Healthy Control	.740	24	.000
	mTBI	.951	60	.017
	Orthopedic Injury	.977	17	.927
PDGF AB BB	Healthy Control	.903	24	.025
	mTBI	.750	60	.000
	Orthopedic Injury	.891	17	.049
RAGE	Healthy Control	.912	24	.039
	mTBI	.899	60	.000
	Orthopedic Injury	.928	17	.200
RANTES	Healthy Control	.908	24	.032
	mTBI	.842	60	.000
	Orthopedic Injury	.954	17	.527
S100B	Healthy Control	.788	24	.000
	mTBI	.681	60	.000
	Orthopedic Injury	.863	17	.017
sCD40L	Healthy Control	.882	24	.009
	mTBI	.967	60	.099
	Orthopedic Injury	.774	17	.001
sICAM-1	Healthy Control	.898	24	.020
	mTBI	.499	60	.000
	Orthopedic Injury	.764	17	.001
sVCAM-1	Healthy Control	.769	24	.000
	mTBI	.714	60	.000
	Orthopedic Injury	.963	17	.682
TGF-α	Healthy Control	.709	24	.000
	mTBI	.289	60	.000
	Orthopedic Injury	.562	17	.000
TNF-α	Healthy Control	.904	24	.026
	mTBI	.770	60	.000
	Orthopedic Injury	.671	17	.000
VEGF	Healthy Control	.950	24	.269
	mTBI	.963	60	.062
	Orthopedic Injury	.955	17	.544
VEGF A	Healthy Control	.918	24	.053
	mTBI	.850	60	.000
	Orthopedic Injury	.819	17	.004

Supplementary Table 4 S4. Kruskal-Wallis test summary comparing biomarkers across groups.

Biomarker	Test	Total N	Test Statistic	Degree Of Freedom	Asymptotic Sig.(2-sided test)
APP	Independent-Samples Kruskal-Wallis Test	101	.042	2	.979
BDNF	Independent-Samples Kruskal-Wallis Test	101	8.711	2	.013
Catepsin-D	Independent-Samples Kruskal-Wallis Test	101	1.045	2	.593
EGF	Independent-Samples Kruskal-Wallis Test	101	11.719	2	.003
Enolase NSE	Independent-Samples Kruskal-Wallis Test	101	1.220	2	.543
Eotaxin	Independent-Samples Kruskal-Wallis Test	101	5.739	2	.057
FGF	Independent-Samples Kruskal-Wallis Test	101	2.970	2	.226
Fibroblast	Independent-Samples Kruskal-Wallis Test	101	.028	2	.986
Fractalkine	Independent-Samples Kruskal-Wallis Test	101	7.992	2	.018
GCSF	Independent-Samples Kruskal-Wallis Test	101	10.122	2	.006
GM-CSF	Independent-Samples Kruskal-Wallis Test	101	2.190	2	.335
GRO	Independent-Samples Kruskal-Wallis Test	101	12.109	2	.002
IFN-α2	Independent-Samples Kruskal-Wallis Test	101	3.024	2	.220
IFN-γ	Independent-Samples Kruskal-Wallis Test	101	3.833	2	.147
IL-10	Independent-Samples Kruskal-Wallis Test	101	4.853	2	.088
IL-12 p40	Independent-Samples Kruskal-Wallis Test	101	2.262	2	.323

IL-12 p70	Independent-Samples Kruskal-Wallis Test	101	.567	2	.753
IL-13	Independent-Samples Kruskal-Wallis Test	101	5.289	2	.071
IL-15	Independent-Samples Kruskal-Wallis Test	101	8.055	2	.018
IL-17A	Independent-Samples Kruskal-Wallis Test	101	3.587	2	.166
IL-1α	Independent-Samples Kruskal-Wallis Test	101	13.283	2	.001
IL-1β	Independent-Samples Kruskal-Wallis Test	101	17.278	2	.000
IL-2	Independent-Samples Kruskal-Wallis Test	101	11.115	2	.004
IL-3	Independent-Samples Kruskal-Wallis Test	101	10.662	2	.005
IL-4	Independent-Samples Kruskal-Wallis Test	101	10.647	2	.005
IL-5	Independent-Samples Kruskal-Wallis Test	101	20.793	2	.000
IL-6	Independent-Samples Kruskal-Wallis Test	101	5.709	2	.058
IL-7	Independent-Samples Kruskal-Wallis Test	101	1.468	2	.480
IL-8_CXCL8	Independent-Samples Kruskal-Wallis Test	101	.511	2	.775
IL-9	Independent-Samples Kruskal-Wallis Test	101	11.427	2	.003
IP-10	Independent-Samples Kruskal-Wallis Test	101	.354	2	.838
LIGHT/TNFSF14	Independent-Samples Kruskal-Wallis Test	101	20.024	2	.000
Lipocalin-2	Independent-Samples Kruskal-Wallis Test	101	6.139	2	.046
MCP-1	Independent-Samples Kruskal-Wallis Test	101	2.222	2	.329
MCP-3	Independent-Samples Kruskal-Wallis Test	101	.957	2	.620

MDC	Independent-Samples Kruskal-Wallis Test	101	4.796	2	.091
MIF	Independent-Samples Kruskal-Wallis Test	101	7.188	2	.027
MIP-1α	Independent-Samples Kruskal-Wallis Test	101	1.094	2	.579
MIP-1β	Independent-Samples Kruskal-Wallis Test	101	.040	2	.980
MMP-1	Independent-Samples Kruskal-Wallis Test	101	5.193	2	.075
MMP-9	Independent-Samples Kruskal-Wallis Test	101	14.432	2	.001
MPO	Independent-Samples Kruskal-Wallis Test	101	14.415	2	.001
NCAM	Independent-Samples Kruskal-Wallis Test	101	7.209	2	.027
Neuropilin-1	Independent-Samples Kruskal-Wallis Test	101	1.705	2	.426
NRG1- β1	Independent-Samples Kruskal-Wallis Test	101	10.383	2	.006
PAI-1	Independent-Samples Kruskal-Wallis Test	101	3.275	2	.194
PARK5-UCH	Independent-Samples Kruskal-Wallis Test	101	.703	2	.704
PDGF AA	Independent-Samples Kruskal-Wallis Test	101	6.153	2	.046
PDGF AB BB	Independent-Samples Kruskal-Wallis Test	101	4.840	2	.089
RAGE	Independent-Samples Kruskal-Wallis Test	101	.098	2	.952
RANTES	Independent-Samples Kruskal-Wallis Test	101	1.351	2	.509
S100B	Independent-Samples Kruskal-Wallis Test	101	7.628	2	.022
sCD40L	Independent-Samples Kruskal-Wallis Test	101	2.753	2	.253
sICAM 1	Independent-Samples Kruskal-Wallis Test	101	8.085	2	.018

sVCAM 1	Independent-Samples Kruskal-Wallis Test	101	2.843	2	.241
TGF-α	Independent-Samples Kruskal-Wallis Test	101	11.456	2	.003
TNF-α	Independent-Samples Kruskal-Wallis Test	101	1.507	2	.471
VEGF	One-Way ANOVA	101	.056	2	.972
VEGF A	Independent-Samples Kruskal-Wallis Test	101	9.415	2	.009

Supplementary Table 5 S5. Dunn's post hoc pairwise comparisons with Bonferroni adjustment for significant biomarkers.

Biomarker	Test	Comparison	Test Statistic	Std. Error	Std. Test Statistic	Sig.	Adj. Sig. (Bonferroni)
BDNF	Dunn's Test	mTBI – Healthy Control	12.271	7.074	1.735	.083	.248
		Orthopedic Injury – Healthy Control	27.366	9.285	2.947	.003	.010
		Orthopedic Injury – mTBI	15.096	8.047	1.876	.061	.182
EGF	Dunn's Test	mTBI – Healthy Control	15.600	7.076	2.205	.027	.082
		Healthy Control – Orthopedic Injury	-9.262	9.287	-.997	.319	.956
		mTBI – Orthopedic Injury	-24.862	8.049	-3.089	.002	.006
Fracktalkine	Dunn's Test	Healthy Control – mTBI	-13.083	7.075	-1.849	.064	.193
		Orthopedic Injury – Healthy Control	7.311	9.286	.787	.431	1.000
		Orthopedic Injury – mTBI	20.395	8.048	2.534	.011	.034
GCSF	Dunn's Test	Healthy Control – mTBI	-14.200	7.069	-2.009	.045	.134
		Healthy Control – Orthopedic Injury	-29.346	9.278	-3.163	.002	.005
		mTBI – Orthopedic Injury	-15.146	8.041	-1.883	.060	.179
GRO	Dunn's Test	Healthy Control – mTBI	-23.917	7.075	-3.380	.001	.002

		Healthy Control – Orthopedic Injury	-10.647	9.286	-1.147	.252	.755
		Orthopedic Injury – mTBI	13.270	8.049	1.649	.099	.298
IL15	Dunn's Test	Healthy Control – mTBI	-18.567	7.066	-2.628	.009	.026
		Healthy Control – Orthopedic Injury	-21.608	9.274	-2.330	.020	.059
		mTBI – Orthopedic Injury	-3.041	8.038	-.378	.705	1.000
IL-1α	Dunn's Test	mTBI – Healthy Control	14.917	7.033	2.121	.034	.102
		Healthy Control – Orthopedic Injury	-12.297	9.230	-1.332	.183	.548
		mTBI – Orthopedic Injury	-27.213	8.000	-3.402	.001	.002
IL-1β	Dunn's Test	mTBI – Healthy Control	17.483	7.055	2.478	.013	.040
		Healthy Control – Orthopedic Injury	-13.434	9.259	-1.451	.147	.440
		mTBI – Orthopedic Injury	-30.917	8.025	-3.853	.000	.000
IL-2	Dunn's Test	mTBI – Healthy Control	13.600	7.043	1.931	.053	.160
		Healthy Control – Orthopedic Injury	-11.363	9.244	-1.229	.219	.657
		mTBI – Orthopedic Injury	-24.963	8.012	-3.116	.002	.006
IL-3	Dunn's Test	mTBI – Healthy Control	9.333	7.012	1.331	.183	.550
		Healthy Control – Orthopedic Injury	-16.355	9.203	-1.777	.076	.227
		mTBI – Orthopedic Injury	-25.689	7.977	-3.220	.001	.004
IL-4	Dunn's Test	mTBI – Healthy Control	19.833	7.056	2.811	.005	.015
		Orthopedic Injury – Healthy Control	1.294	9.260	.140	.889	1.000
		mTBI – Orthopedic Injury	-18.539	8.026	-2.310	.021	.063
IL-5	Dunn's Test	mTBI – Healthy Control	14.225	7.033	2.023	.043	.129

		Healthy Control – Orthopedic Injury	-21.490	9.231	-2.328	.020	.060
		mTBI – Orthopedic Injury	-35.715	8.001	-4.464	.000	.000
IL-9	Dunn's Test	mTBI – Healthy Control	5.179	7.038	.736	.462	1.000
		Healthy Control – Orthopedic Injury	-21.869	9.237	-2.367	.018	.054
		mTBI – Orthopedic Injury	-27.048	8.006	-3.378	.001	.002
LIGHT	Dunn's Test	Healthy Control – mTBI	-31.304	7.076	-4.424	.000	.000
		Healthy Control – Orthopedic Injury	-17.126	9.287	-1.844	.065	.196
		Orthopedic Injury – mTBI	14.178	8.049	1.761	.078	.235
Lipocalin	Dunn's Test	Healthy Control – mTBI	-15.687	7.060	-2.222	.026	.079
		Healthy Control – Orthopedic Injury	-2.683	9.266	-.290	.772	1.000
		Orthopedic Injury – mTBI	13.005	8.031	1.619	.105	.316
MIF	Dunn's Test	Healthy Control – mTBI	-13.075	7.076	-1.848	.065	.194
		Orthopedic Injury – Healthy Control	5.797	9.288	.624	.533	1.000
		Orthopedic Injury – mTBI	18.872	8.050	2.344	.019	.057
MMP-9	Dunn's Test	Healthy Control – mTBI	-26.762	7.076	-3.782	.000	.000
		Healthy Control – Orthopedic Injury	-16.322	9.288	-1.757	.079	.237
		Orthopedic Injury – mTBI	10.440	8.050	1.297	.195	.584
MPO	Dunn's Test	Healthy Control – mTBI	-26.862	7.076	-3.796	.000	.000
		Healthy Control – Orthopedic Injury	-19.683	9.288	-2.119	.034	.102
		Orthopedic Injury – mTBI	7.180	8.050	.892	.372	1.000
NCAM	Dunn's Test	mTBI – Healthy Control	10.438	7.073	1.476	.140	.420

		Orthopedic Injury – Healthy Control	24.925	9.284	2.685	.007	.022
		Orthopedic Injury – mTBI	14.488	8.046	1.801	.072	.215
NRG1-β1	Dunn's Test	Healthy Control – mTBI	-10.771	7.061	-1.525	.127	.381
		Orthopedic Injury – Healthy Control	14.374	9.267	1.551	.121	.363
		Orthopedic Injury – mTBI	25.145	8.032	3.131	.002	.005
PDGF AA	Dunn's Test	mTBI – Healthy Control	16.592	7.075	2.345	.019	.057
		Orthopedic Injury – Healthy Control	5.556	9.286	.598	.550	1.000
		mTBI – Orthopedic Injury	-11.035	8.048	-1.371	.170	.511
S100B	Dunn's Test	Healthy Control – mTBI	-19.192	7.070	-2.715	.007	.020
		Healthy Control – Orthopedic Injury	-9.748	9.279	-1.050	.294	.881
		Orthopedic Injury – mTBI	9.444	8.043	1.174	.240	.721
TGF-α	Dunn's Test	Healthy Control – mTBI	-23.742	7.076	-3.355	.001	.002
		Healthy Control – Orthopedic Injury	-20.424	9.287	-2.199	.028	.084
		Orthopedic Injury – mTBI	3.318	8.050	.412	.680	1.000
VEGF A	Dunn's Test	Healthy Control – mTBI	-17.954	7.075	-2.538	.011	.033
		Orthopedic Injury – Healthy Control	.614	9.286	.066	.947	1.000
		Orthopedic Injury – mTBI	18.568	8.049	2.307	.021	.063
sICAM 1	Dunn's Test	Healthy Control – mTBI	-1.329	7.075	-.188	.851	1.000
		Orthopedic Injury – Healthy Control	21.153	9.286	2.278	.023	.068
		Orthopedic Injury – mTBI	22.482	8.048	2.793	.005	.016

Part 2 - Machine-Learning–Based Group Classification and Model Interpretation

S2.1 Leakage-Safe Preprocessing Pipeline

A fully leakage-safe preprocessing pipeline was implemented to ensure that no statistical information from the held-out test partitions influenced any stage of model training. This design prevents data contamination and optimistic bias, providing reliable estimates of generalization performance. All preprocessing operations were executed strictly within each outer training fold of the nested cross-validation framework.

Outlier handling, winsorization, imputation, and scaling were performed exclusively on the training portion of each fold. The learned transformations were then applied to the corresponding test data. This approach follows current best practices for machine-learning analyses in small clinical datasets with high-dimensional biomarker panels.

S2.1.1 Preprocessing Steps (applied inside each outer fold)

1. Quantile-based winsorization

Each continuous biomarker was clipped to the empirical 1st and 99th percentiles, computed from the training fold only, to attenuate the influence of extreme values without discarding features. This conservative strategy stabilizes distributions while preserving biological variability. The number of affected samples per biomarker was recorded and later aggregated across folds.

Reference: Tukey (1962) [31].

2. Median imputation

Missing values were replaced using the training-fold median of each biomarker after winsorization.
It guarantees that the test data are transformed using statistics not derived from themselves, preventing leakage and avoiding scale distortions.

Reference: Little & Rubin (2002) [32].

3. Robust scaling

Features were standardized using median–IQR scaling (RobustScaler), with statistics computed solely from the training data. This normalization is resilient to outliers and preserves relative differences among biomarkers.

Reference: Huber (1981) [33].

S2.1.2 Outputs of the preprocessing step

Across all outer folds, the preprocessing stage generated detailed quality-control summaries, including:

- Distribution of winsorized samples per biomarker;
- Aggregated counts of affected samples;
- Visual barplots summarizing winsorization intensity;
- Confirmation that no biomarkers were excluded—all were retained for downstream modeling.

These outputs provide complete transparency regarding the preprocessing pipeline and align with contemporary standards for minimizing bias in clinical machine-learning workflows.

S2.2 Nested Cross-Validation Framework

A nested cross-validation (nested CV) framework was implemented to obtain unbiased performance estimates while ensuring proper model selection and strict control of data leakage. All model selection and hyperparameter tuning were performed exclusively within the training data of each outer fold.

S2.2.1 Outer loop

- 5-fold *StratifiedKFold* was used to define the outer cross-validation splits.
- This outer loop provides an unbiased estimate of generalization performance on unseen data.
- Test folds remained completely untouched until the final evaluation step of each outer iteration.

S2.2.2 Inner loop

- A 3-fold *StratifiedKFold* was applied only within the outer training folds.
- Hyperparameter optimization was performed exclusively in this inner loop using a grid search.

- After selecting the best hyperparameter combination, the model was retrained on the entire outer training fold and then evaluated on the corresponding outer test fold.

S2.2.3 Hyperparameter search grid

The following hyperparameter grid was used, tailored to small-sample, moderate-dimensional biomarker datasets:

- n_estimators ∈ {300, 500}.
- max_depth ∈ {None, 8, 12}.
- min_samples_leaf ∈ {1, 2, 4}.
- max_features = 'sqrt'.
- bootstrap = True.
- All models were trained with random_state = 42.

This grid is both statistically principled and computationally feasible for nested CV in small biomedical datasets. The choice is informed by core Random Forest literature [11, 34, 35].

S2.3 Classification Tasks and Feature Sets

S2.3.1 Tasks

Both multiclass and binary classification settings were evaluated:

- Three-class model**:** Control (0), TBI (1), and Orthopedic injury (2).
- Binary pairwise tasks**:** 0×1: Control vs. TBI and 0×2: Control vs. Orthopedic injury.

The binary tasks quantify discriminative performance independently for each pair of diagnostic categories.

S2.3.2 Feature configurations

Two feature configurations were analyzed to quantify the added predictive value of demographic variables:

1. bio_only**:** All available serum biomarkers.
2. bio_demo**:** All biomarkers plus age and sex.

S2.4 Model Evaluation Metrics

For each outer fold, the following metrics were computed:

Primary metrics:

- Balanced accuracy.
- Macro-F1 score.
- ROC-AUC (one-vs-rest for the three-class model; standard AUC for binary tasks).
- PR-AUC (one-vs-rest for the three-class model; binary PR-AUC for pairwise tasks).

Secondary outputs:

- Confusion matrices.
- ROC curves for all classes.
- Precision–recall (PR) curves for all classes.
- Best hyperparameter combination selected in the inner CV for each outer fold.

Fold-aggregated metrics**:**

- Mean performance across outer folds.
- Standard deviation across outer folds.
- 95% bootstrap confidence intervals (n = 1000 resamples).

These confidence intervals provide a precise quantification of performance uncertainty and support a robust interpretation of the machine-learning results.

S2.5 SHAP Interpretability and Stability

SHAP-based interpretability analyses were conducted using a leakage-safe framework that is entirely robust to the nested cross-validation design. In all experiments, SHAP values were computed exclusively on the held-out test partitions of the outer folds, ensuring that no information from the training data influenced feature attribution estimates. Feature robustness was further evaluated through fold-wise rank variability and Top-k recurrence frequency, enabling a stability-oriented interpretation of model behavior.

Reference: Lundberg & Lee (2017) [12].

S2.5.1 Per-Fold SHAP Values

For each outer fold and each predictive class, a comprehensive set of SHAP-based interpretability outputs was computed:

- Beeswarm plots illustrating the distribution, direction, and magnitude of SHAP values across features;
- Per-feature mean absolute SHAP values (|SHAP|), quantifying feature importance within each fold;
- Per-fold feature rankings, obtained by sorting features in descending order of mean |SHAP|.

These fold-specific explanations enable the inspection of how feature importance varies across resampling iterations and form the basis for downstream stability analyses.

S2.5.2 Aggregated SHAP Results

Across the five outer folds, SHAP-derived quantities were aggregated to produce global interpretability summaries for each class:

- Aggregated beeswarm visualizations combining SHAP values across folds;
- Two-dimensional heatmaps of SHAP-based feature ranks across folds, enabling direct visualization of rank variability;
- Class-specific summaries highlighting consistently influential biomarkers.

These aggregated representations facilitate the identification of biomarkers whose importance is robust to resampling variability rather than driven by fold-specific effects.

S2.5.3 SHAP Stability Analysis

To explicitly quantify the robustness of feature importance across folds, a systematic SHAP stability analysis was performed:

1. Features were ranked within each fold in descending order of mean |SHAP|.
2. The Top-10 features were extracted per fold.
3. The number of folds in which each feature appeared among the Top-10 was counted;
4. Absolute and normalized frequencies (%) were computed across folds;
5. Stability patterns were visualized using:
    - Top-10 stability barplots, and
    - Rank heatmaps reflecting fold-wise variability in feature ordering.

This stability-oriented analysis distinguishes biomarkers that contribute to model predictions from those whose apparent importance is sensitive to sampling variation, a critical consideration in small-sample biomedical studies.

S2.6 Software and Reproducibility

All analyses were conducted using a fully reproducible computational environment:

- Python 3.10.
- scikit-learn ≥ 1.4 (model training, nested CV, metrics).
- SHAP ≥ 0.45 (interpretability analyses).
- Pandas, NumPy (data processing).
- matplotlib, seaborn (visualization).

Reproducibility measures:

- All algorithms executed with fixed random seeds (random_state = 42).
- All preprocessing transformations were fit only on training folds.
- Results are fully deterministic under the specified environment.

These reproducibility measures ensure transparent, repeatable, and verifiable analyses consistent with modern standards for machine-learning applications in biomedical research.

S3. Supplementary Results

S3.1 Preprocessing Outcomes

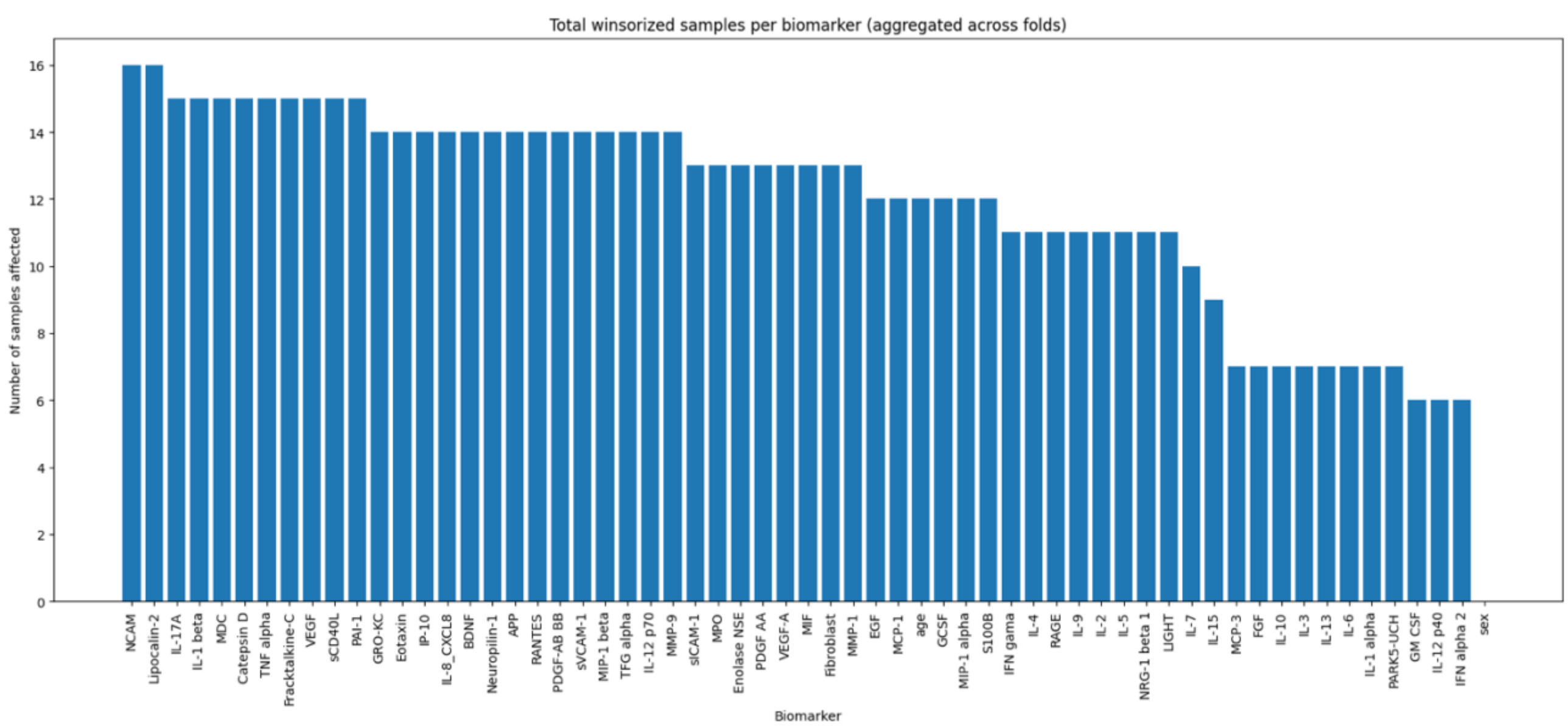

Supplementary Figure S3: Winsorized samples per biomarker across all folds.

Across all outer folds, the proportion of observations affected by winsorization ranged from approximately 6% to 16% per biomarker, with most biomarkers falling between 11% and 16%.

This reflects a moderate presence of extreme values within the sample (N=101), without evidence of widespread outlier inflation. Winsorization acted consistently across folds, indicating stable distributional properties independent of the resampling procedure. Significantly, this step operates at the observation level, ensuring that no biomarkers were removed and that the full biochemical panel remained available for downstream modeling. Biomarkers known to exhibit higher physiological variability showed slightly increased winsorization frequencies, which is expected given their broader biological ranges.

S3.2 Nested CV — Three-Class Performance

This section summarizes the nested cross-validation results for the three-class model**,** distinguishing Control (Class 0), TBI (Class 1), and Orthopedic Injury (Class 2).

Supplementary Table S4 — Per-fold performance metrics for the three-class classification task under both feature configurations (bio_demo and bio_only). Metrics include balanced accuracy, macro-F1, ROC-AUC, and PR-AUC.

Table S4 shows that both feature configurations, bio_demo (biomarkers plus age/sex) and bio_only (biomarkers only), performed similarly and stably across the five folds. The Balanced Accuracy score ranged from 0.53 to 0.70. Although some variability is normal in small datasets, no fold failed, which proves the robustness of our cross-validation design. The inclusion of age and sex did not improve performance. It suggests that age and sex provide only minor, non-dominant predictive information.

Supplementary Table S5 — Summary of outer-fold performance (mean ± 95% CI) for the three-class classification task under both feature configurations.

In Table S5, the aggregated fold results showed adequate and stable performance: (a) Mean Balanced Accuracy was 0.570 for bio_demo and 0.576 for bio_only, with significantly overlapping 95% confidence intervals (0.550–0.584 and 0.514–0.638, respectively); (b) ROC-AUC (OVR) remained high in both scenarios (0.862 for bio_demo and 0.850 for bio_only), confirming strong discriminative ability; (c) while PR-AUC (OVR) showed slightly more variability (0.786 for bio_demo and 0.776 for bio_only), overall performance was maintained within clinically meaningful ranges. These findings confirm model consistency and demonstrate that the inclusion of demographic features did not yield a performance gain, as bio_only achieved a comparable (and slightly higher) mean Balanced Accuracy, reinforcing the robust predictive value of the biomarker panel alone.

Supplementary Table S6 — Best Random Forest hyperparameters selected by inner cross-validation for each outer fold in the three-class classification task (Control, TBI, Orthopedic Injury).

In Table S6, the optimal Random Forest hyperparameters selected via inner cross-validation show a consistent preference for moderate ensemble sizes (300–500 trees), unrestricted tree depth (None), and minimal leaf sizes (1–2) across all folds. This stability in hyperparameter selection across both feature configurations indicates robust model complexity for the three-class classification task.

Supplementary Figure S2 — Confusion matrices for the three-class classification task under both scenarios (bio_demo and bio_only).

In Figure S2, both confusion matrices share the same qualitative pattern: (a) Class 1 (TBI) is the most accurately classified; (b) Class 0 (Control) shows moderate confusion with TBI; (c) Class 2 (Orthopedic Injury) is the most difficult to classify, exhibiting a distribution of misclassifications across both the Control and TBI groups. The similarity between feature configurations indicates that biomarkers remain the dominant signal, with minimal reliance on demographic factors.

Supplementary Figure S3 — ROC curves for all three classes under both modeling scenarios.

In Figure S3, the ROC analysis indicates: (a) AUC values are approximately 0.80 or higher for all classes across both scenarios; (b) TBI (Class 1) and Orthopedic Injury (Class 2) consistently display the highest AUCs, reaching up to 0.88–0.89; (c) Curves for bio_demo and bio_only are nearly superimposed, suggesting that age and sex add limited discriminative value. A high ROC-AUC underscores the Random Forest model's strong ranking performance, even in the presence of class imbalance.

Supplementary Figure S4 — Precision–recall curves for all three classes under both modeling scenarios.

In Figure S4, PR curves show more variability, consistent with class imbalance: (a) Class 1 (TBI) achieves the highest AUPRC (~0.88–0.91). (b) Classes 0 and 2 show lower AUPRC (~0.64–0.69) due to overlapping biomarker profiles. (c) Minimal differences between bio_demo and bio_only again highlight the predominance of biological signals. Overall, PR analysis confirms that the classifier performs best for TBI, where biomarker patterns are more distinctive.

Supplementary Table S4: Per-fold performance metrics for the three-class classification task under both feature configurations (bio_demo and bio_only). Metrics include balanced accuracy, macro-F1, ROC-AUC, and PR-AUC.

Scenario	Fold	Balanced Accuracy	Macro-F1	ROC-AUC (OVR)	PR-AUC (OVR)
bio_demo	1	0.567	0.593	0.907	0.824
	2	0.589	0.595	0.861	0.770
	3	0.533	0.508	0.829	0.706
	4	0.578	0.633	0.841	0.796
	5	0.583	0.631	0.872	0.832
bio_only	1	0.483	0.495	0.910	0.849
	2	0.589	0.612	0.862	0.773
	3	0.533	0.508	0.789	0.643
	4	0.689	0.743	0.841	0.799
	5	0.583	0.610	0.849	0.815

Supplementary Table S5: Summary of outer-fold performance (mean ± 95% CI) for the three-class classification task under both feature configurations.

Scenario	Metric	Mean	Std	CI95 Low	CI95 High
bio_demo	Balanced Accuracy	0.570	0.020	0.550	0.584
	Macro-F1	0.592	0.045	0.550	0.625
	ROC-AUC (OVR)	0.862	0.027	0.840	0.887
	PR-AUC (OVR)	0.786	0.046	0.742	0.822
bio_only	Balanced Accuracy	0.576	0.068	0.514	0.638
	Macro-F1	0.593	0.089	0.521	0.670
	ROC-AUC (OVR)	0.850	0.039	0.814	0.886
	PR-AUC (OVR)	0.776	0.071	0.703	0.827

Supplementary Table S6: Best Random Forest hyperparameters selected by inner cross-validation for each outer fold in the three-class classification task (Control, TBI, Orthopedic

Injury), shown separately for the two feature configurations (bio_demo and bio_only). Hyperparameters were selected exclusively within the inner cross-validation loop using grid search.

Scenario	Fold	n_estimators	max_depth	min_samples_leaf	max_features	bootstrap
bio_demo	1	300	None	1	'sqrt'	True
	2	500	None	1	'sqrt'	True
	3	300	None	2	'sqrt'	True
	4	300	None	2	'sqrt'	True
	5	500	None	1	'sqrt'	True
bio_only	1	300	None	1	'sqrt'	True
	2	300	None	1	'sqrt'	True
	3	500	None	1	'sqrt'	True
	4	500	None	1	'sqrt'	True
	5	300	None	1	'sqrt'	True

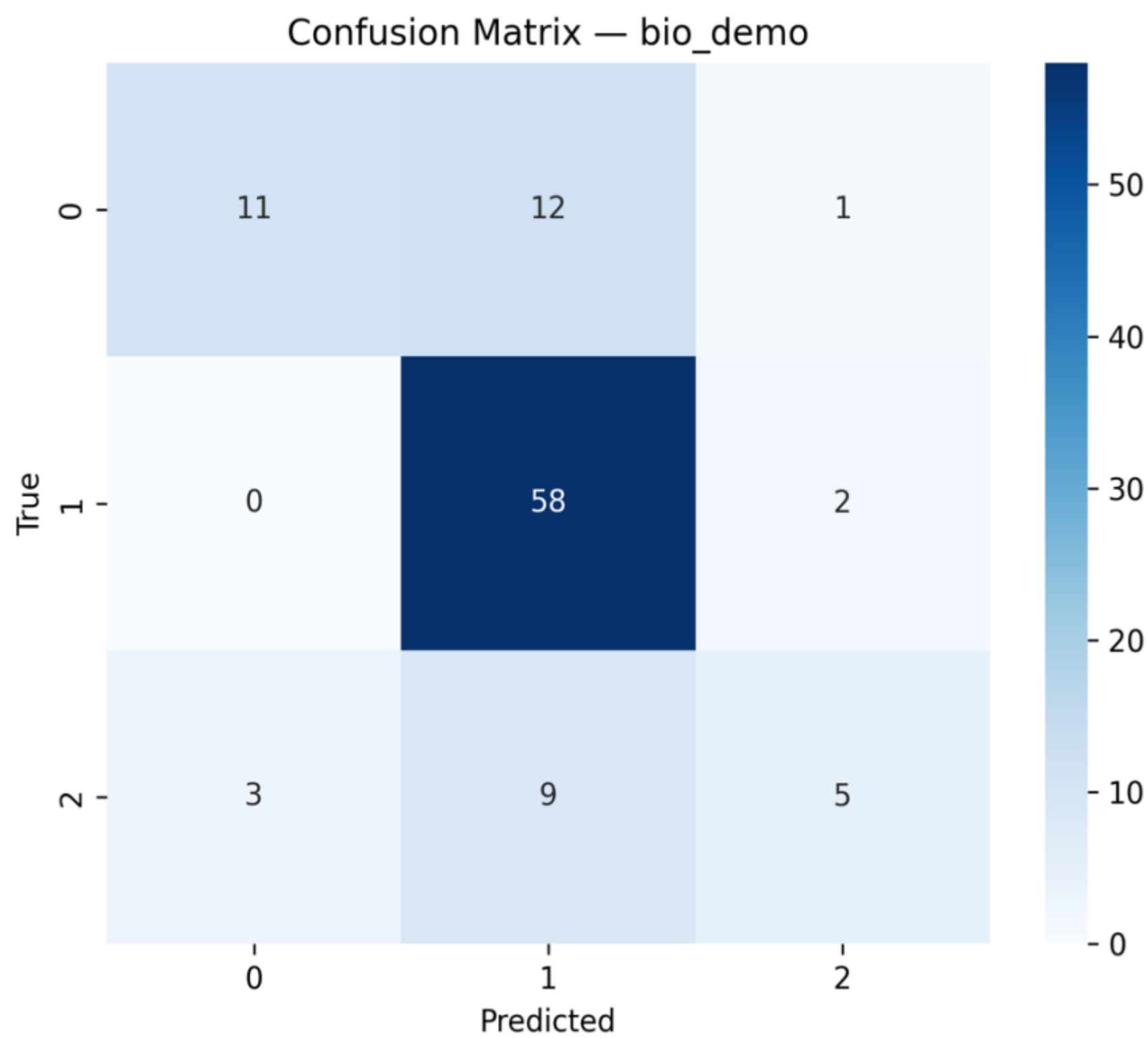

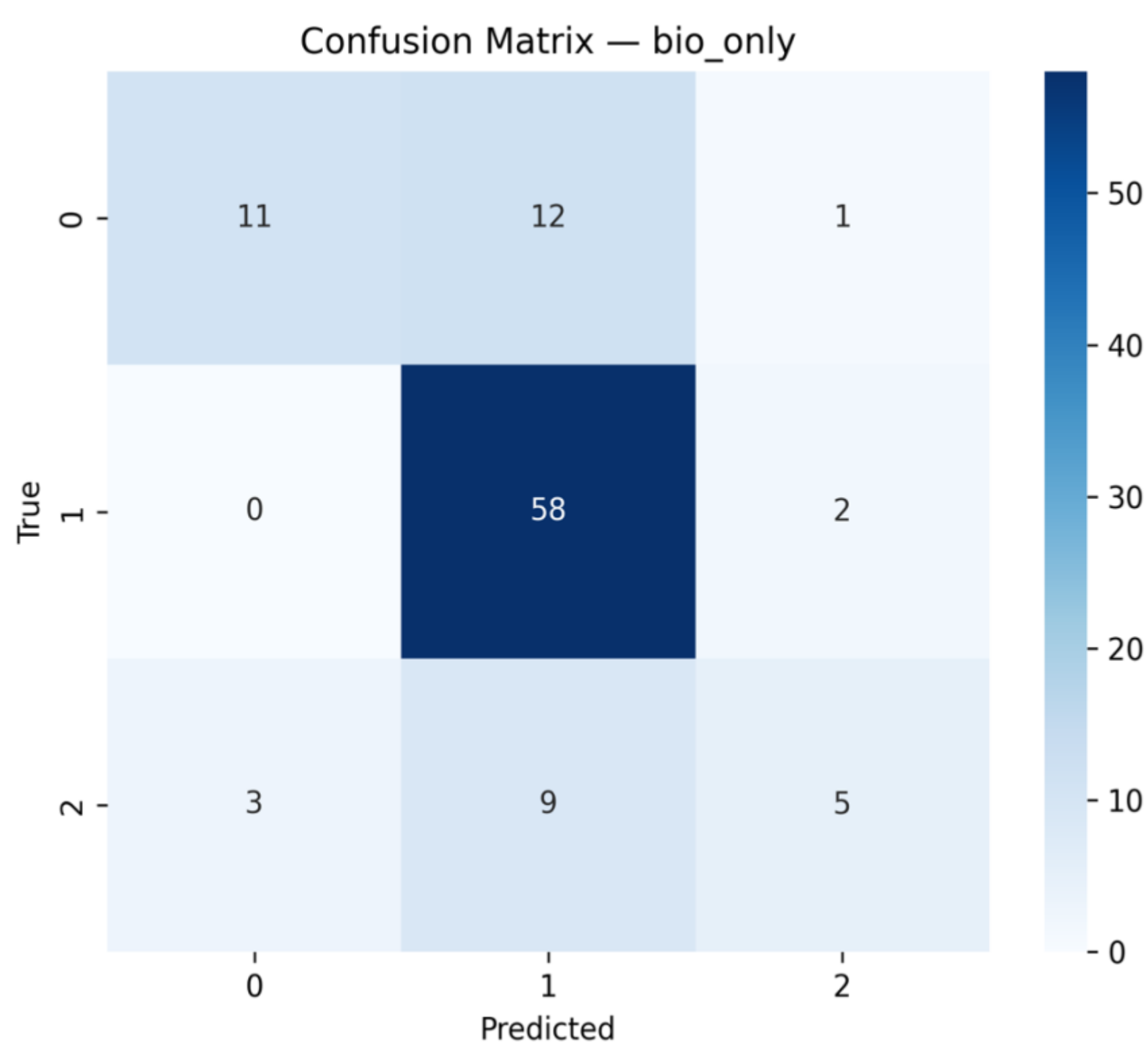

Supplementary Figure S2: Confusion matrices for the three-class classification task under both scenarios (bio_demo and bio_only).

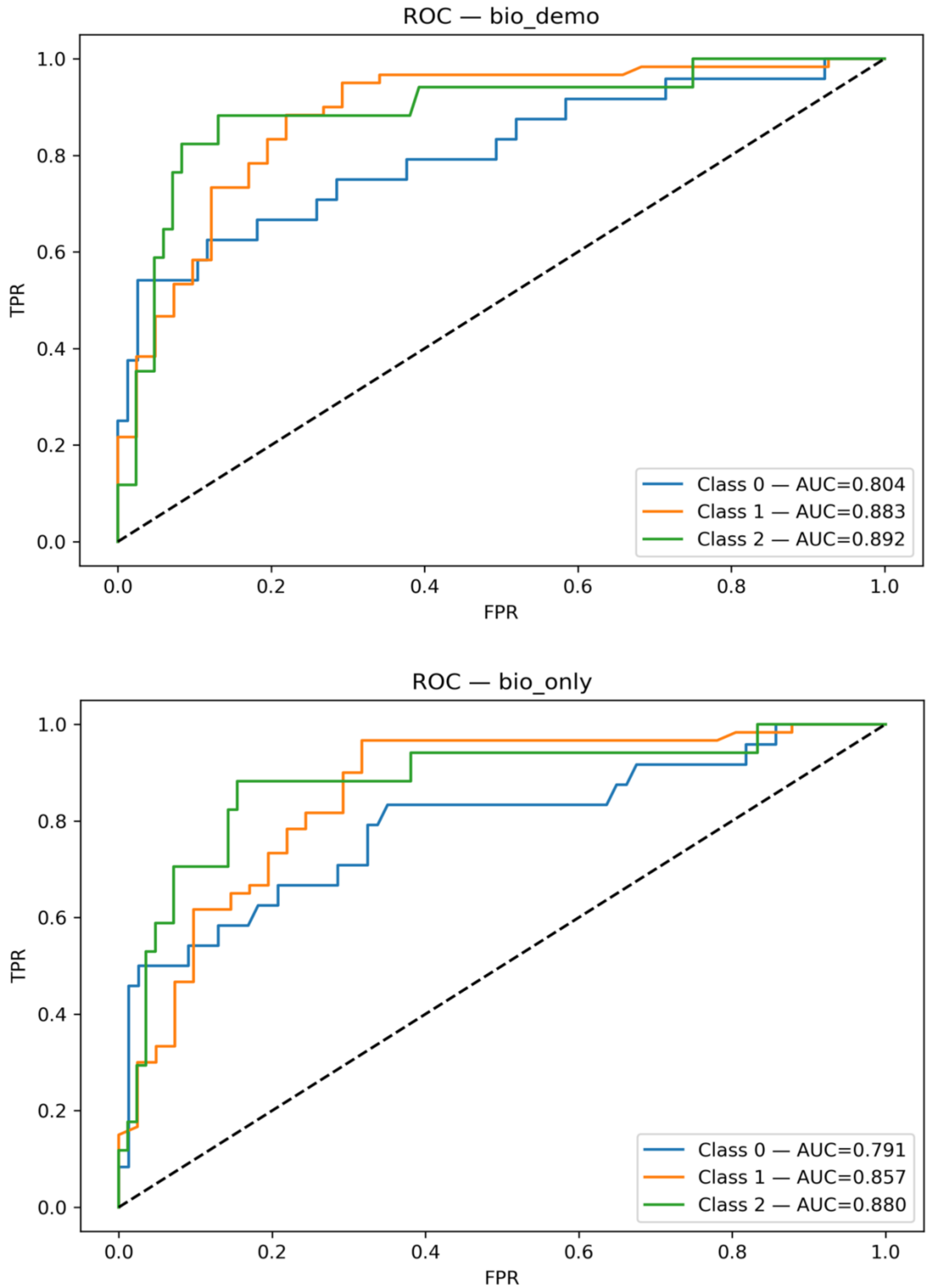

Supplementary Figure S3: ROC curves for the three-class classification task under bio_demo and bio_only scenarios. TPR: True Positive Rate; FPR: False Positive Rate.

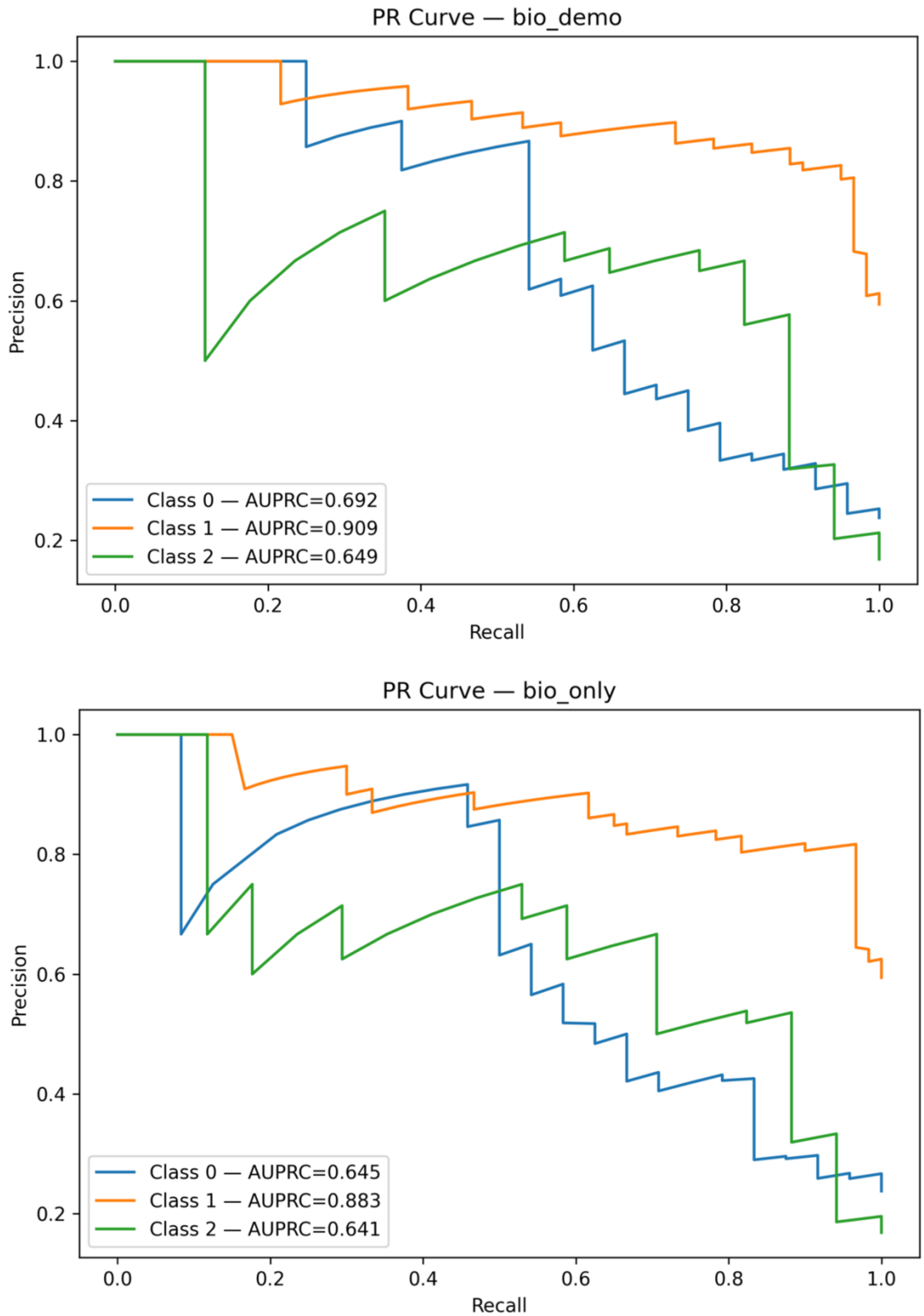

Supplementary Figure S4: Precision–recall curves for all three classes under both modeling scenarios.

S2.3 Binary Pairwise Classification

Binary analyses were conducted for:

- 0×1: Control vs. TBI;
- 0×2: Control vs. Orthopedic Injury.

Supplementary Table S7 – Per-fold performance metrics for binary tasks (0×1, 0×2) under both scenarios.

Table S7 analyzes binary classification performance, showing two distinct patterns: (1) Control vs. TBI (0×1 Pair): Performance was high and consistent across folds. Balanced Accuracy ranged from 0.70 to 0.90, and PR-AUC remained consistently high (0.83–0.99), confirming the strong discriminative power of the biomarker signature. Marginal differences between configurations suggest that demographic features add little benefit. (2) Control vs. Orthopedic Injury (0×2 Pair): Performance was significantly more variable, with Balanced Accuracy ranging from 0.40 to 0.80 and PR-AUC showing a wide spread (0.49 to 0.92). This increased variability reflects the greater biological overlap and classification difficulty between these two groups.

Supplementary Table S8 – Summary of binary classification performance (mean ± 95% CI).

In Table S8, the aggregated analysis confirms these patterns: (1) For Pair 0×1 (Control vs. TBI): Performance was robust with Balanced Accuracy of 0.76–0.77, ROC-AUC of 0.83, and high PR-AUC of ≈0.92. Relatively tight confidence intervals across all metrics indicate stable model generalization for this task. (2) For Pair 0×2 (Control vs. Orthopedic Injury): Classification was notably more difficult, with Balanced Accuracy ranging from 0.62 to 0.64. Interestingly, for this pair, the bio_only scenario achieved higher average ROC-AUC (0.83) and PR-AUC (0.82) compared to bio_demo (0.76 and 0.75, respectively), suggesting that demographic features may not enhance discrimination for orthopedic injuries. Broader intervals reflect the inherent challenges in separating these cohorts.

Supplementary Table S9: Best Random Forest hyperparameters selected by inner cross-validation for each outer fold in the binary classification task, Control vs. TBI (0×1).

In Table S9, for the Control vs. TBI task, inner cross-validation consistently selected moderate ensemble sizes (300–500 trees) with unrestricted tree depth and small leaf sizes. This stability across folds and feature configurations indicates a strong, reproducible signal that generalizes well, requiring minimal regularization to differentiate TBI from controls.

Supplementary Table S10: Best Random Forest hyperparameters selected by inner cross-validation for each outer fold in the binary classification task, Control vs. Orthopedic Injury (0×2).

In Table S10, hyperparameter selection for the more challenging Control vs. Orthopedic Injury task favored slightly larger leaf sizes (up to min_samples_leaf = 4) and stable ensemble settings (primarily 300 trees) across folds. This preference for increased regularization is consistent with the higher biological overlap between these cohorts, as the model seeks to minimize noise while maintaining a robust predictive signal.

Supplementary Figure S5 – Confusion matrices for the 0×1 classification task.

Figure S5 demonstrates high true-positive rates for the TBI class, exceeding 98% in both scenarios. While the Control group shows moderate confusion—with approximately 46% of instances misclassified as TBI—this pattern remains highly consistent across both feature configurations, confirming the robustness of the biological signal.

Supplementary Figure S6 – ROC curves for the 0×1 classification task.

Figure S6 shows ROC curves for the bio_demo and bio_only feature configurations. Class 0 (neg) represents Controls, and Class 1 (pos) represents TBI. ROC-AUC values are high (≈0.82), and the curves for both feature sets are nearly identical, indicating strong, consistent discriminative performance, with demographic features adding limited value.

Supplementary Figure S7 – Precision–recall curves for the 0×1 classification task.

Figure S7 shows that the TBI class maintains AUPRC ≈0.90 and the Control class AUPRC ≈0.76−0.79.

Supplementary Figure S8 – Confusion matrices for the 0×2 classification task.

Figure S8 reveals that while the model maintains a consistent detection rate for Controls (approximately 79% accuracy), the Orthopedic Injury class exhibits significantly higher misclassification rates. In both scenarios, more than half of the Orthopedic Injury group was misclassified as Controls, reflecting substantial biological overlap between these two non-neurological cohorts. The minimal variance between bio_demo and bio_only further confirms that this classification difficulty is driven by shared biomarker profiles rather than demographic factors.

Supplementary Figure S9 – ROC curves for the 0×2 classification task.

Figure S9 demonstrates moderate discriminative performance for the Control vs. Orthopedic Injury task, with ROC-AUC values ranging from 0.72 to 0.75 across feature configurations. The nearly overlapping curves for both modeling scenarios indicate that the biological signal provides

the primary basis for classification, while demographic variables contribute only marginally to class separability. This moderate performance is consistent with the anticipated biological proximity between healthy controls and patients with orthopedic injury.

Supplementary Figure S10 – Precision–recall curves for the 0×2 classification task.

Figure S10 presents the Precision-Recall (PR) curves for the Control vs. Orthopedic Injury task, with AUPRC values ranging from 0.64 to 0.82. The high consistency across modeling scenarios (differences < 0.02 in AUPRC) reinforces the conclusion that demographic features do not substantially enhance the predictive signal provided by the biomarker panel. The lower AUPRC observed for the Orthopedic Injury class (Class 2) relative to Controls (Class 0) underscores the greater challenge of identifying specific inflammatory signatures in this injury cohort compared to healthy individuals.

Supplementary Table S7: Per-fold performance metrics for binary tasks (0×1, 0×2) under both scenarios.

Pair	Scenario	Fold	Balanced Accuracy	Macro-F1	ROC-AUC	PR-AUC
0×1	bio_demo	1	0.700	0.730	0.867	0.941
		2	0.900	0.924	0.867	0.925
		3	0.800	0.837	0.967	0.988
		4	0.700	0.730	0.683	0.829
		5	0.750	0.795	0.750	0.893
	bio_only	1	0.700	0.730	0.867	0.941
		2	0.858	0.858	0.867	0.925
		3	0.800	0.837	0.917	0.965
		4	0.700	0.730	0.767	0.867
		5	0.750	0.795	0.750	0.891
0×2	bio_demo	1	0.400	0.308	0.500	0.493
		2	0.733	0.733	0.800	0.756
		3	0.567	0.564	0.733	0.700
		4	0.800	0.750	0.867	0.867
		5	0.625	0.564	0.875	0.917
	bio_only	1	0.525	0.500	0.800	0.771
		2	0.733	0.733	0.867	0.806
		3	0.567	0.564	0.733	0.722
		4	0.633	0.619	0.867	0.867
		5	0.750	0.733	0.875	0.917

Supplementary Table S8: Summary of binary classification performance (mean ± 95% CI).

Pair	Scenario	Metric	Mean	Std	CI95 Low	CI95 High
0×1	bio_demo	Balanced Accuracy	0.770	0.075	0.710	0.840
		Macro-F1	0.803	0.073	0.743	0.868
		ROC-AUC	0.827	0.099	0.733	0.907
		PR-AUC	0.915	0.053	0.864	0.960
	bio_only	Balanced Accuracy	0.762	0.061	0.710	0.815
		Macro-F1	0.790	0.053	0.743	0.837
		ROC-AUC	0.833	0.064	0.777	0.887
		PR-AUC	0.918	0.035	0.887	0.947
0×2	bio_demo	Balanced Accuracy	0.625	0.139	0.500	0.740
		Macro-F1	0.584	0.159	0.444	0.709
		ROC-AUC	0.755	0.137	0.622	0.857
		PR-AUC	0.746	0.148	0.617	0.864
	bio_only	Balanced Accuracy	0.642	0.089	0.563	0.720
		Macro-F1	0.630	0.092	0.549	0.710
		ROC-AUC	0.828	0.055	0.773	0.870
		PR-AUC	0.816	0.069	0.758	0.878

Supplementary Table S9: Best Random Forest hyperparameters selected by inner cross-validation for each outer fold in the binary classification task, Control vs. TBI (0×1), shown for both feature configurations (bio_demo and bio_only).

Scenario	Fold	n_estimators	max_depth	min_samples_leaf	max_features	bootstrap
bio_demo	1	500	None	1	'sqrt'	True
	2	300	None	4	'sqrt'	True
	3	500	None	1	'sqrt'	True
	4	500	None	4	'sqrt'	True
	5	500	None	1	'sqrt'	True
bio_only	1	300	None	1	'sqrt'	True
	2	500	None	2	'sqrt	True
	3	300	None	1	'sqrt'	True
	4	500	None	1	'sqrt'	True
	5	300	None	1	'sqrt'	True

Supplementary Table S10: Best Random Forest hyperparameters selected by inner cross-validation for each outer fold in the binary classification task, Control vs. Orthopedic Injury (0×2), shown for both feature configurations (bio_demo and bio_only).

Scenario	Fold	n_estimators	max_depth	min_samples_leaf	max_features	bootstrap
bio_demo	1	300	None	4	'sqrt'	True
	2	300	None	2	'sqrt'	True
	3	300	None	2	'sqrt'	True
	4	300	None	1	'sqrt'	True
	5	300	None	4	'sqrt'	True
bio_only	1	300	None	1	'sqrt'	True
	2	300	None	4	'sqrt'	True
	3	300	None	1	'sqrt'	True
	4	300	None	1	'sqrt'	True
	5	500	None	2	'sqrt'	True

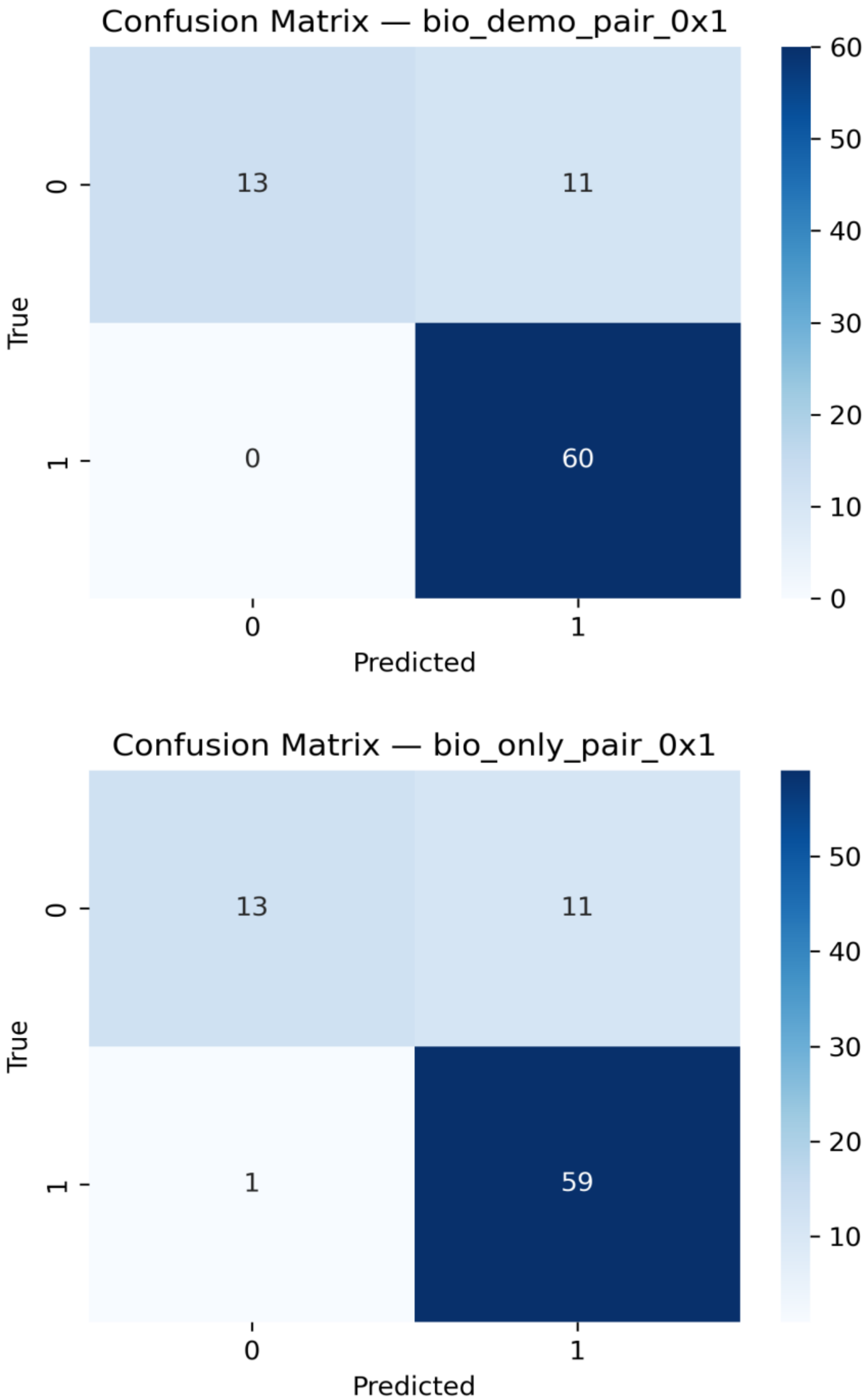

Supplementary Figure S5: Confusion matrices for the 0×1 classification task

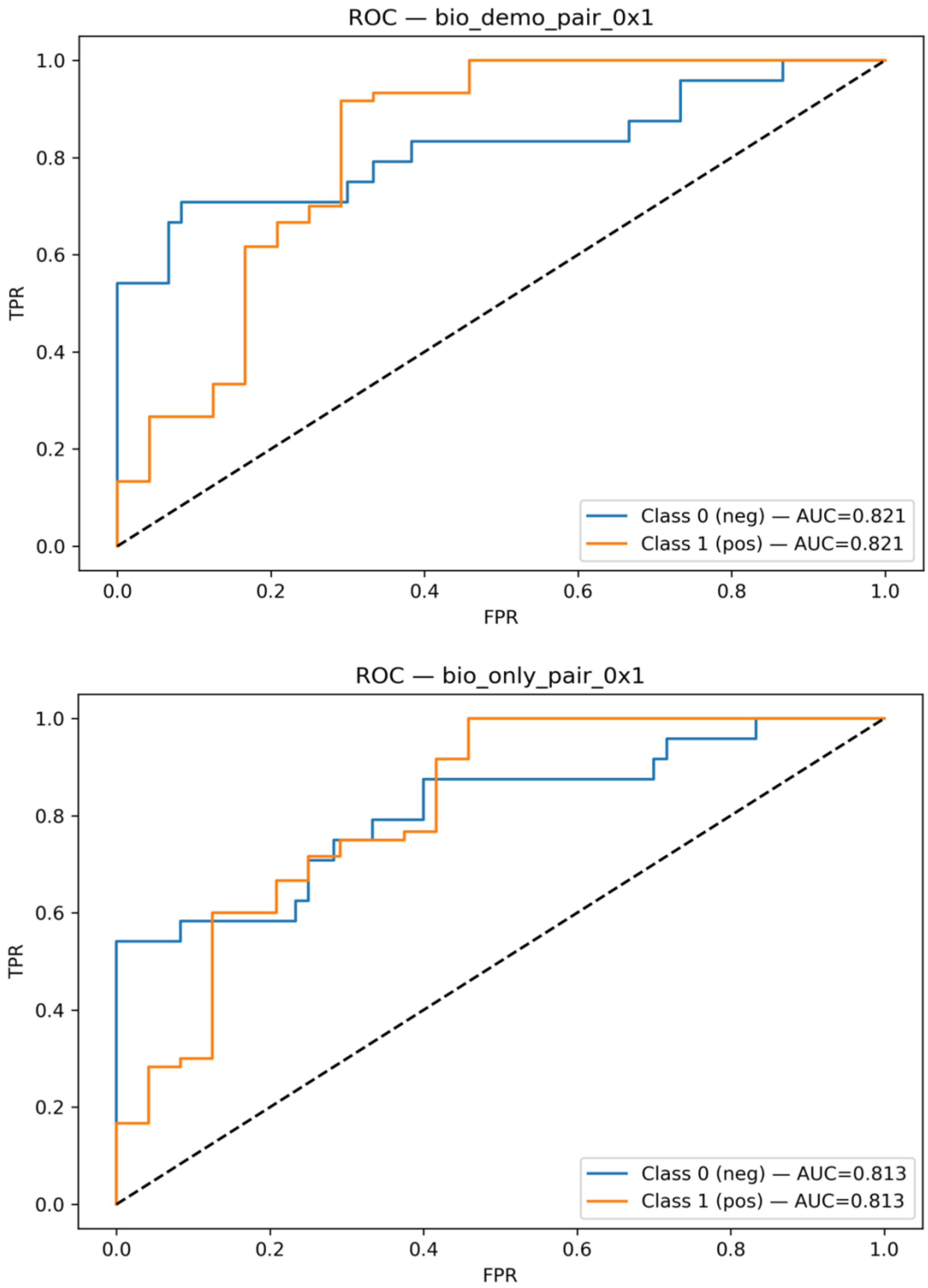

Supplementary Figure S6: ROC curves for the 0×1 classification task. TPR: True Positive Rate; FPR: False Positive Rate.

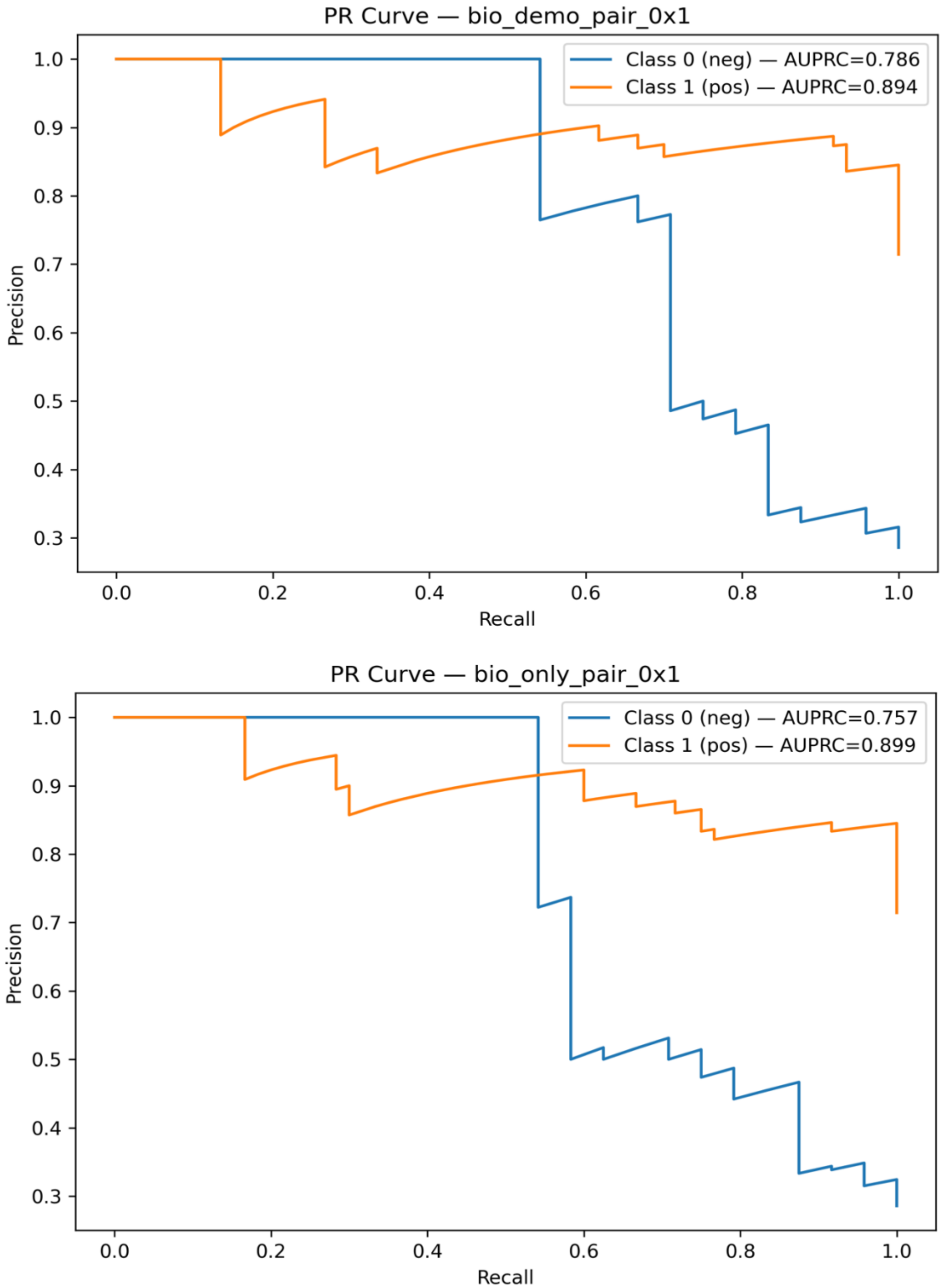

Supplementary Figure S7: Precision–recall curves for the 0×1 classification task.

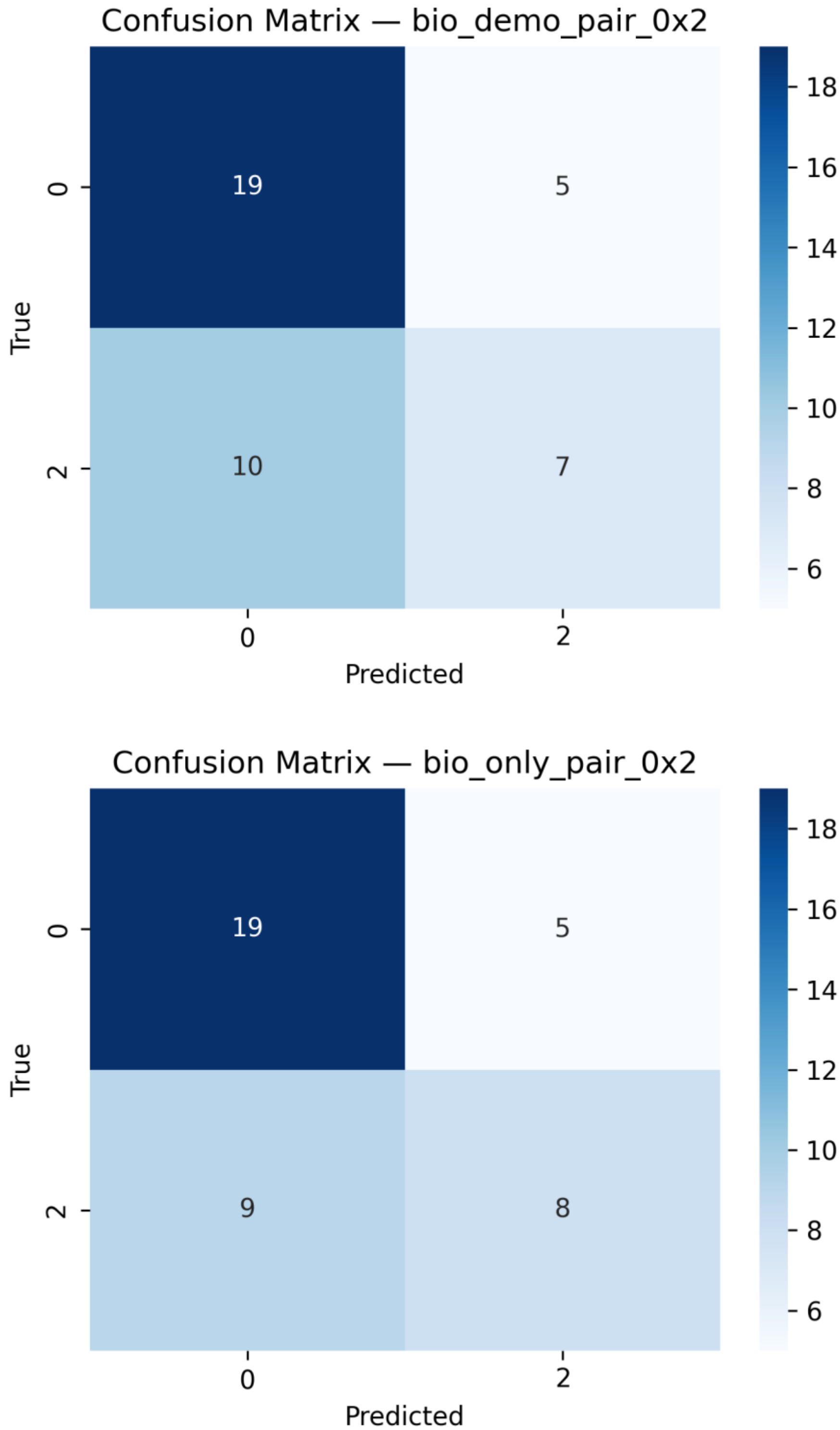

Supplementary Figure S8: Confusion matrices for the 0×2 classification task.

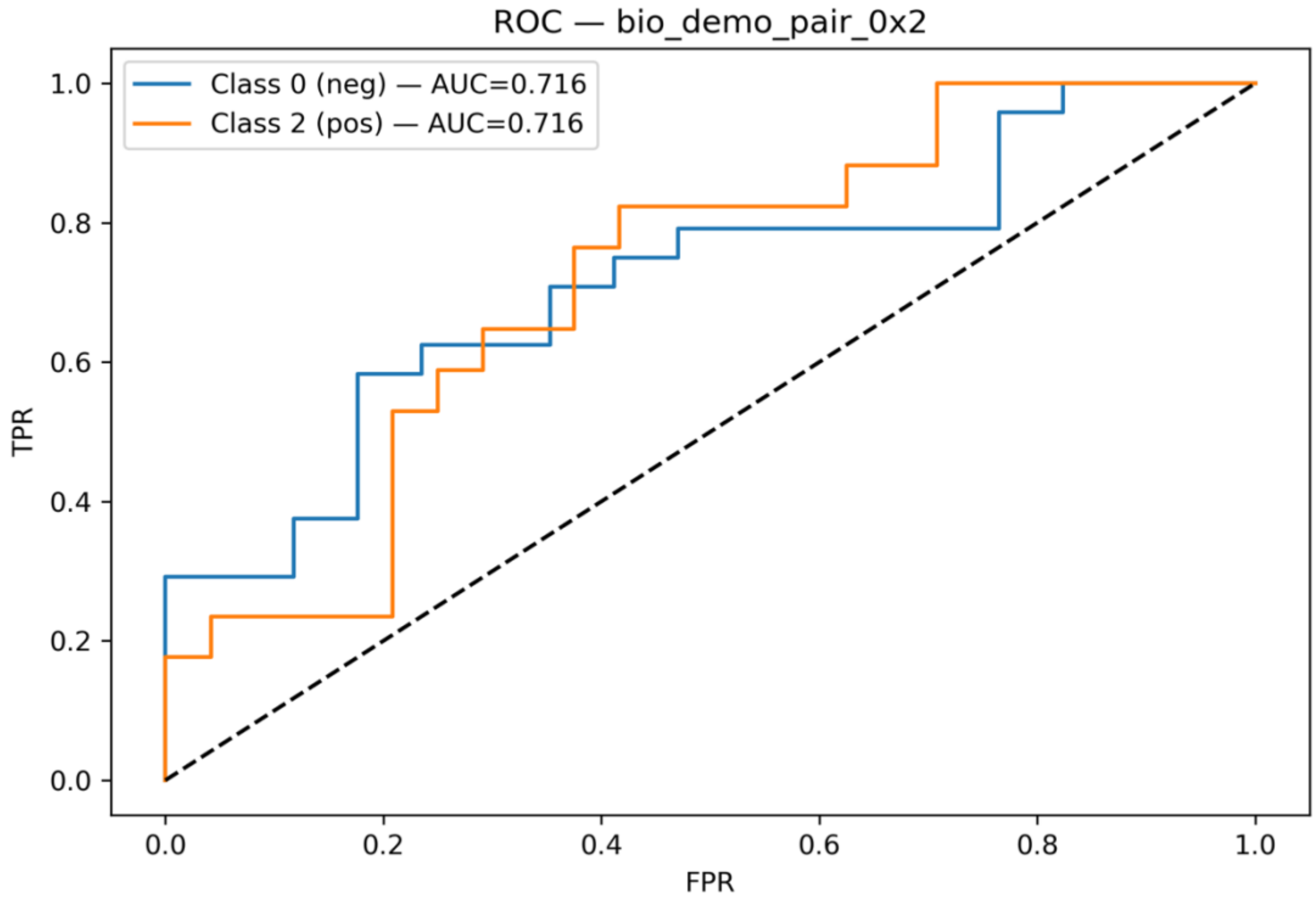

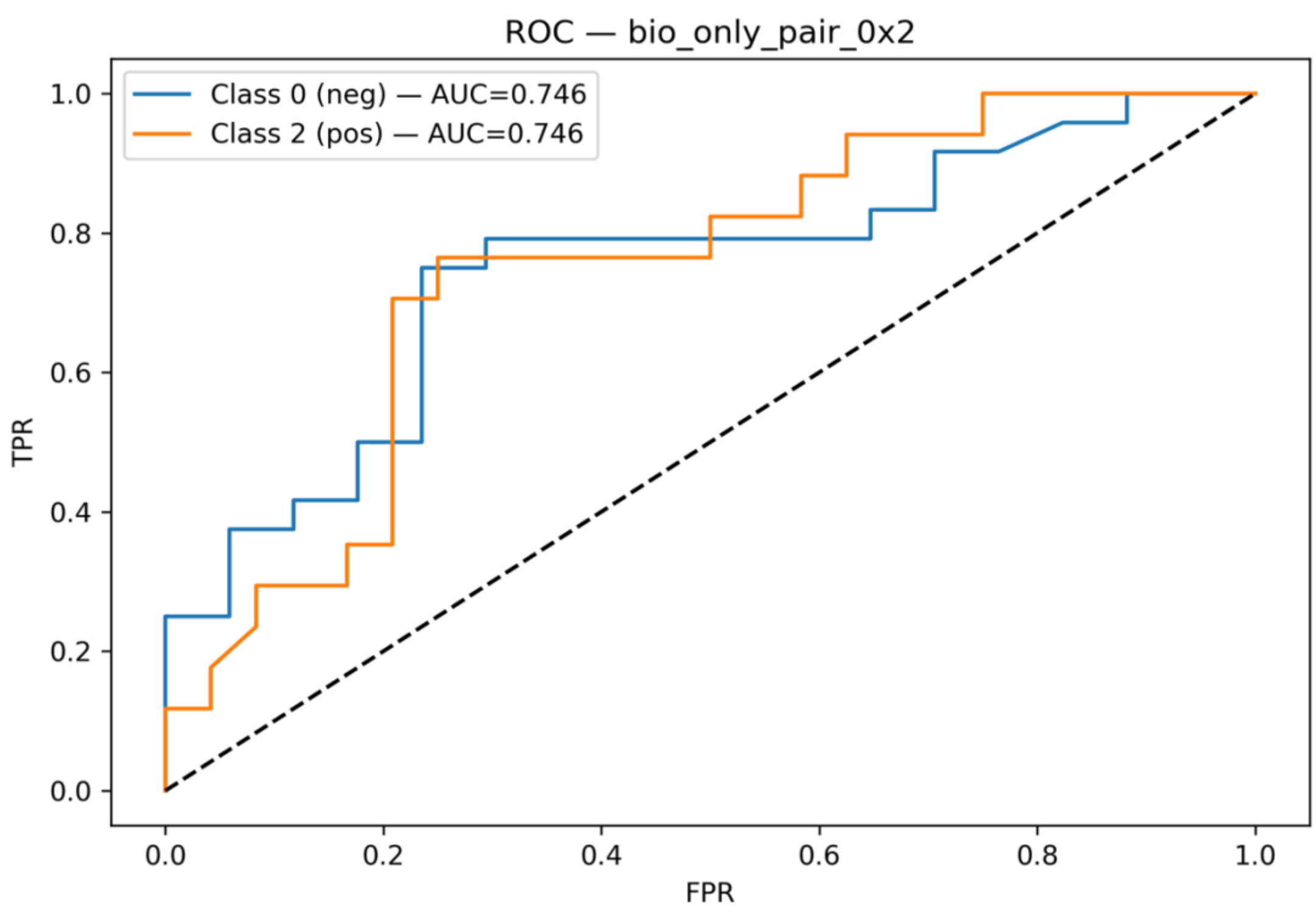

Supplementary Figure S9: ROC curves for the 0×2 classification task. TPR: True Positive Rate; FPR: False Positive Rate.

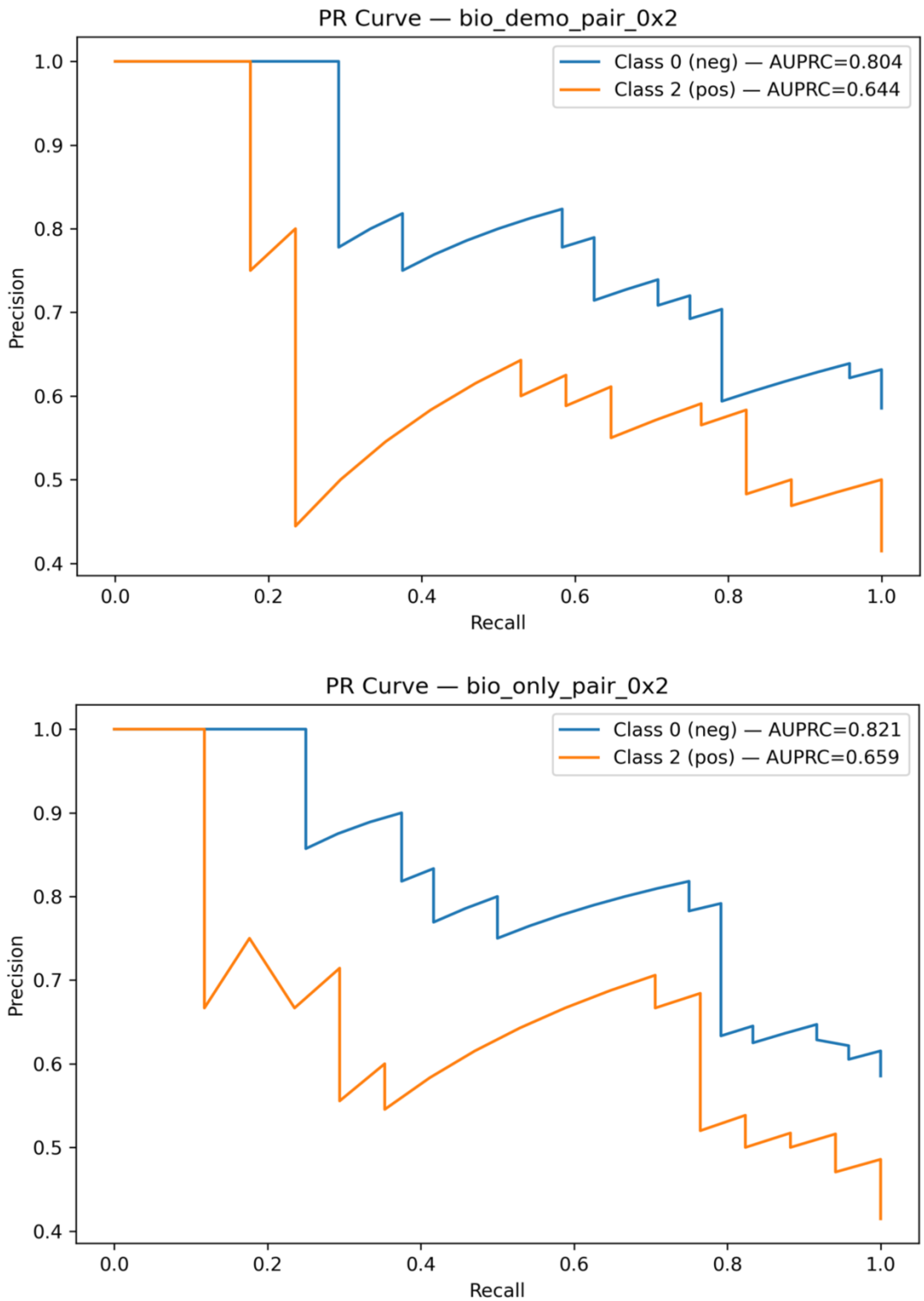

Supplementary Figure S10: Precision–recall curves for the 0×2 classification task.

S3.4 SHAP Interpretability and Stability

This section summarizes the SHAP-based interpretability analyses performed across all outer folds of the nested cross-validation framework. Two complementary perspectives are provided:

1. Fold-wise SHAP rank variability: heatmaps illustrating how the rank position of each biomarker changes across folds, thereby quantifying the stability of feature-importance ordering.
2. SHAP Top-10 stability: barplots reporting how frequently each biomarker appears among the ten most influential features across the five outer folds.

Together, these analyses offer a model-agnostic, fold-resolved assessment of which biomarkers provide a reproducible predictive signal rather than fold-specific variability. Results are reported for the three-class task and for the two binary pairwise tasks (Control vs. TBI; Control vs. Orthopedic Injury), and for both modeling scenarios: (i) biomarkers + age/sex (bio_demo) and (ii) biomarkers only (bio_only).

All SHAP computations were performed exclusively on the held-out outer test folds, ensuring fully leakage-free interpretability.

S3.4.1 Three-Class Classification: SHAP-Based Interpretability.

S3.4.1.1 Fold-Wise SHAP Rank Variability (Figures S11–S13).

Figures S11–S13 display SHAP rank heatmaps for Classes 0, 1, and 2, respectively. Each heatmap reports, for each biomarker, its rank position in each outer fold. Darker tones indicate top-tier rankings, while lighter tones reflect lower importance or greater fluctuation across folds. Across both bio_demo and bio_only, a restricted subset of biomarkers is among the most influential features: LIGHT is highly stable for Classes 0 and 1, and IL-5 consistently ranks highest for Class 2. These biomarkers maintain high ranks across nearly all folds, suggesting a robust discriminative signal despite the modest sample size. The similarity between bio_demo and bio_only rank profiles further indicates that demographic features do not substantially alter the underlying biomarker-driven predictive structure

S3.4.1.2 SHAP Top-10 Stability Across Folds (Figures S14–S16).

Figures S14–S16 illustrate the frequency with which each biomarker appears among the Top-10 most influential features across the five outer folds. Frequencies approaching 5 denote excellent stability. Across both feature configurations, several biomarkers recur within the Top-10, underscoring their role as reliable discriminative features. The most recurrent biomarkers across folds include LIGHT, IL-5, sCD40L, IL-1β, MPO, GRO-KC, IL-3, EGF, MMP-9, and Fractalkine. This high recurrence confirms that these biomarkers provide a stable, fold-robust signal for the

three-class classification problem. When included, demographic variables contribute interpretably (e.g., 'age' in Class 0, Figure S14), but exert a secondary influence on the overall stability profile. Lower-frequency biomarkers likely represent fold-specific fluctuations or minor contributions to discrimination.

S3.4.2 Binary Classification: Control vs. TBI (0×1).

S3.4.2.1 Fold-Wise SHAP Rank Variability (Figures S17–S18).

Figures S17 and S18 display the SHAP rank heatmaps for Class 0 (Control) and Class 1 (TBI). Across all resampling folds, a core set of inflammatory and neuroinjury-related biomarkers consistently occupies top-tier rankings, particularly within the bio_only configuration. The addition of demographic variables (bio_demo) results in only marginal shifts in rank distribution, leaving the primary biomarker-driven signal largely intact. In this 0×1 pairwise comparison, LIGHT, GRO-KC, and MPO emerge as the most influential features. Their sustained cross-fold consistency underscores a robust and reproducible biological signature distinguishing TBI from controls, whereas other markers exhibit higher rank variability, likely attributable to biological heterogeneity or sampling noise.

S3.4.2.2 SHAP Top-10 Stability Across Folds (Figures S19–S20).

Figures S19 and S20 illustrate the Top-10 stability frequencies for Classes 0 (Control) and 1 (TBI). Several biomarkers recur across all five outer folds (frequency = 5), demonstrating robust and reproducible importance for distinguishing TBI from controls. In contrast, demographic variables appear less frequently, underscoring their auxiliary role in the predictive model. Highly stable biomarkers include LIGHT, GRO-KC, and MMP-9, which consistently rank within the Top-10 across both modeling scenarios. Features with lower recurrence likely reflect fold-specific variations rather than systematic contributions. Overall, the TBI pairwise comparison provides the most precise and stable biomarker importance profile among all evaluated tasks

S3.4.3 Binary Classification: Control vs. Orthopedic Injury (0×2)

S3.4.3.1 Fold-Wise SHAP Rank Variability (Figures S21–S22).

Figures S21 and S22 present the SHAP rank heatmaps for Class 0 (Control) and Class 2 (Orthopedic Injury), respectively. In contrast to the 0×1 task, the 0×2 pairwise comparison reveals distinct ranking patterns, reflecting the divergent biological signatures of orthopedic injury versus TBI. While specific biomarkers—most notably GCSF—exhibit relatively stable top-tier positions, the overall rank variability is markedly higher than in the TBI task. This increased fluctuation underscores the challenges in discrimination, likely due to the partial overlap between systemic inflammatory responses in orthopedic trauma and the profiles of healthy controls.

S3.4.3.2 SHAP Top-10 Stability Across Folds (Figures S23–S24).

Figures S23 and S24 report Top-10 stability frequencies for the 0×2 task. Several biomarkers exhibit frequencies of 4–5, indicating meaningful but moderate stability across folds. Comparisons between bio_demo and bio_only show that demographic variables do not substantially alter stability patterns, as the discriminatory signal remains predominantly biological. BDNF, GCSF, MDC, and TFG-α are among the most stable features, though fewer biomarkers exhibit high recurrence rates than in the 0×1 task. This pattern suggests that separating controls from orthopedic injury involves a weaker or more heterogeneous biomarker signal than the signature observed for TBI.

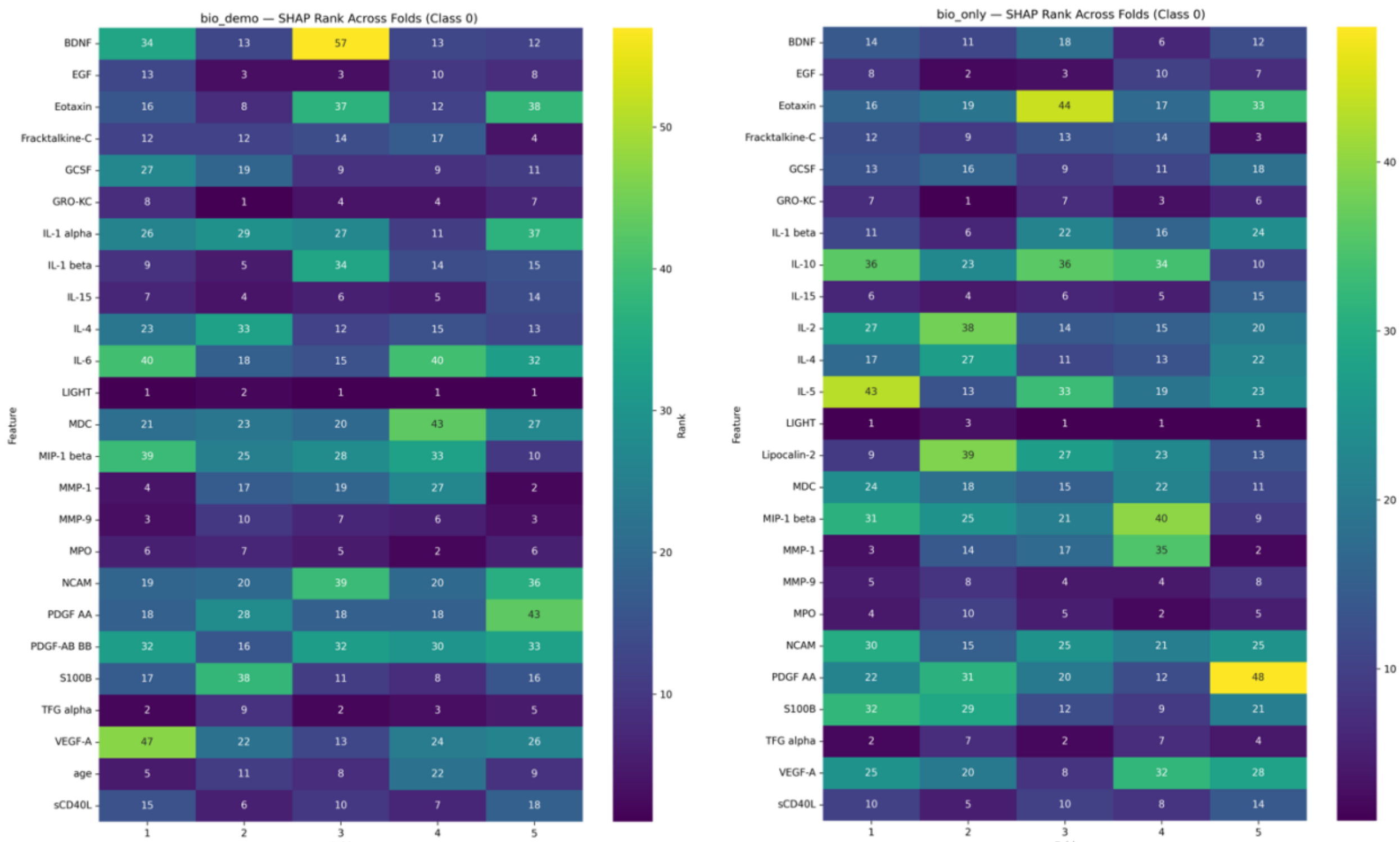

Supplementary Figure S11: SHAP rank variability across outer folds for Class 0 in the three-class classification task under both modeling scenarios (bio_demo and bio_only). Each cell denotes the rank position of a biomarker in a given fold.

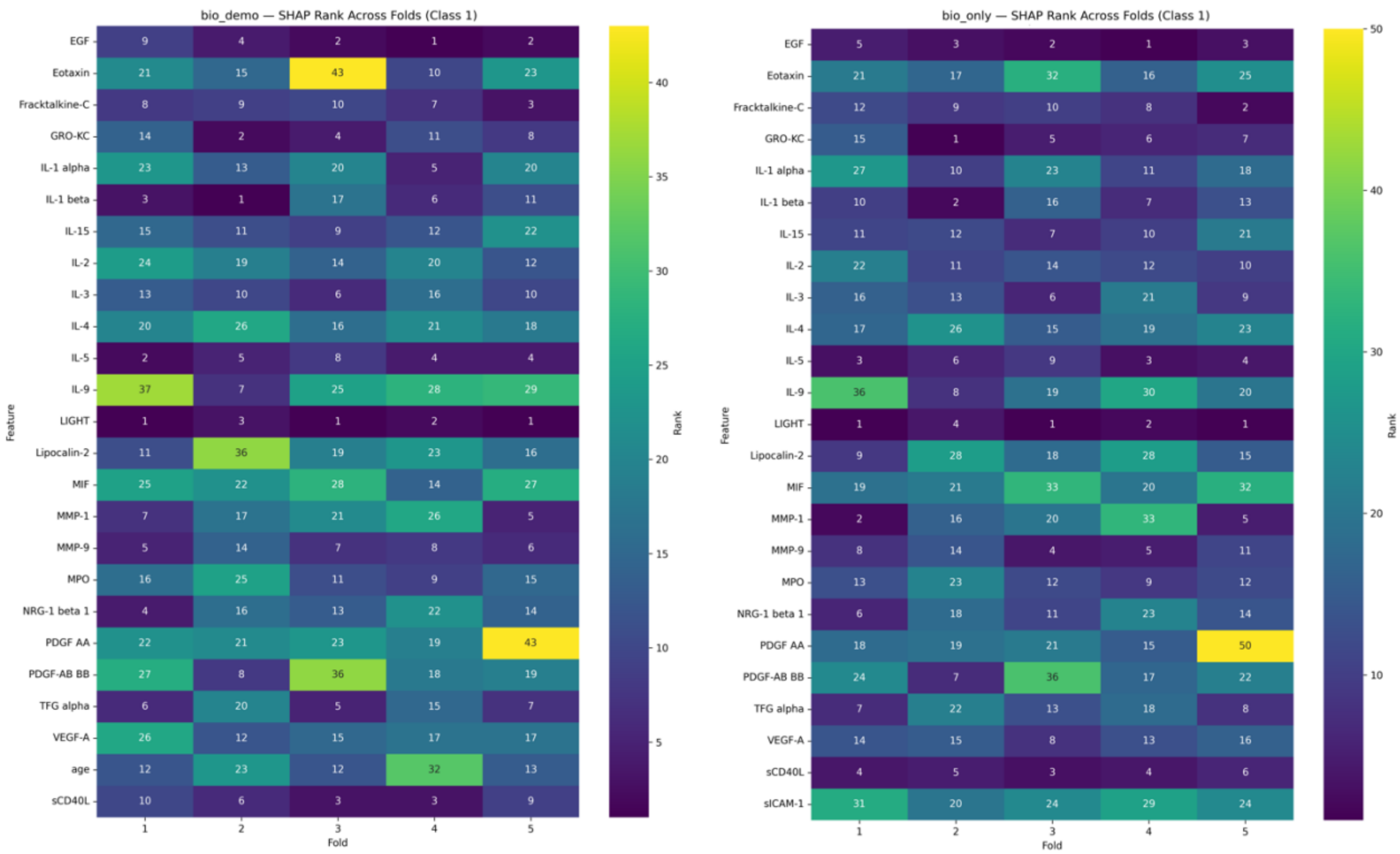

Supplementary Figure S12: SHAP rank variability across outer folds for Class 1 in the three-class classification task under both modeling scenarios.

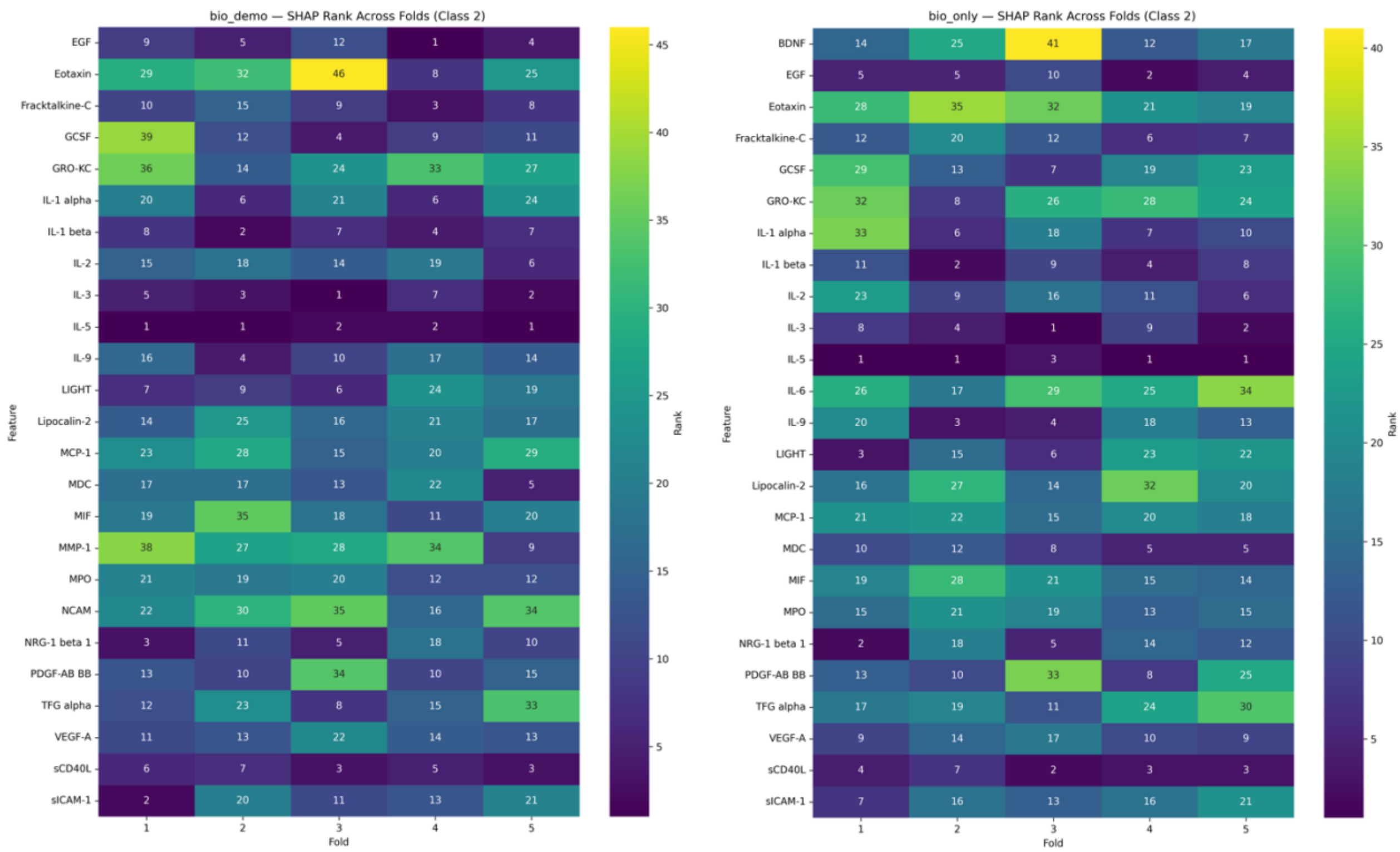

Supplementary Figure S13: SHAP rank variability across outer folds for Class 2 in the three-class classification task under both modeling scenarios.

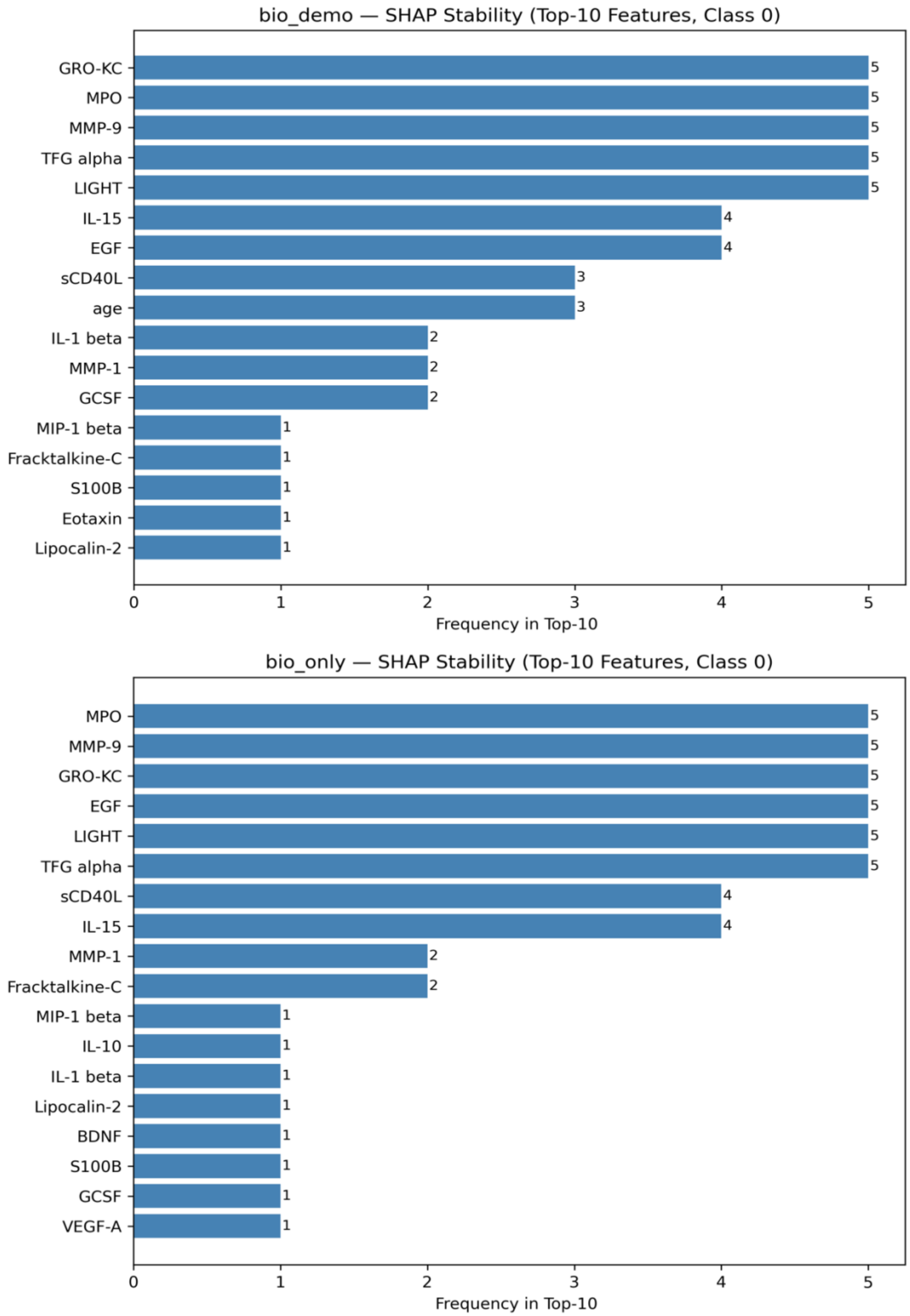

Supplementary Figure S14: SHAP Top-10 stability for Class 0 across outer folds under both modeling scenarios. Bars represent the frequency with which each biomarker appeared among the ten most influential features.

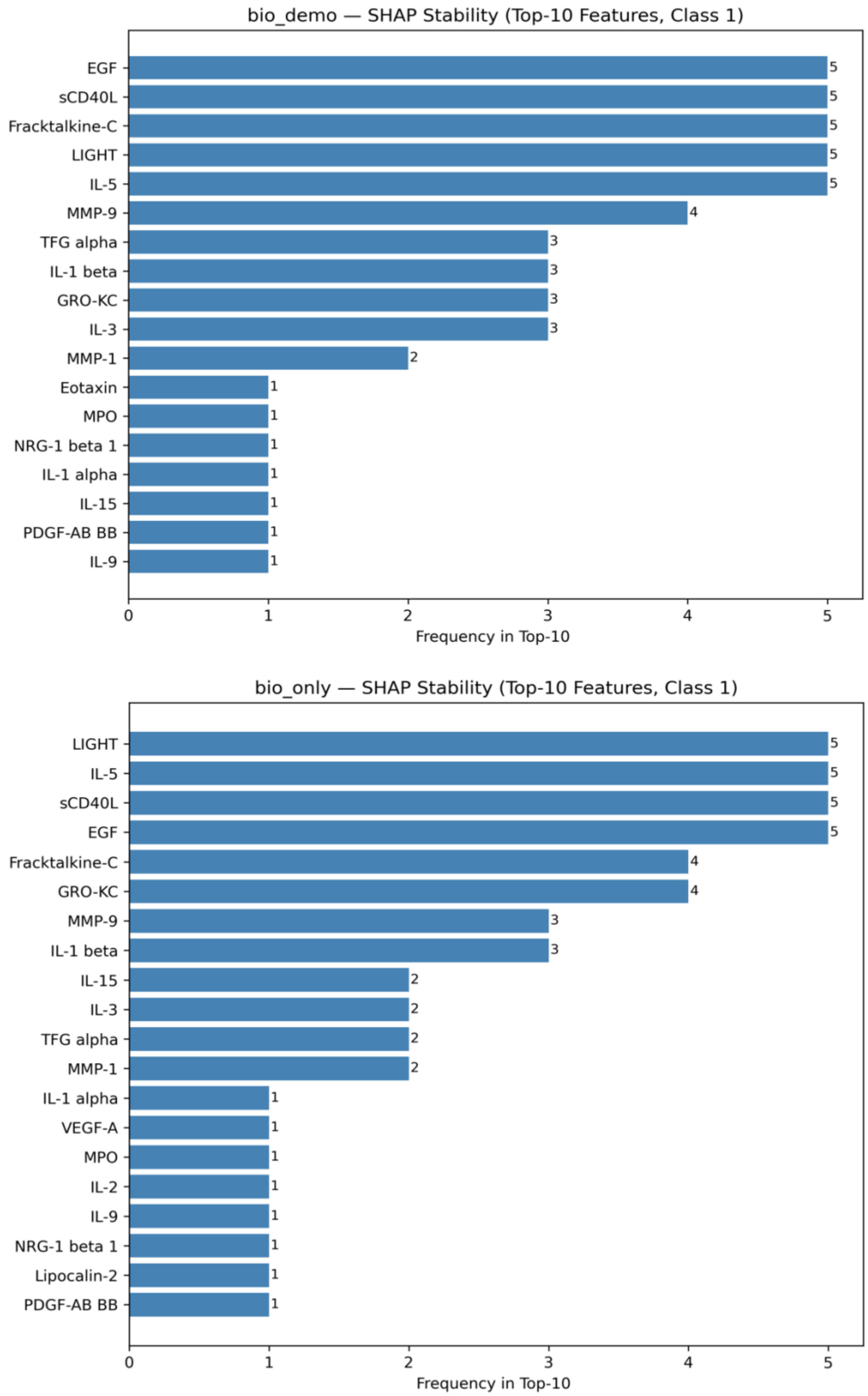

Supplementary Figure S15: SHAP Top-10 stability for Class 1 across outer folds under both modeling scenarios.

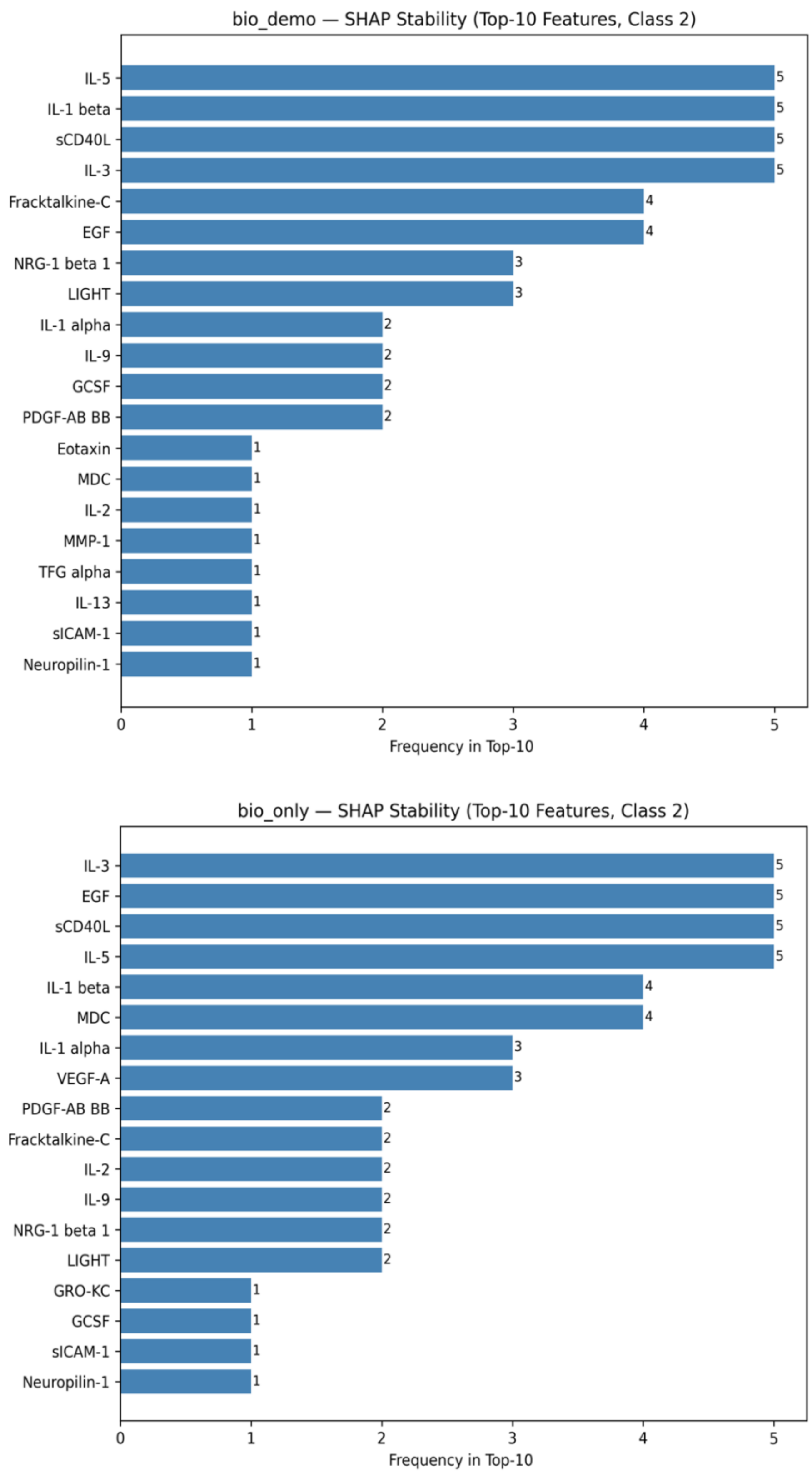

Supplementary Figure S16: SHAP Top-10 stability for Class 2 across outer folds under both modeling scenarios.

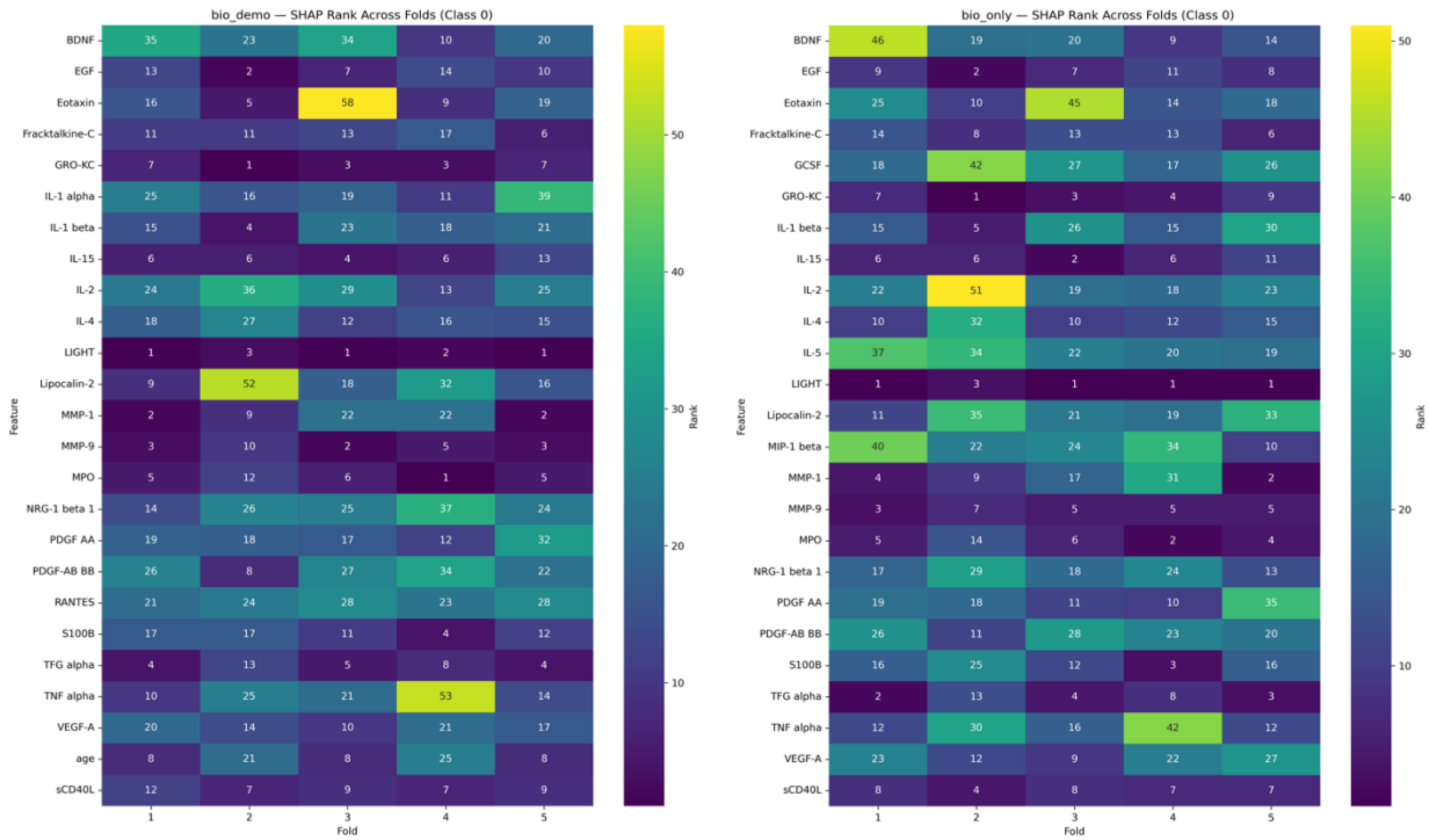

Supplementary Figure S17: SHAP rank variability across outer folds for Class 0 in the Control vs. TBI (0×1) classification task.

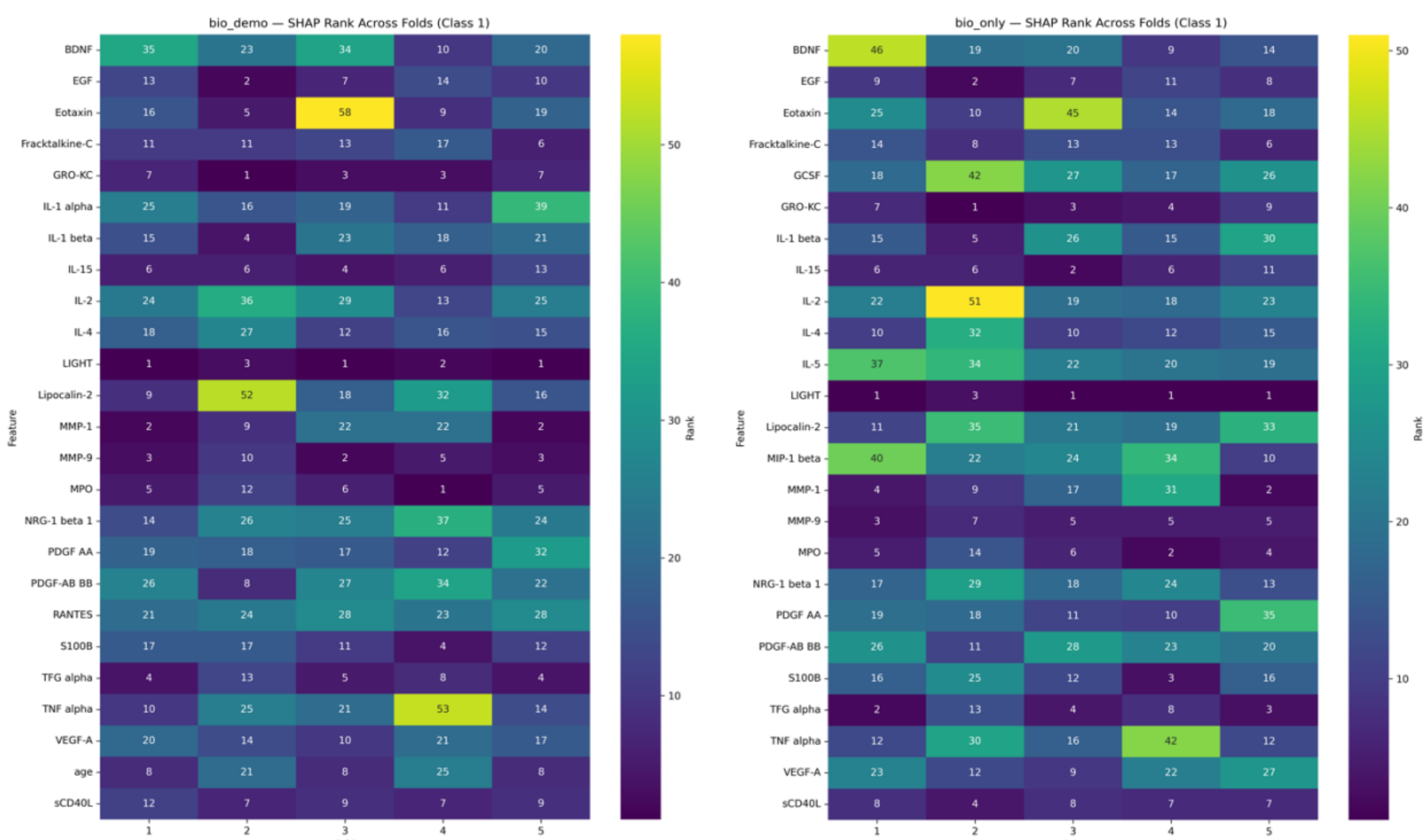

Supplementary Figure S18: SHAP rank variability across outer folds for Class 1 in the Control vs. TBI (0×1) classification task.

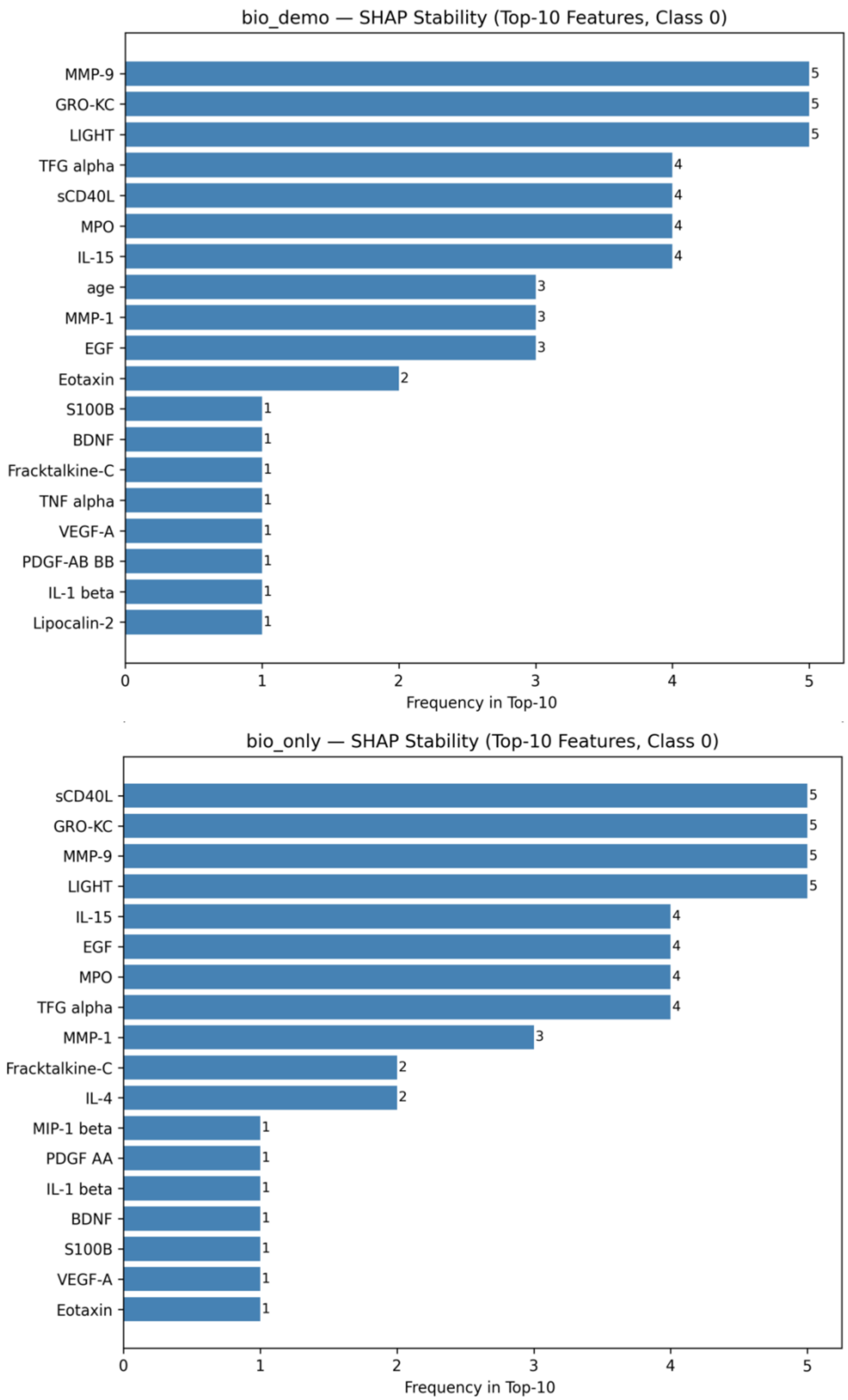

Supplementary Figure S19: SHAP Top-10 stability for Class 0 in the Control vs. TBI (0×1) task across outer folds.

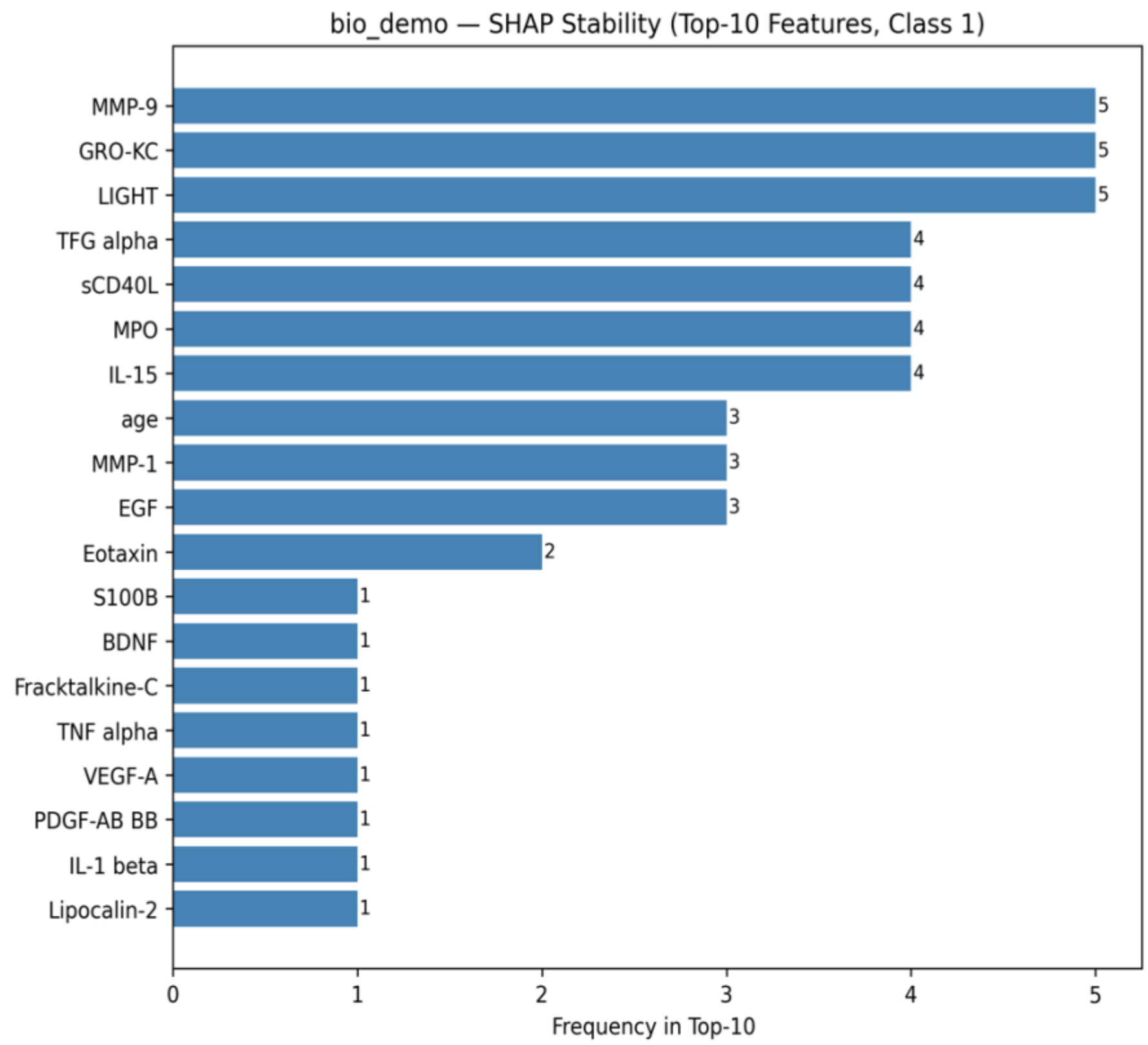

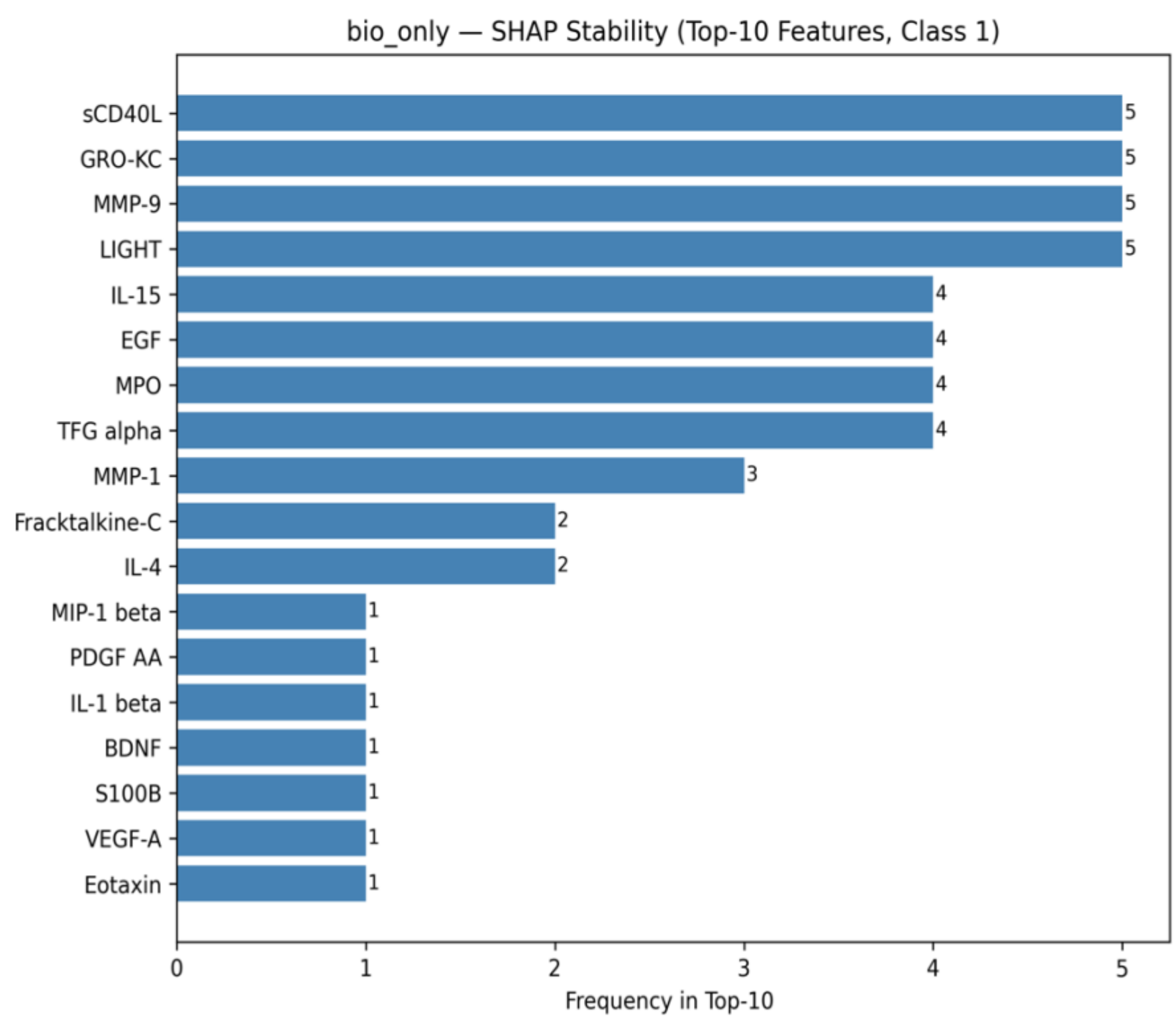

Supplementary Figure S20: SHAP Top-10 stability for Class 1 in the Control vs. TBI (0×1) task across outer folds.

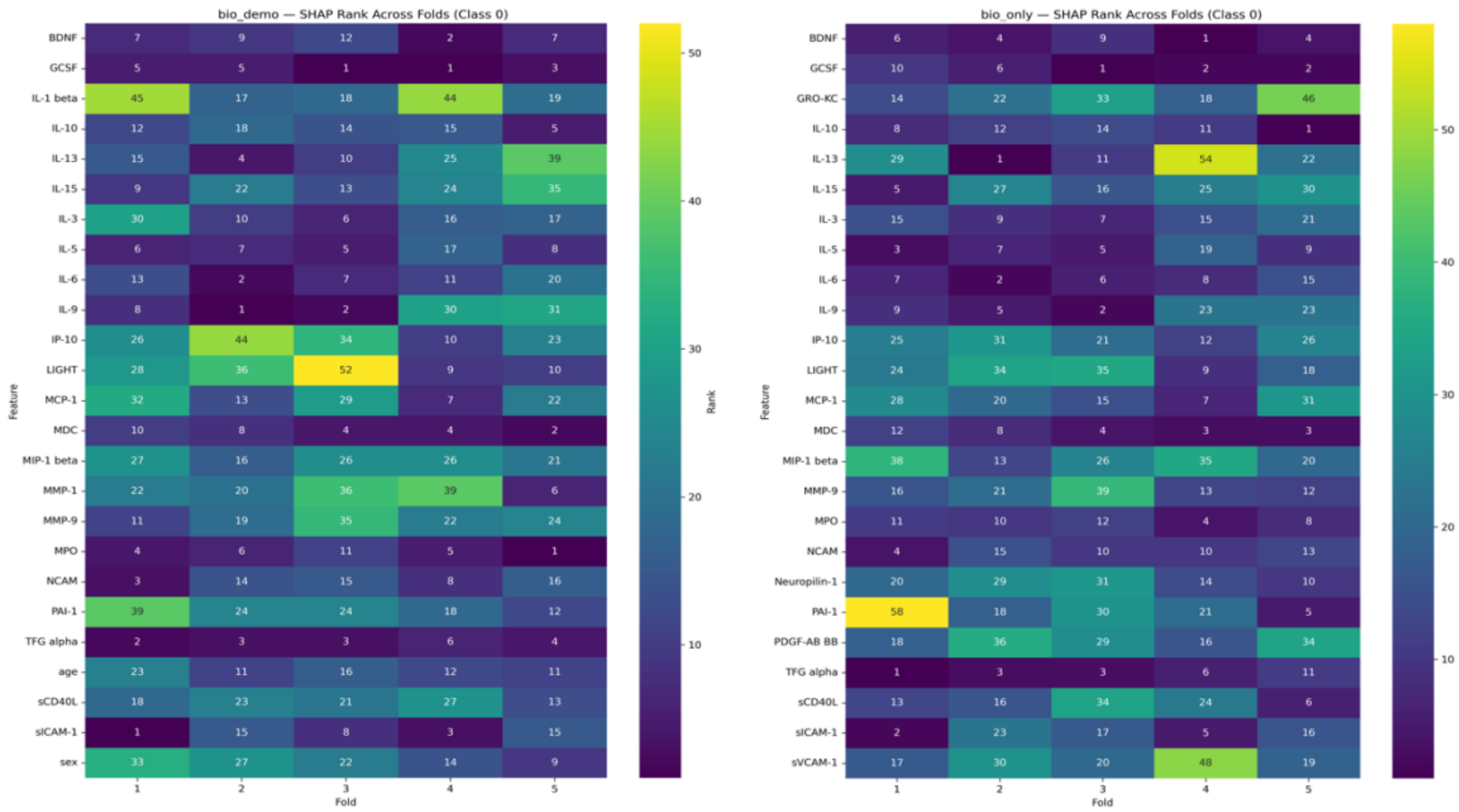

Supplementary Figure S21: SHAP rank variability across outer folds for Class 0 in the Control vs. Orthopedic Injury (0×2) classification task.

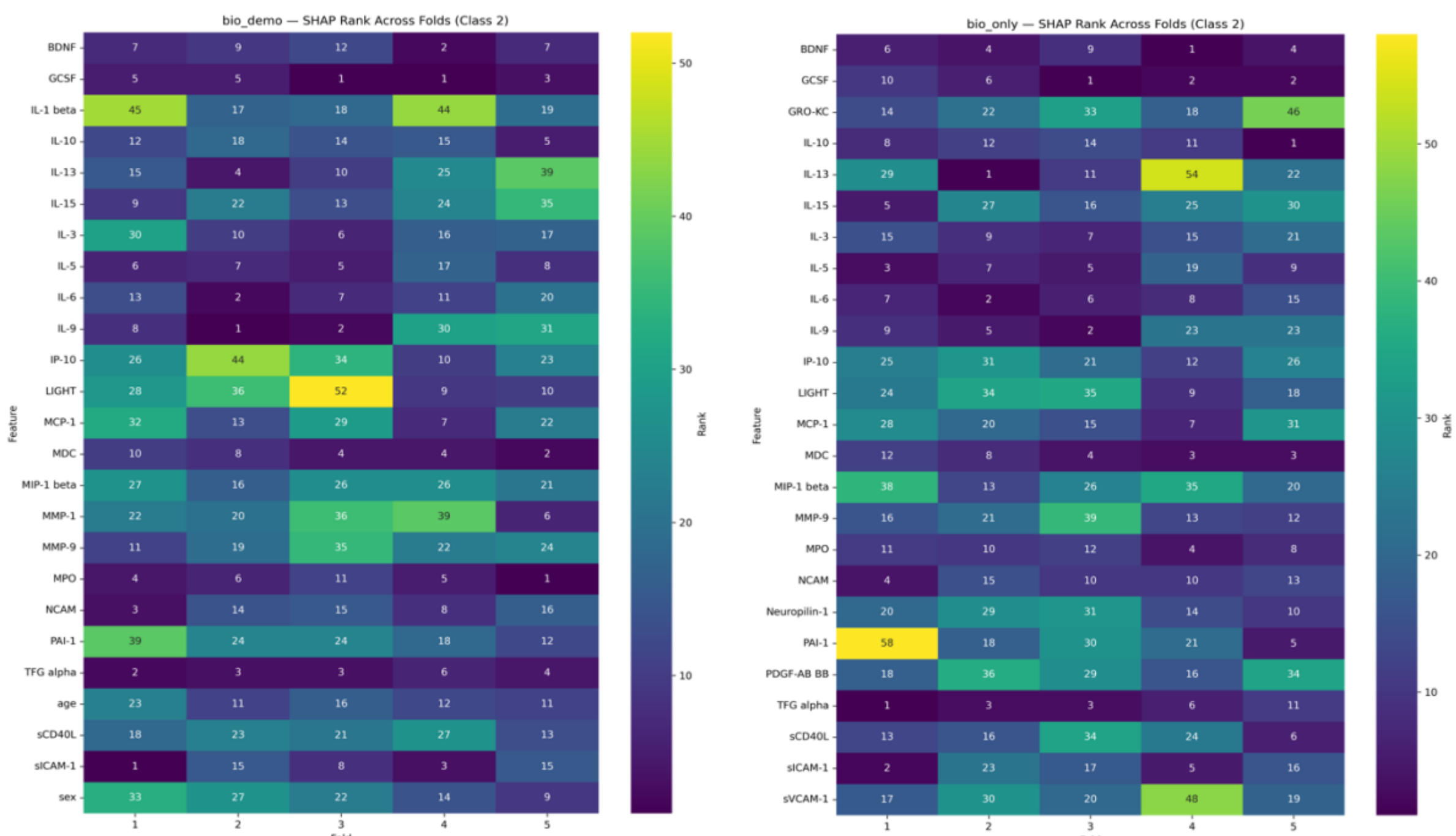

Supplementary Figure S22: SHAP rank variability across outer folds for Class 2 in the Control vs. Orthopedic Injury (0×2) classification task.

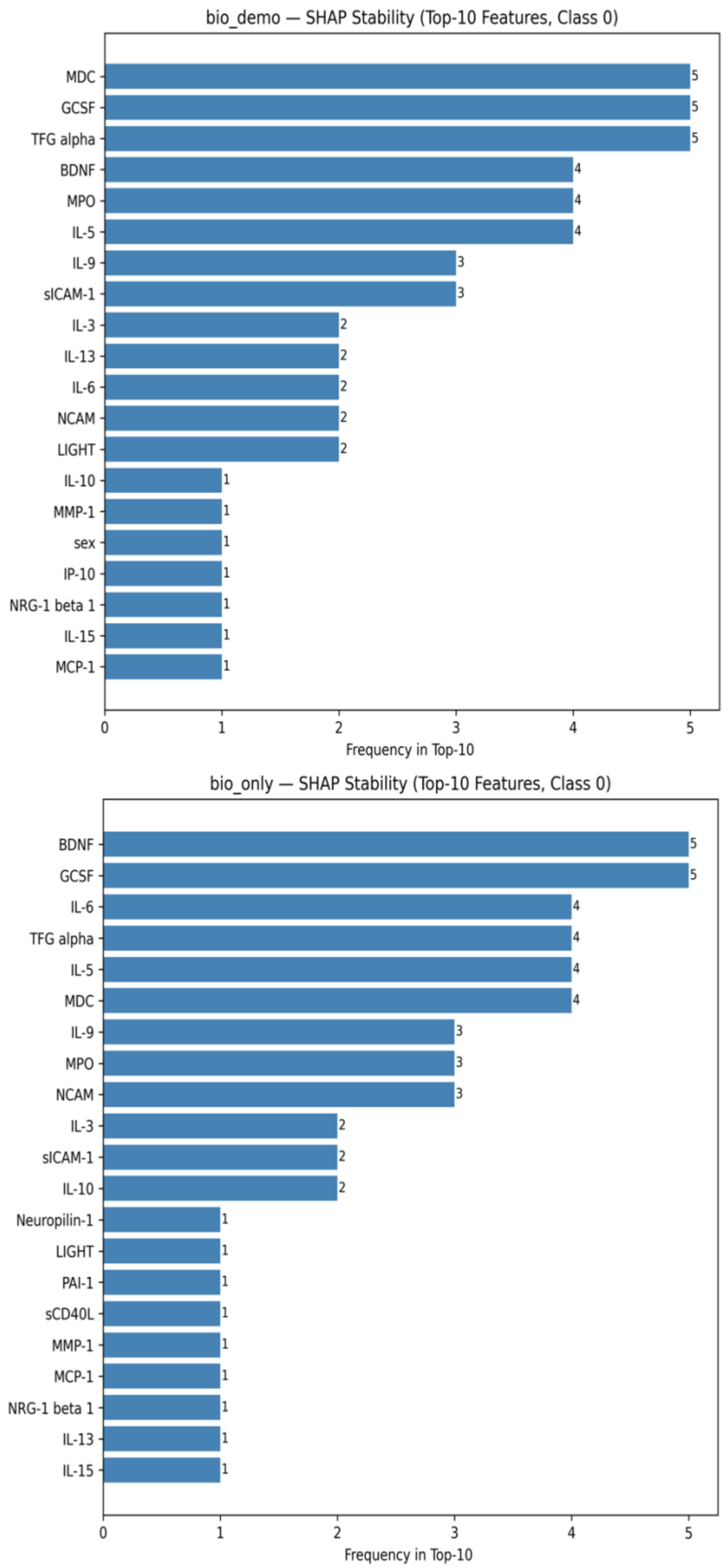

Supplementary Figure S23: SHAP Top-10 stability for Class 0 in the Control vs. Orthopedic Injury (0×2) task across outer folds.

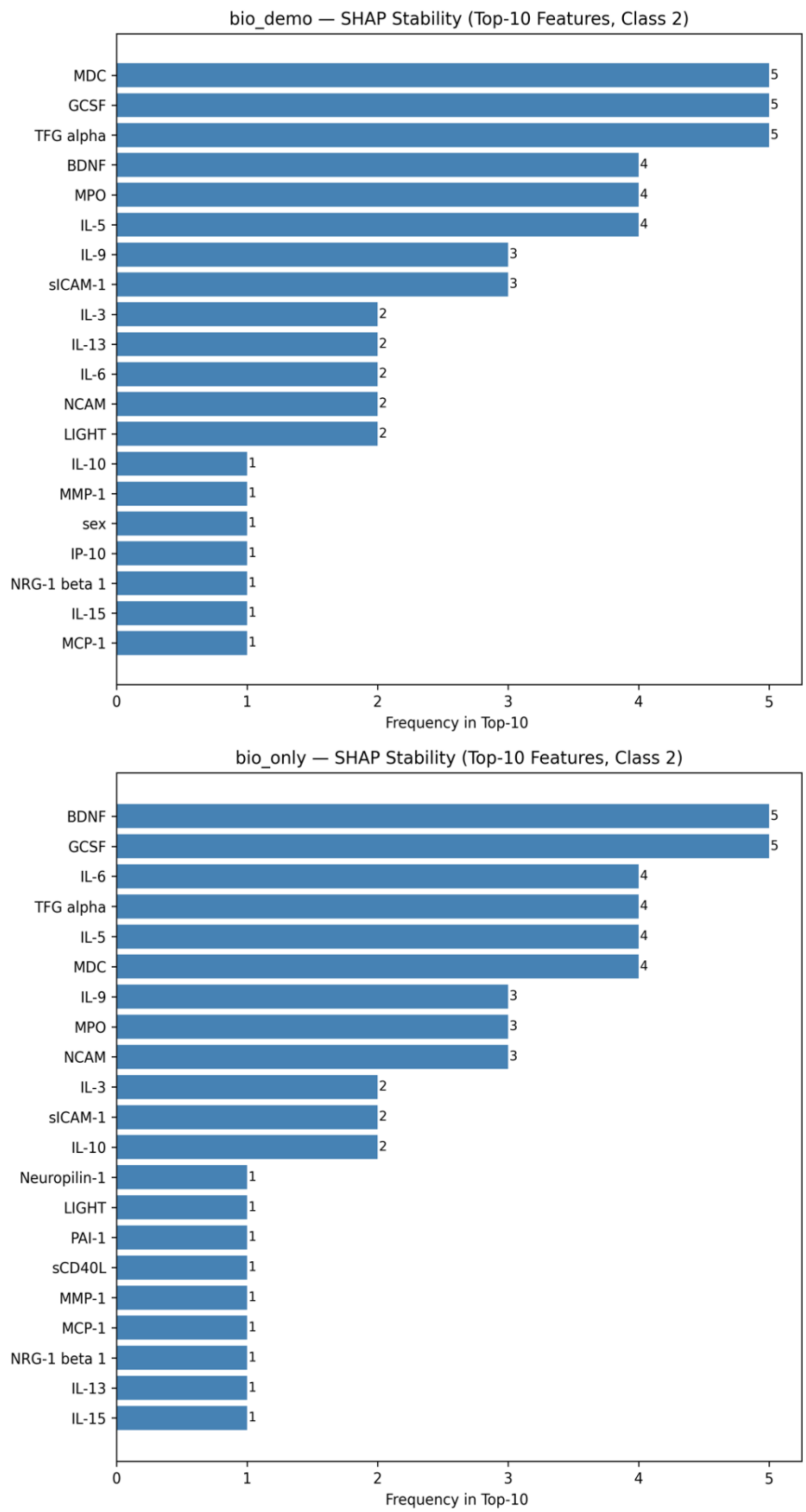

Supplementary Figure S24: SHAP Top-10 stability for Class 2 in the Control vs. Orthopedic Injury (0×2) task across outer folds.

Additional Bibliography:

Chen X. et al. Machine Learning in Clinical Biomarker Research. Nature Medicine Reviews (2021).

Harris, C. R., Millman, K. J., van der Walt, S. J., et al. (2020). Array programming with NumPy. *Nature*, 585, 357–362.

Hunter, J. D. (2007). Matplotlib: A 2D graphics environment. *Computing in Science & Engineering*, 9(3), 90–95.

Kuhn, M., Johnson, K. Applied Predictive Modeling. Springer (2013).

McKinney, W. (2010). Data Structures for Statistical Computing in Python. In *Proceedings of the 9th Python in Science Conference* (pp. 56–61).
